# Self-assembling α,γ-cyclic peptides that generate cavities with tunable properties†
†Electronic supplementary information (ESI) available: Synthetic experimental details, DFT calculation details, additional figures (relevant NMR data) and schemes (synthetic schemes for peptide and amino acid preparation). CCDC 1400134. For ESI and crystallographic data in CIF or other electronic format see DOI: 10.1039/c5sc03187g



**DOI:** 10.1039/c5sc03187g

**Published:** 2015-09-14

**Authors:** Nuria Rodríguez-Vázquez, Rebeca García-Fandiño, Manuel Amorín, Juan R. Granja

**Affiliations:** a Singular Research Centre in Chemical Biology and Molecular Materials , (CIQUS) , Organic Chemistry Department , University of Santiago de Compostela (USC) , 15782 Santiago de Compostela , Spain

## Abstract

The design and synthesis of β-sheet-based self-assembling cyclic peptides with tunable cavities is described. The incorporation of a γ-amino acid with a hydroxyl group at C2 allows the incorporation of different groups that modify the internal properties of the resulting dimeric ensemble. These dimers can entrap different guests depending on the properties of the group at C2. The guest defines the geometry of the resulting aggregate.

## Abstract 2

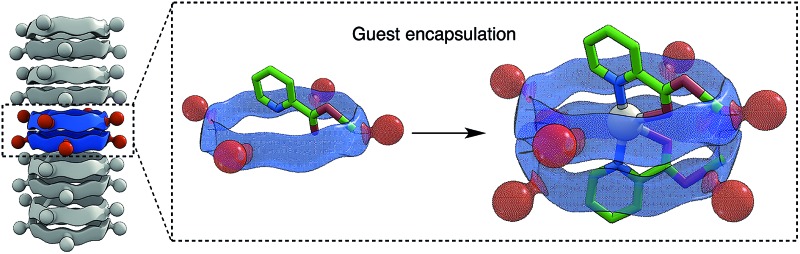
The design and synthesis of self-assembling cyclic peptides with tunable cavity properties is described, allowing the incorporation of guests with different features.

## Introduction

In recent years peptides have emerged as powerful tools for the design of novel nanobiomaterials because of their exceptional characteristics based on their unique folding and self-assembly properties.^1^ Peptide nanotubes are tubular structures formed by peptide components.^2^ Self-assembling cyclic peptide nanotubes (SCPNs) are a subclass of this type of material formed by the stacking of cyclic components in a pseudo-flat conformation^3^ and one of the main advantages of these systems is the rigorous control of the internal diameter.^4^ In the last few years we have been working with cyclic peptides (CPs) that contain 3-aminocycloalkanecarboxylic acids, broadening the types of SCPNs available and also allowing functionalization of their cavities.^5^ Selectively *N*-methylated CPs are SCPN models that allow the study of nanotube properties which is not easily achieved with the full supramolecular structure.^6^ Here we present a new SCPN model in which the properties of the internal cavity can be modified. The resulting dimers entrap different guests, such as metal ions, alcohols or dicarboxylic acids.

Cyclic γ-amino acids are very useful building blocks for the internal functionalization of peptide nanotubes.^7^ We recently reported the synthesis of a 3-hydroxytetrahydrofuran (Ahf) derivative **1** and used this compound in the synthesis of a cyclic tetrapeptide.^8^ This peptide could form two non-equivalent dimers but the presence of the hydroxyl group directed the equilibrium towards the one in which the two hydroxyl groups participated in hydrogen-bonding interactions.^8,9^ The resulting ensemble had a very small internal cavity which was unable to interact with other molecules. In the work described here we extended the study to larger CPs, evaluating their ensemble cavity properties that allow the incorporation of a variety of guests with different chemical properties.

## Results and discussion

### Synthetic studies and dimer characterization

**Scheme 1:**
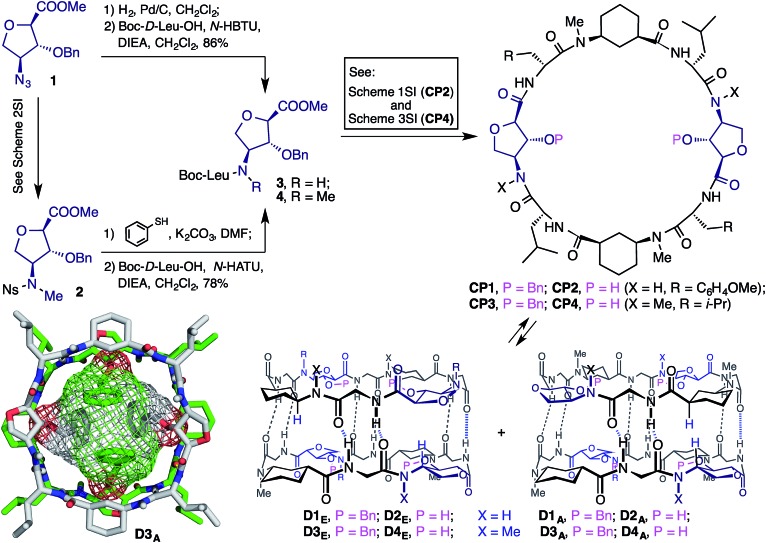
Structure of the α,γ-cyclic peptides (**CP1–4**) and their corresponding dimers. Bottom-left corner: X-ray structure of **D3_A_**.

**Fig. 1:**
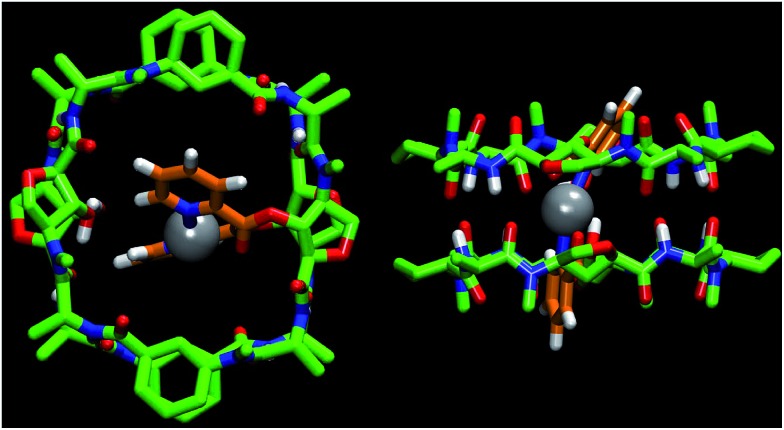
Computational structure of **s-D5_E_ ⊃ Ag** showing the incorporation of the silver ion (gray color) within the dimer cavity coordinating the two picolinates (orange). All hydrogens, except those from the NHs, OHs and the pyridines, have been removed for clarity.

Following the aim of studying the internal cavity properties of cyclic peptide precursors of SCPNs that generate assemblies with functionalized cavities, the octapeptide **CP2** was designed (Scheme 1). This peptide contains two Ahf residues and would form large hydrophilic cavities with *C*_2_ symmetry. The synthesis of the peptide was carried out in solution following a strategy similar to that reported previously (see Scheme 1SI in the ESI†).^8^ Considering the *C*_2_ symmetry, **CP2** can form two non-equivalent dimers that are called eclipsed (**D2_E_**), *i.e.*, the form in which the two Ahf residues lie on top of each other, and alternated (**D2_A_**), *i.e.*, the form in which the 3-aminocyclohexanecarboxylic acid (Ach) and Ahf residues are paired. Unfortunately, neither of the two peptides (**CP2** and protected **CP1**) formed the corresponding dimers. Instead they adopted a folded conformation in which the Ahf amide proton was hydrogen bonded to its own carbonyl group.^10,11^ The singlet signals in the ^1^H NMR spectra of the vicinal protons at C2 (Hα) and C3 (Hβ) suggest that they are perpendicularly oriented, adopting a conformation that prevents dimer formation (Fig. 1SI, ESI†).

**Scheme 2:**
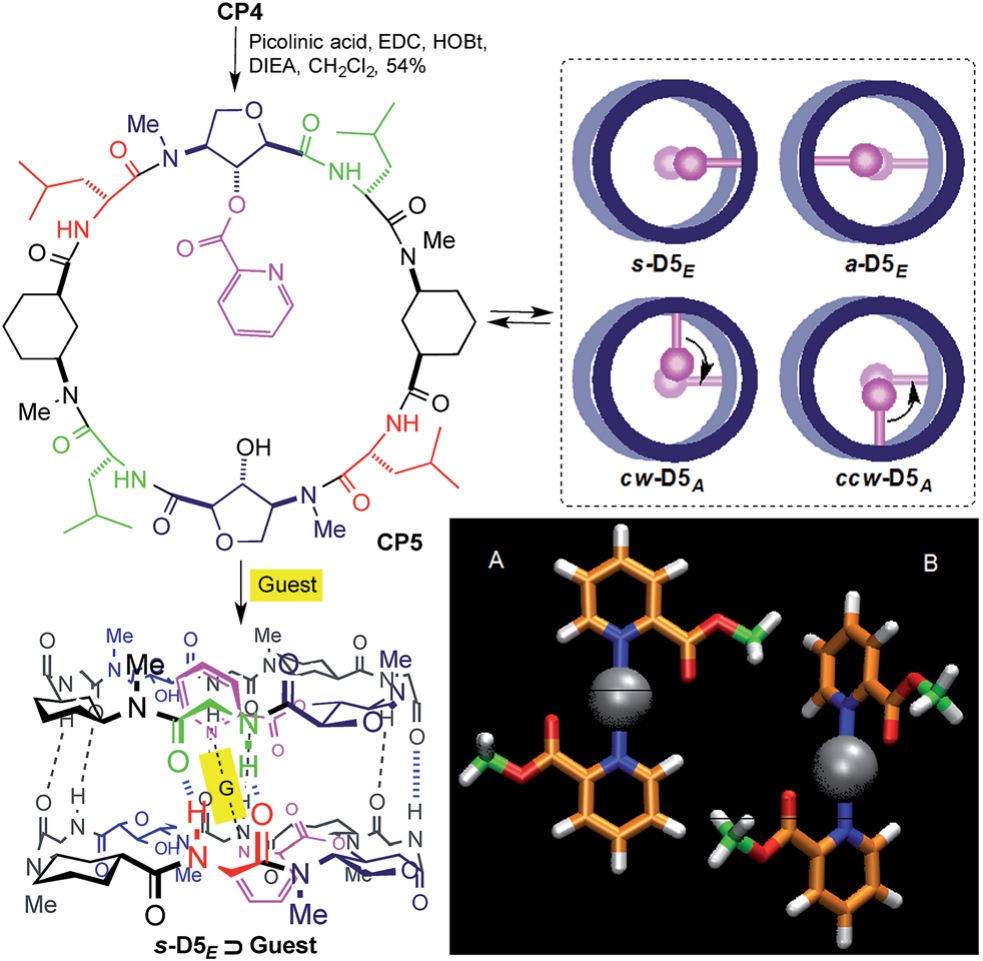
Structure of **CP5** and its guest encapsulation model (**s-D5_E_ ⊃ G**). Top view models of the four resulting dimers are presented in the inset.^14^ Bottom-right corner: DFT models of the two most stable silver–picolinic ester complexes.

In an effort to overcome this problem, a new peptide (**CP4**) was designed in which the amide protons of the Ahf residues were removed by *N*-methylation. To achieve this, compound **2** (Scheme 1) was prepared using Fukuyama sulfonamide alkylation (see Scheme 2SI, ESI†).^11^ Treatment of **2** with thiophenol to remove the nosyl group followed by coupling with Boc-d-Leu-OH gave dipeptide **4**, which was used as the starting material in the synthesis of **CP4** (Scheme 3SI, ESI†). NMR spectroscopy studies on protected **CP3** showed the key features of a dimer, including the downfield shift and the splitting of the amide proton signals (NHs) (9.08 and 8.79, and 8.08 and 8.03 ppm) as a consequence of the formation of two non-equivalent dimers (Fig. 2SI, ESI†) in around a 2 : 1 ratio. Also, the Ahf C2 and C3 signals appeared as a doublet and a doublet of doublets, respectively, as a consequence of the scalar coupling of each proton with their vicinal protons, thus confirming the pseudo-equatorial disposition of the amino, carbonyl and hydroxyl groups. The C2 equatorial proton signals of both Ach moieties of the alternating form (**D3_A_**) were up-field shifted (–1.20 ppm) due to the proximity of the benzyl groups that fill the dimer cavity.^12^ This situation was also confirmed by the nOe cross peak between the NHs and the benzylic hydrogens (Fig. 3SI, ESI†). The nOe cross peak between Hα (Ach) and Hγ (Ahf), in the minor form, implies that this corresponds to the alternating dimer (**D3_A_**). Meanwhile, the nOe cross peaks between the N–H of the non-equivalent Leu, together with the interspace interaction between these protons and Hγ of the pairing CP, confirms that the major ensemble is **D3_E_**. The concentration and temperature independence of the chemical shifts of the NHs implies an association constant (*K*_a_) value greater than 10^5^ M^–1^.^6^ X-ray diffractometry of single crystals obtained from a chloroform/hexanes solution (Scheme 1) provided conclusive proof of dimer formation with **CP3**. In the crystal structure the predominant form is the alternating dimer in which the benzyl groups are pointing towards the cavity, as observed in the solution experiments. The crystallization of the minor component of the dimers mixture (**D3_A_**) must derive from the incorporation of the benzyl groups into the dimer cavity that provides a more compact, symmetric and less flexible structure.

Hydrogenolysis of **CP3** provided **CP4**. This CP presented different NMR behavior. In deuterochloroform solution the signals were broad, especially those corresponding to the amide protons. This behavior results from a folding or aggregation phenomenon for which the inter-exchange rate is similar to the ^1^H NMR time scale. NMR experiments were carried out at lower temperatures in an effort to slow down the process. It was necessary to reduce the temperature to 253 K to observe clearly the amide protons as sharp doublets, although at 0 °C (273 K) two different sets of signals were observed (Fig. 4SI, ESI†). At 253 K two dimers in an almost 1 : 1 ratio were present, as inferred from the number and integrations of the NH signals (8.69, 8.46, 7.88 and 7.73 ppm).

Although dimer formation must be a process that is driven by hydrogen-bonding interactions, the use of a less polar solvent did not favor dimer formation. In fact, the ^1^H NMR spectra of chloroform/toluene (1 : 3) or tetrachloromethane (1 : 1) mixtures did not show any preference for dimer formation. It was necessary to cool the solutions down to lower temperatures to see the key features of the dimeric form clearly. On the other hand, the addition of small amounts of MeOH to a room temperature chloroform solution of **CP4** gave an NMR spectrum with the key features of **D4**. Interestingly, at 253 K the 5% methanol solution of **CP4** showed the preferential formation of one dimer (9 : 1), as evidenced by the two doublets at 8.64 and 8.15 ppm for Leu NHs (Fig. 5SI, ESI†). Two-dimensional NMR experiments (NOESY) showed that this dimer was **D4_A_**. This was identified based on the nOe cross peaks between the Hγ of Ahf (5.31 ppm) with the Hα of Ach (2.78 ppm) and with the NH (8.15 ppm) of one Leu (Fig. 5SI, ESI†). The preferential formation of **D4_A_** in the presence of methanol must be due to the formation of hydrogen bonds between the solvent molecules and the CP hydroxyl groups. In aprotic media the additional hydrogen bond formation between facing hydroxyl groups must contribute to stabilization of the eclipsed dimer. A similar effect was observed on the addition of water, although the solvent immiscibility made it difficult to characterize this system fully. Furthermore, in CDCl_3_ the dimer ratio changed with peptide concentration.^13^ These dimers must therefore encapsulate protic molecules within the cavity and this stabilizes the dimeric form.

Another attractive feature of these ensembles is their simple chemical modification, which allows the cavity properties to be tuned. We envisaged that the incorporation of a pyridine moiety through a simple ester linkage would allow different metals and other guests to be trapped inside the dimeric structure. In order to facilitate metal incorporation only one such group should be attached per CP. Thus, the reaction of **CP4** with one equivalent of picolinic acid gave **CP5** in 54% yield (Scheme 2). This CP self-assembles to form four non-equivalent dimers, although the complexity of the ^1^H NMR spectrum did not allow the relative ratio to be determined.

The silver ion was selected because of its well-known ability to complex with picolinic acid and its linear coordination geometry.^15^ The addition of 0.2 equiv. (per dimer) of AgBF_4_ to the chloroform solution of **CP5** led to changes in the signals of the aromatic and amide protons in the ^1^H NMR spectrum (Fig. 6SI, ESI†). The addition of one equivalent of silver(i) gave a simplified spectrum whose signals correspond mainly to two dimers in a 3 : 1 ratio. In agreement with the NOESY experiments, the major dimer (**s-D5_E_ ⊃ Ag**) is the one in which the two functionalized Ahf units lie on top of each other.^14^ The cross peaks between Hα (2.57 and 2.35 ppm) and Hγ (4.27 and 3.92 ppm) of each Ach (Fig. 6SI, ESI†), which correspond to the intramolecular and to the intradimer cross-relaxation, clearly support this structure. The nOe for H4/H5 of the picolinic moiety with the Hα of Ach^2^ confirms that the silver complex is located inside the cavity (Fig. 1). In addition, the up-field shift of the equatorial proton signal of the other cyclohexyl moiety (Ach^1^), which appears at 0.11 ppm as a consequence of the aromatic ring current,^12^ suggests that the silver complex is slightly tilted towards this residue.

The observed selectivity of the *syn*-eclipsed dimeric form led us to carry out computational studies to correlate with the silver complex geometry. DFT calculations on the bis(methyl picolinate) silver(i) complex showed a preference for the metal to coordinate to both the carbonyl and the pyridine. Two structures were identified as being more stable (Scheme 2A and B): one square planar complex with a *trans* orientation and another structure in which the pyridine moieties are oriented perpendicular to one another. The above structures were not consistent with the observed **s-D5_E_ ⊃ Ag** structure. However, a less stable form (Fig. 8SI, ESI†), in which the Ag is coordinated to the oxygen of Ahf rather than to the carbonyl, would bring the methyl groups to the 5.5 Å distance that correlates quite well with the distance of the C2 of modified Ahf in **s-D5_E_ ⊃ Ag**. Optimization of all four possible resulting dimers at the same level of theory led to the preferred *syn*-eclipsed conformer (Fig. 1).^16^ The energies of the *anti*-eclipsed, clockwise and counter-clockwise alternating dimers are 3.3, 4.6 and 4.7 kcal mol^–1^, respectively, *i.e.*, less stable than the *syn*-eclipsed form (Fig. 9SI, ESI†). The most stable *syn*-eclipsed dimer has a non-symmetric structure with the silver complex slightly tilted towards the Ach,^1^ as suggested by nOe experiments. The silver is coordinated to the N atoms from both pyridines (Ag–N distance of 2.3 Å) and the carbonyl groups of the linker (Ag–O distance of 2.6 Å), leading to a non-linear N–Ag–N angle (157.9°). All eight H-bonds of the dimer are maintained with values in the range 1.9–2.1 Å (the distance C=O–HN).

The basicity of pyridine also allowed a study of the encapsulation of dicarboxylic acids, such as oxalic acid, whose p*K*_a_ values are compatible with the picolinic ester basicity. The addition of 0.2 equiv. of oxalic acid immediately led to changes in the proton NMR spectra (Fig. 7SI, ESI†). After the addition of one equivalent of the acid the spectra showed the formation of one main dimer, as in the case of silver coordination, although in this case there were also additional signals that correspond to two other dimers. Two-dimensional NMR experiments confirmed the formation of the dimer **s-D5_E_ ⊃ (CO_2_H)_2_** (Fig. 7SI, ESI†). The pyridine rings are also tilted towards one of the proximal cyclohexane rings and this leads to an upfield shift in the signal for the equatorial proton at C2,^12^ thus confirming oxalate encapsulation.

## Conclusions

In summary, it has been shown that alternating α,γ-CPs that contain γ-amino acids functionalized with hydroxyl groups at C2 assemble forming dimeric structures with a functionalized polar cavity. This group can be transformed into different groups and this modifies the cavity properties of the resulting dimer. A variety of polar molecules (methanol, water and metal ions) can be incorporated into the cavity, thus modulating the properties of the ensemble. These dimers are models of large aggregates such as peptide nanotubes. We envisage that the use of this amino acid in nanotube-forming peptides would provide nanotubes with tunable properties and that these would have potential uses in separation technologies, catalysis and molecular transport.
