# Supplementary material for: Self-assembling α,γ-cyclic peptides that generate cavities with tunable properties
Source: Chem Sci. 2015 Sep 14;7(1):183–7. doi: 10.1039/c5sc03187g (PMC5515055; doi:10.1039/c5sc03187g)
Supplement: Supplementary file 1 [file SC-007-C5SC03187G-s001.pdf]

Electronic Supplementary Material (ESI) for Chemical Science.
This journal is © The Royal Society of Chemistry 2015

Self-assembling α,γ-cyclic peptides that generate cavities with tunable properties.

Nuria Rodríguez-Vázquez, Rebeca García-Fandiño, Manuel Amorín,* Juan R. Granja*

Singular Research Center in Chemical Biology and Molecular Materials, (CIQUS), Organic Chemistry Department, University of Santiago de Compostela (USC), 15782 Santiago de Compostela, Spain.

juanr.granja@usc.es and manuel.amorin@usc.es

SUPPORTING INFORMATION

Index:

Scheme 1SI (Synthesis of **CP1** and **CP2**). 3

Scheme 2SI (Synthesis of the *N*-methylated Ahf). 4

Scheme 3SI (Synthesis of **CP3**, **CP4** and **CP5**). 5

Figure 1SI (^{1}H NMR spectrum of **CP2**). 6

Figure 2SI (^{1}H NMR spectrum and selected parts of NOESY spectrum of **CP3**). 7

Figure 3SI (Selected parts of NOESY spectrum of **CP3**). 8

Figure 4SI (Temperature and methanol addition experiments of **CP4**). 9

Figure 5SI (^{1}H NMR spectra of **CP4** at 253 K, in $CDCl_3$ and in 5% CD_3OH in $CDCl_3$). 10

Figure 6SI (^{1}H NMR studies of the formation of dimer ***s*-D5$_E$⊃Ag**). 11

Figure 7SI (^{1}H NMR studies of the formation of the dimer ***s*-D5$_E$⊃(CO$_2$H)$_2$**). 12

Figure 8SI (DFT Optimized structures of bis(methyl picolinate) silver(I) complexes). 13

Figure 9SI (DFT Optimized structures of the dimers). 14

Materials and Methods 15

NMR and FTIR Spectra 27

Computational methods 54

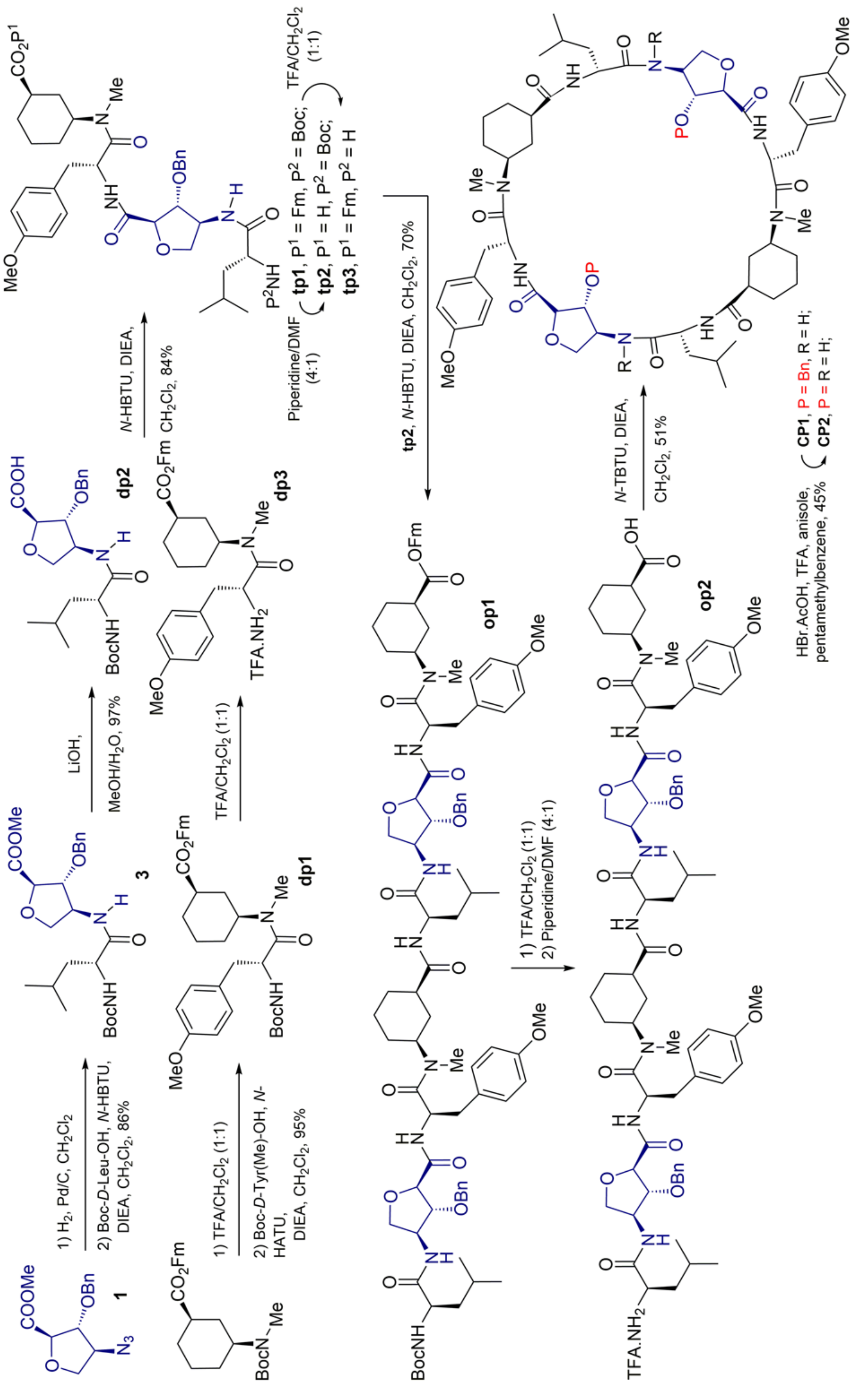

Scheme 1SI: Synthetic strategy for the preparation of cyclic peptides **CP1** and **CP2** by solution phase method.

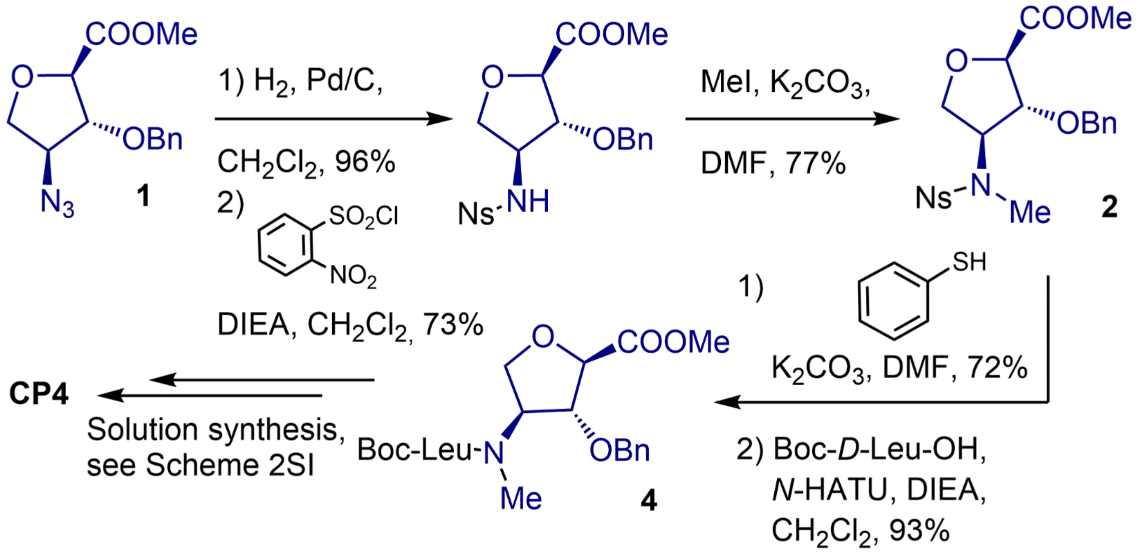

Scheme 2SI: Synthesis of the *N*-methylated Ahf using Fukuyama´s method for the preparation of the fully protected amino acid **2**. Removal of Nosyl group and coupling with Boc-*D*-Leu-OH provide the dipeptide **4**, the basic component used in the synthesis of **CP4**.

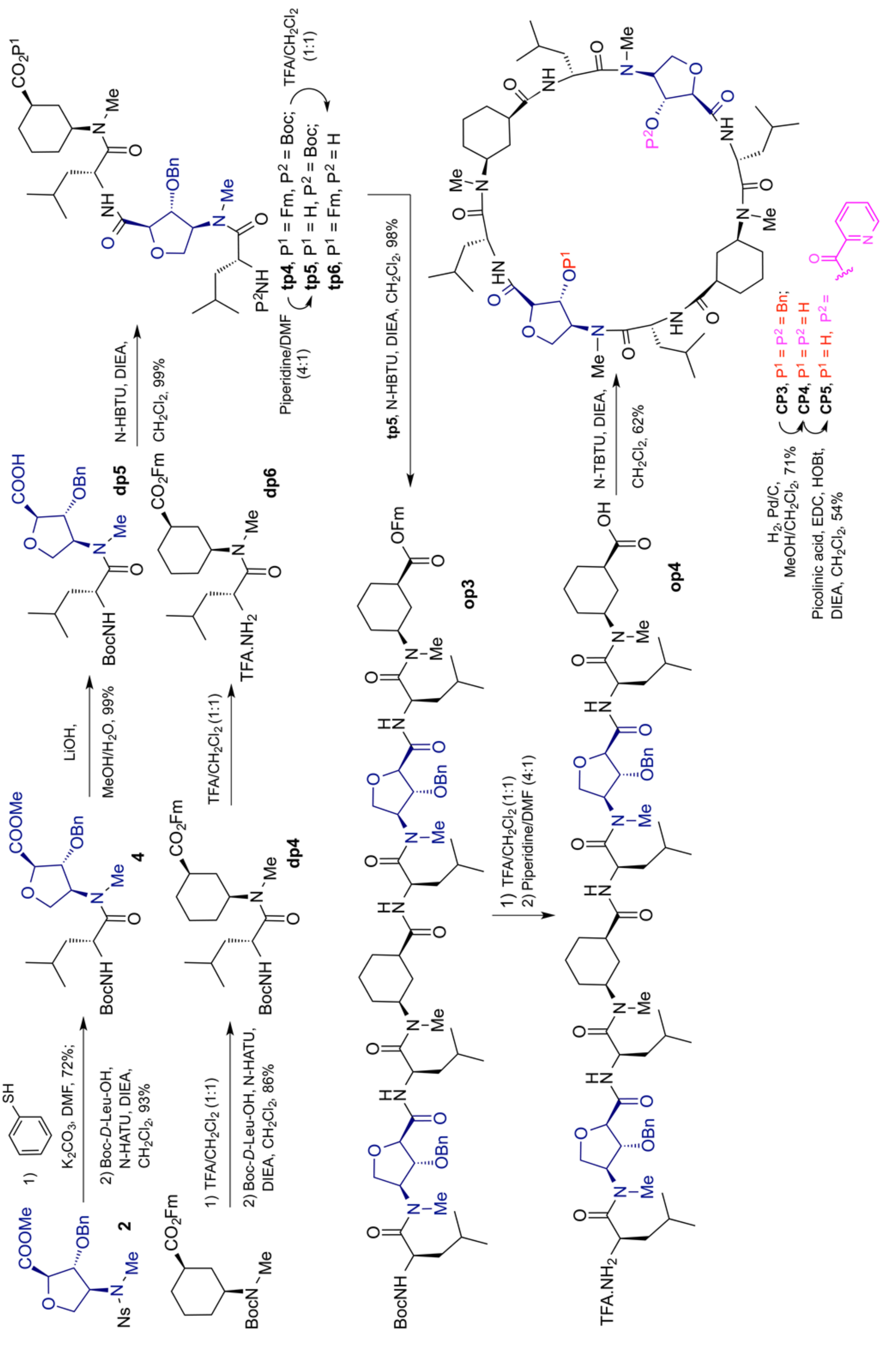

Scheme 3SI: Synthetic strategy for the preparation of cyclic peptides **CP3**, **CP4** and **CP5** by solution phase method.

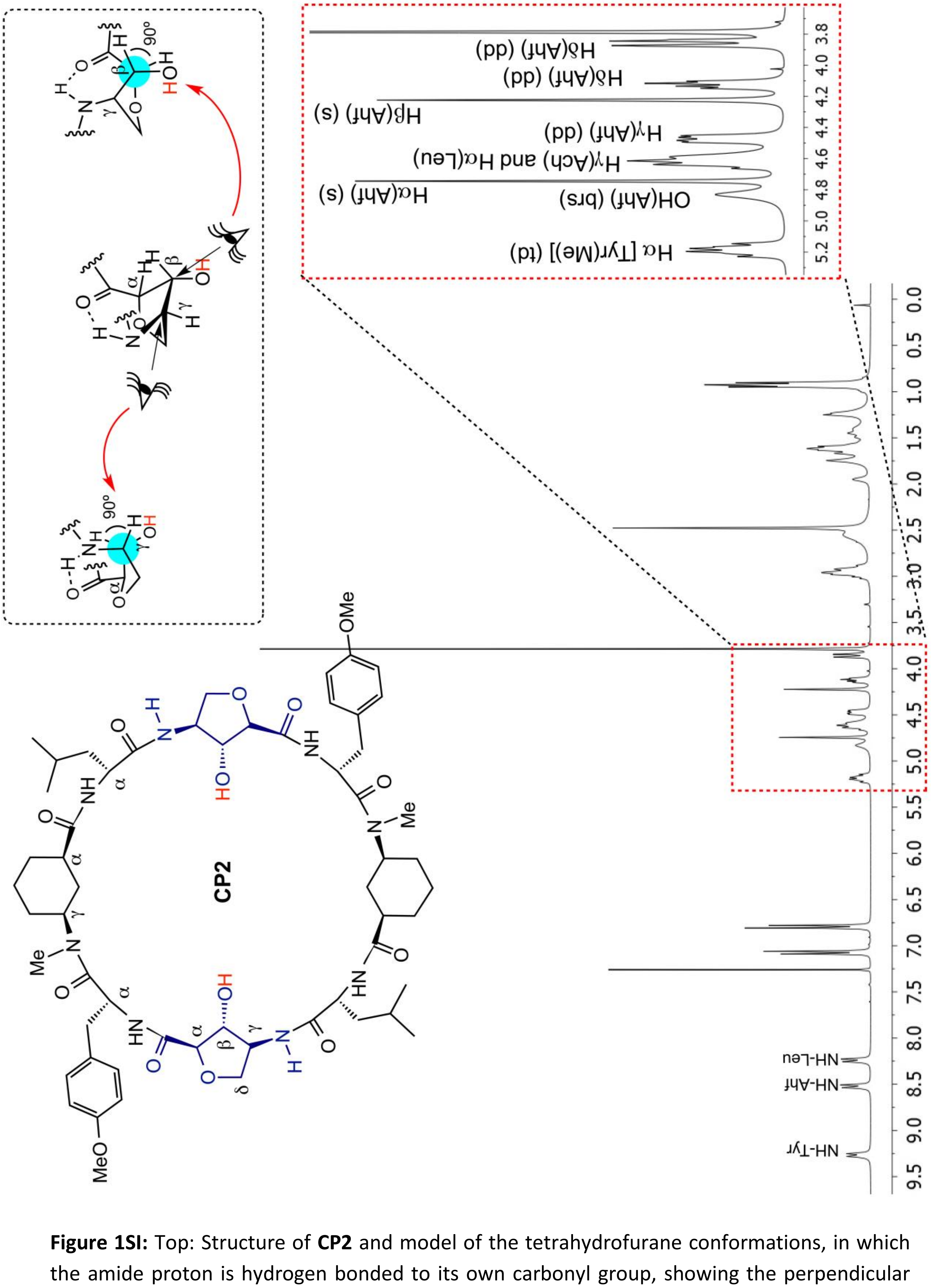

Figure 1SI: Top: Structure of **CP2** and model of the tetrahydrofurane conformations, in which the amide proton is hydrogen bonded to its own carbonyl group, showing the perpendicular orientation between the Hα and the Hβ and between the Hγ and the Hβ. Bottom: ^{1}H NMR spectrum of **CP2** (20 mM, $CDCl_3$, 298 K); in the inset, an extension of the spectral region between 5.4 and 3.6 ppm should be shown, where the vicinal protons α-Ahf and β-Ahf appear as singlets, which suggests their perpendicular orientation.

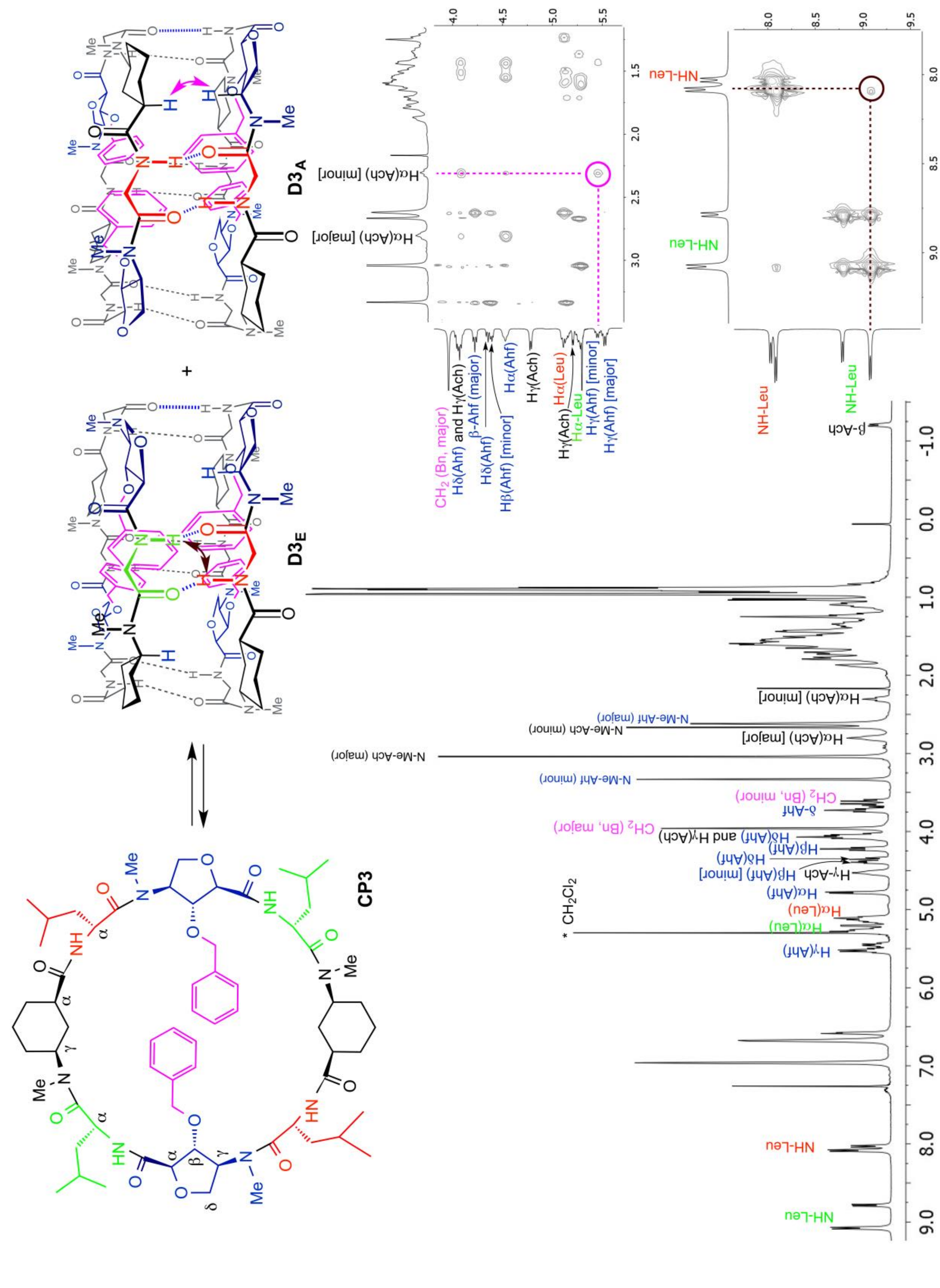

Figure 2SI: Top: Structure of **CP3** and model of the two possible dimers: eclipsed (**D3$_E$**) and alternated (**D3$_A$**). Bottom left: ^{1}H NMR spectrum of **CP3** (16 mM, $CDCl_3$, 298 K), showing the formation of the two non-equivalent dimers in a 2:1 ratio (**D3$_E$:D3$_A$**). Bottom right: Selected parts of NOESY spectrum showing the nOe cross peaks between the α-Ach proton and the γ-Ahf (pink) in the minor form (**D3$_A$**) and between the amide protons (N-H) of the two non-equivalent leucines (brown) of the major ensemble (**D3$_E$**).

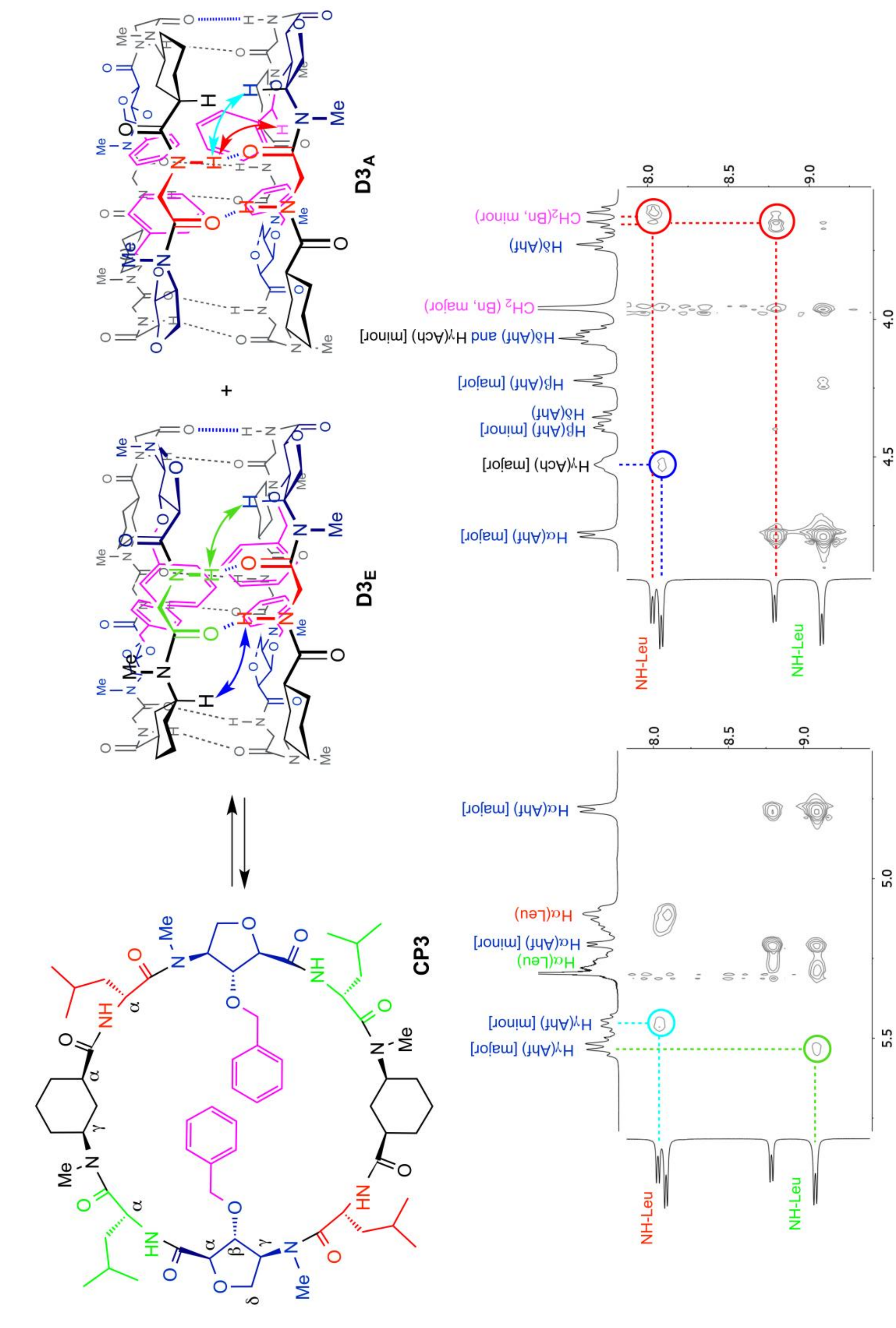

Figure 3SI: Top: Structure of **CP3** and model of the two possible dimers: eclipsed (**D3$_E$**) and alternated (**D3$_A$**). Bottom left: Selected part of NOESY spectrum showing the nOe cross peaks between the γ-Ahf and the Leu N-H (light blue) in the minor form (**D3$_A$**), and the between the γ-Ahf and Leu N-H (green) in the major ensemble (**D3$_E$**). Bottom right: Selected part of NOESY spectrum showing the nOe cross peaks (red) between the amide protons and the benzylic protons in the minor form (**D3$_A$**), and between the γ-Ach and Leu N-H (dark blue) of the major ensemble (**D3$_E$**).

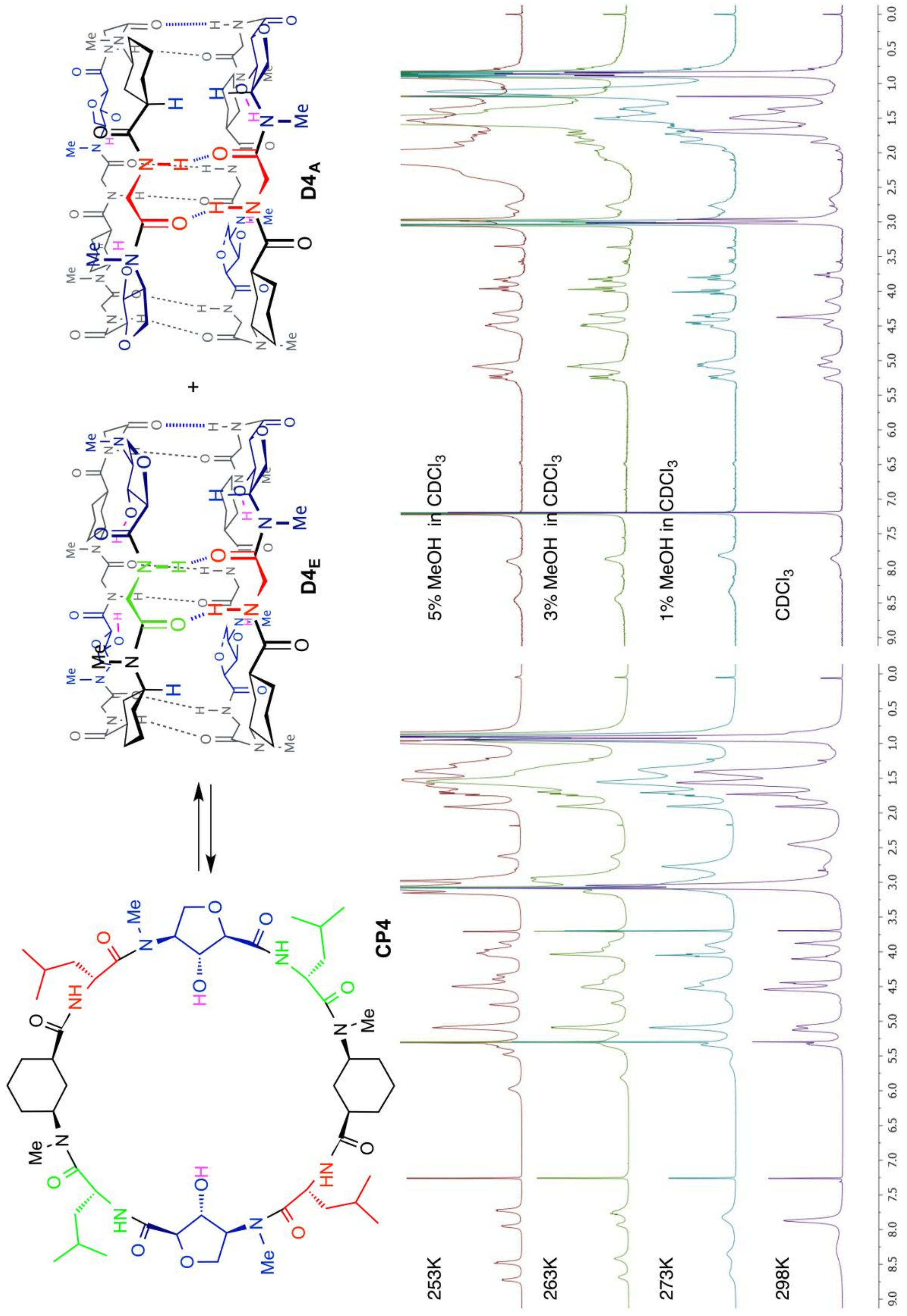

Figure 4SI: Top: Structure of **CP4** and model of the two possible dimers: eclipsed (**D4$_E$**) and alternated (**D4$_A$**). Bottom left: ^{1}H NMR spectra of **CP4** (5 mM, $CDCl_3$) at different temperatures. Bottom right: ^{1}H NMR experiment of methanol additions (1-5%) to a 5 mM solution of **CP4** in $CDCl_3$.

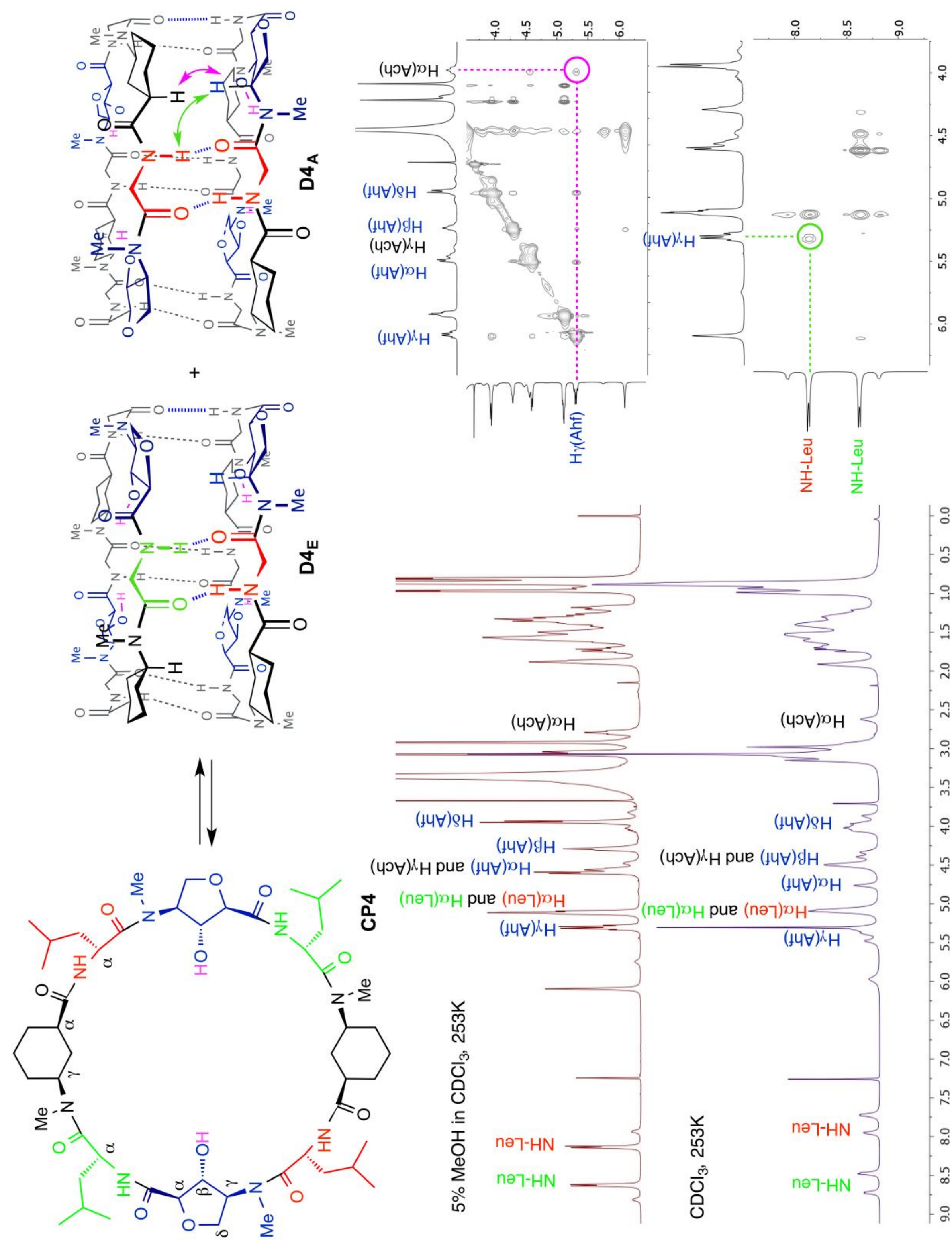

Figure 5SI: Top: Structure of **CP4** and model of the two possible dimers: eclipsed (**D4$_E$**) and alternated (**D4$_A$**). Bottom left: ^{1}H NMR spectra of **CP4** (32 mM in $CDCl_3$) at 253 K, in $CDCl_3$ and in 5% CD_3OH in $CDCl_3$, showing that at this temperature the addition of methanol induces the formation of mainly one dimer (**D4$_A$**). Bottom right: Selected part of NOESY spectrum showing the nOe cross peaks between the α-Ach proton and the γ-Ahf (pink) and between the N-H and the γ-Ahf (green) in the alternated form (**D4$_A$**).

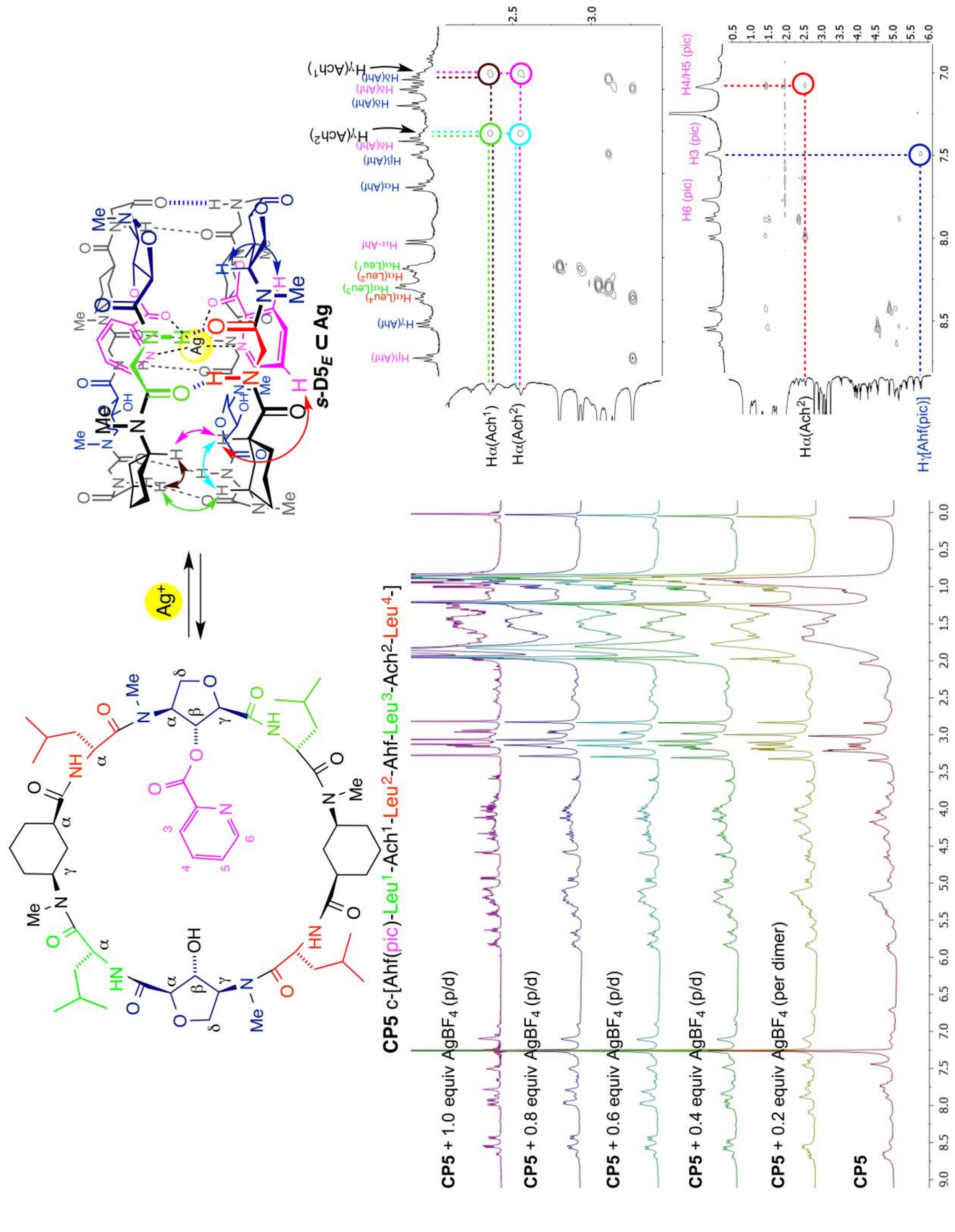

Figure 6SI: Top: Structure of **CP5** and model of the silver-encapsulated dimer (***s*-D5$_E$⊃Ag**). Bottom left: ^{1}H NMR studies of the formation of dimer ***s*-D5$_E$⊃Ag** by the addition of different equivalents of $AgBF_4$ (0.2-1.0 equiv per dimer) to a $CDCl_3$ solution of **CP5** (5 mM, $CDCl_3$, 298 K). Bottom right: Selected parts of NOESY spectrum showing the nOe cross peaks between the Hγ of each Ach and both Hα of Ach that support the formation of the *syn*-eclipsed form (***s*-D5$_E$⊃Ag**). The nOe cross peaks of picolinic moiety protons are also shown, which suggest the incorporation of the silver complex in the dimer cavity.

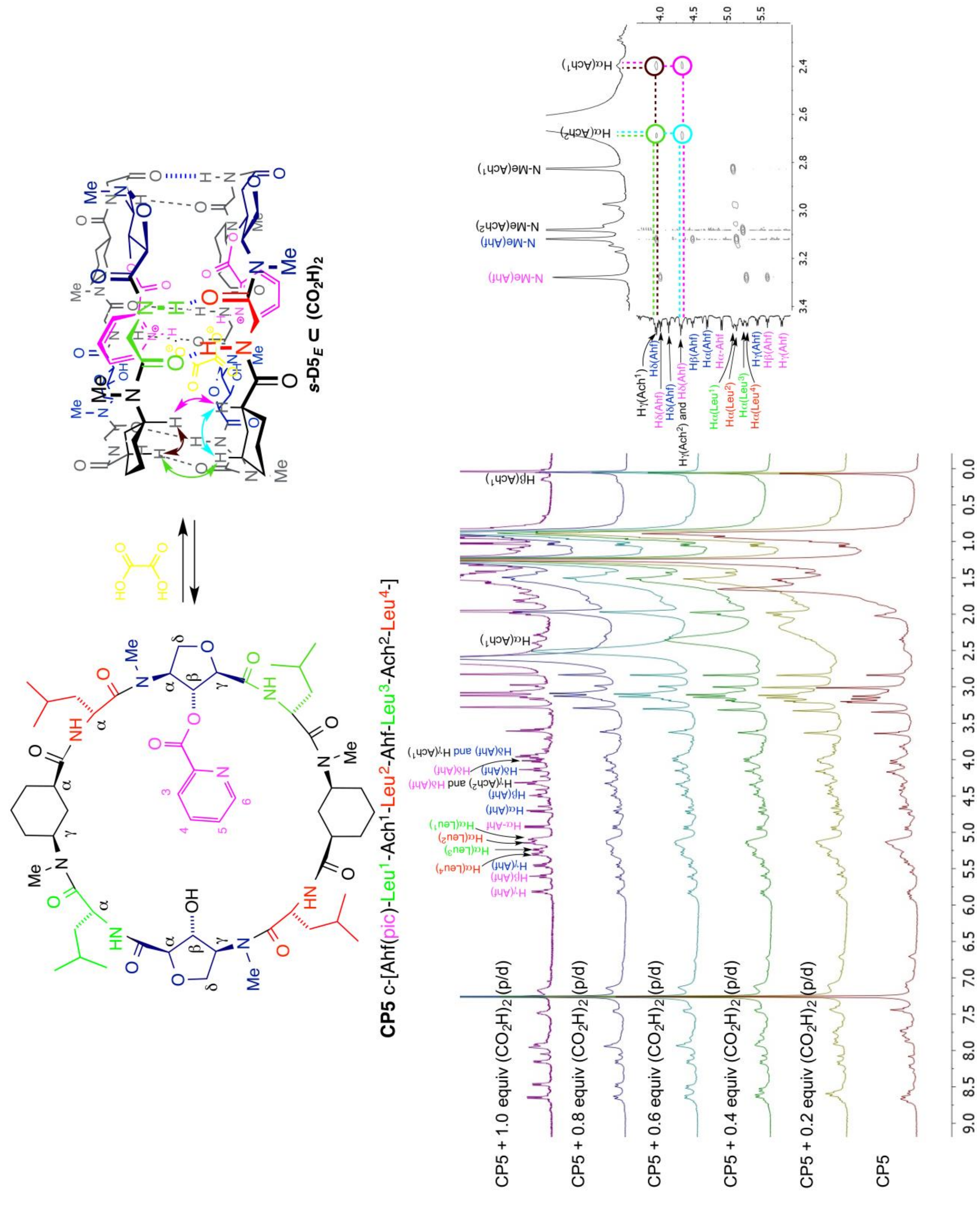

Figure 7SI: Top: Structure of **CP5** and model of the oxalic acid encapsulated in the corresponding dimer (***s*-D5$_E$⊃(CO$_2$H)$_2$**). Bottom left: ^{1}H NMR studies of the formation of the dimer ***s*-D5$_E$⊃(CO$_2$H)$_2$** by the addition of different equivalents of oxalic acid (0.2-1.0 equiv per dimer) to a CDCl$_3$ solution of **CP5** (5 mM, CDCl$_3$, 298 K). Bottom right: Selected parts of NOESY spectrum showing the nOe cross peaks between the Hγ of each Ach and both Hα of the same residue that support the formation of the *syn*-eclipsed form (***s*-D5$_E$⊃(CO$_2$H)$_2$**).

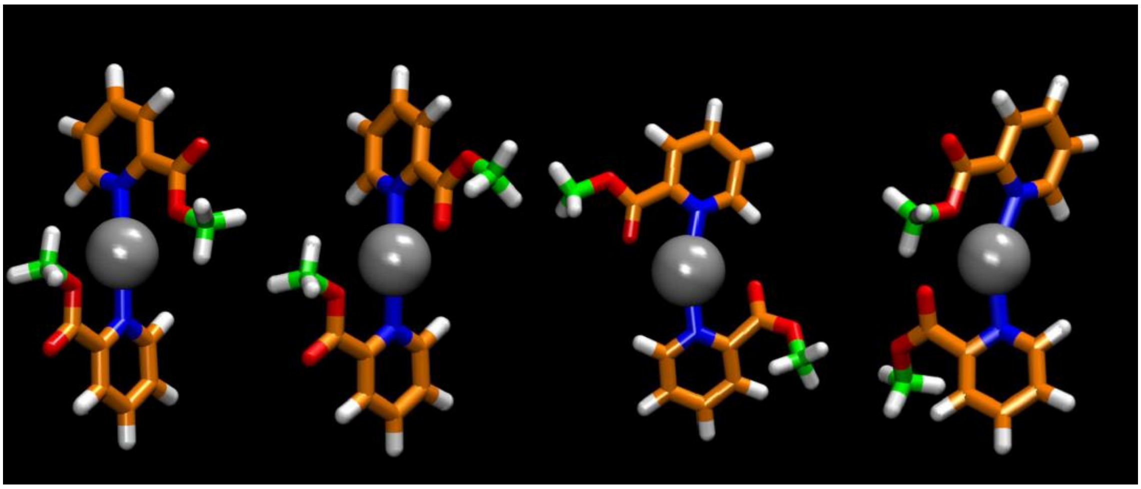

Figure 8SI: DFT Optimized structures of bis(methyl picolinate) silver(I) complexes with higher (less stable) energies compared to those presented in the main part of the manuscript. The energies of these conformations are about 11, 5, 13 and 18 kcal/mol (from left to right) less stable than those corresponding to the conformers presented there. The silver ion is in gray whereas the two picolinates are highlighted in orange.

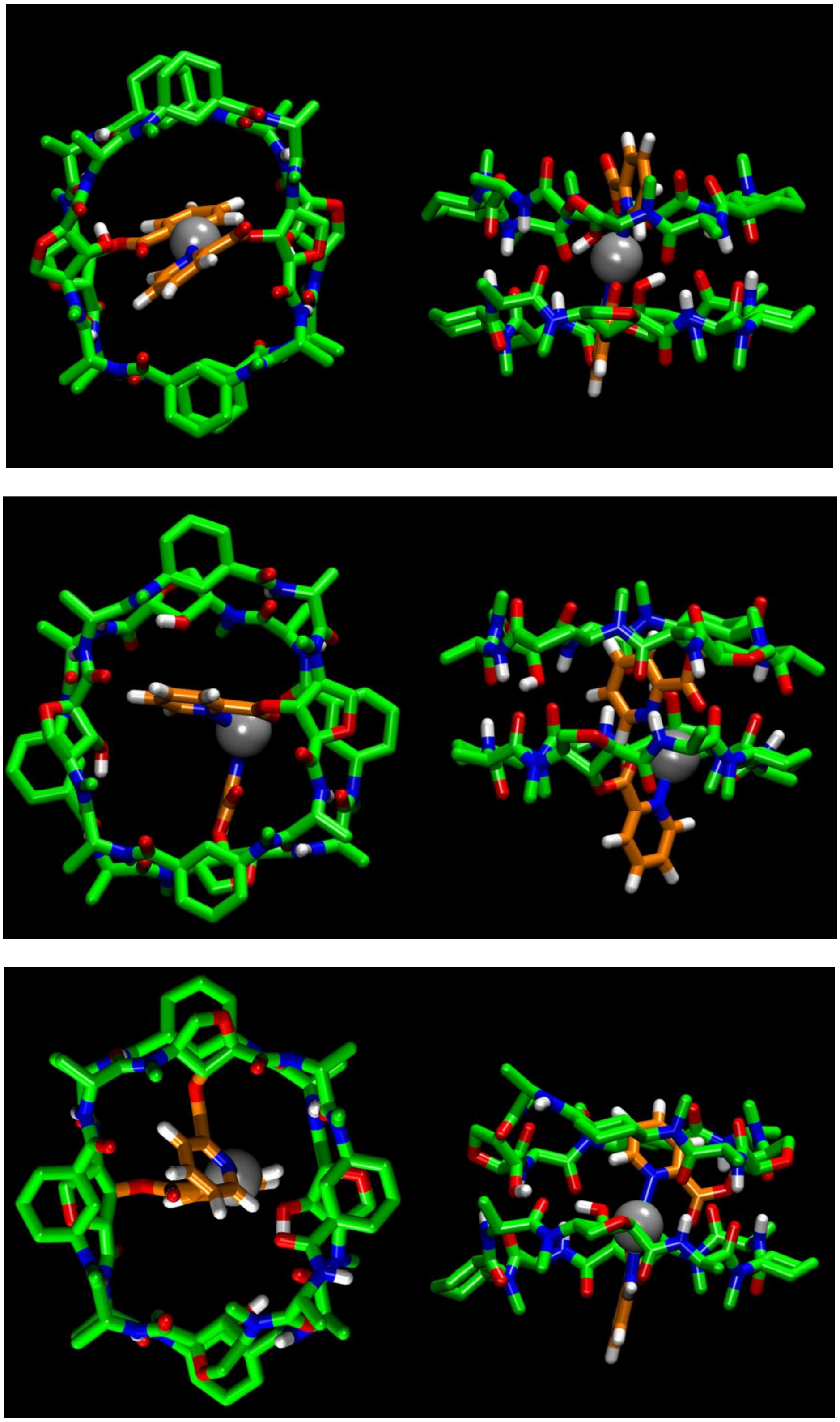

Figure 9SI: Top and lateral views of the DFT optimized structures for the *anti*-eclipsed, clockwise and counter-clockwise alternating dimers, respectively. All the hydrogens, except those from the backbone and those from the pyridines, have been removed for clarity. The side chains were changed to methyl groups to reduce the number of possible conformers.

Materials and Methods:

General:

1-[bis(dimethylamino)methylene]-1*H*-1,2,3-triazolo-[4,5-*b*]pyridinium hexafluorophosphate 3-oxide (*N*-HATU), 1-[bis(dimethylamino)methylene]-1*H*-benzotriazolium hexafluorophosphate 3-oxide (*N*-HBTU), 1-[bis(dimethylamino)methylene]-1*H*-benzotriazolium tetrafluoroborate 3-oxide (*N*-TBTU),[1] *N*-(3-dimethylaminopropyl)-*N*′-ethylcarbodiimide hydrochloride (EDC), 1-hydroxybenzotriazole (HOBt), 4-dimethylaminopyridine (DMAP) and α-aminoacids were purchased from Iris Biotech, Aldrich or from Globel Sales Manager, GL Biochem (Shanghai) Ltd, China. All reagents and solvents were used as received unless otherwise noted. CH_2Cl_2 and DIEA to be used as reaction solvents were distilled from CaH_2 over argon immediately prior to use. Analytical thin-layer chromatography was performed on E. Merck silica gel 60 F_{254} plates. Compounds, which were not UV active, were visualized by dipping the plates in a nynhidrin solution and heating. Silica gel flash chromatography was performed using E. Merck silica gel (type 60SDS, 230-400 mesh). Solvent mixtures for chromatography are reported as v/v ratios. HPLC purification was carried out on a HITACHI D-7000 using a Phenomenex Luna 5µ Silica 100 Angstroms column with CH_2Cl_2/MeOH gradients between 96:4 and 87:13. **^{1}H NMR** spectra were recorded on Varian Inova 500 MHz, Varian Mercury 300 MHz or Bruker DPX 250 MHz spectrometers. Chemical shifts (δ) were reported in parts per million (ppm) relative to tetramethylsilane (δ = 0.00 ppm) or by the deuterated solvent. ^{1}H NMR splitting patterns are designated as singlet (s), doublet (d), triplet (t), or quartet (q). All first-order splitting patterns were assigned on the basis of the appearance of the multiplet. Splitting patterns that could not be easily interpreted are designated as multiplet (m) or broad (br). **^{13}C NMR** spectra were recorded on Varian Mercury 300 MHz spectrometer. Carbon resonances were assigned using distortionless enhancement by polarization transfer (DEPT) spectra obtained with phase angles of 135. **^{1}H NMR Assignments of Cyclic Peptides (CPs).** The signals of the ^{1}H NMR spectra of the peptides were identified from the corresponding double-quantum-filled 2D COSY, TOCSY and NOESY and/or ROESY spectra acquired at concentration and temperature indicated (Mixing times for NOESY and/or ROESY –between 250 and 1000 ms- were not optimized). Electrospray (ESI) mass spectra were recorded on a Bruker BIOTOF II mass spectrometer. FTIR measurements were made on a JASCO FT/IR-400 spectrophotometer placing the sample on a CaF_2 pellet.

[1] L. A. Carpino, et all., *Angew. Chem. Int. Ed.* **2002**, *41*, 41–445.

Peptide synthesis:

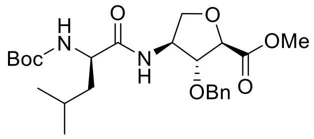

Boc-*D*-Leu-*L*-Ahf(Bn)-OMe (3): A solution of N_3-*L*-Ahf(Bn)-OMe[2] (271 mg, 0.97 mmol) and Pd/C (208 mg, 10% in wt) in CH_2Cl_2 (10 mL) was stirred at rt for 2 h under hydrogen atmosphere (balloon pressure). The resulting mixture was filtered through a Celite pad, the residue was washed with CH_2Cl_2, and the combined filtrates and washes were concentrated under reduced pressure. The crude was used without further purification. A solution of the resulting NH_2-*L*-Ahf(Bn)-OMe in CH_2Cl_2 (15 mL) was successively treated with DIEA (0.71 mL, 3.9 mmol), Boc-*D*-Leu-OH (225 mg, 1.0 mmol), and *N*-HBTU (405.0 mg, 1.1 mmol), and the mixture was stirred at rt under Argon for 90 min. The solution was washed with aqueous HCl (5%, 3 x 20 mL) and aqueous saturated $NaHCO_3$ (2 x 20 mL). The organic layer was dried with anhydrous Na_2SO_4, filtered and concentrated under reduced pressure. The resulting crude was purified by flash chromatography (0-2% MeOH/CH_2Cl_2) to give 387 mg of dipeptide **3**. [Yellow foam, 86%, R_f = 0.58 (5% MeOH/CH_2Cl_2)]. **^{1}H NMR** ($CDCl_3$, 250 MHz, δ): 7.51-7.24 (m, 6H), 6.63 (d, *J* = 8.1 Hz, 1H), 4.77 (AB, *J* = 11.8 Hz, 1H), 4.83-4.49 (m, 2H), 4.66 (AB, *J* = 11.8 Hz, 1H), 4.47 (d, *J* = 1.6 Hz, 1H), 4.24-3.91 (m, 3H), 3.77 (s, 3H), 1.86-1.51 (m, 3H), 1.43 (s, 9H), 0.93 (d, *J* = 5.7 Hz, 6H). **^{13}C NMR** ($CDCl_3$, 75.4 MHz, δ): 172.8 (CO), 171.7 (CO), 155.4 (CO), 137.3 (C), 128.3 (CH), 127.8 (CH), 127.7 (CH), 86.6 (CH), 82.1 (CH), 79.8 (C), 72.5 (CH_2), 71.8 (CH_2), 54.7 (CH_3), 53.4 (CH), 52.4 (CH), 41.4 (CH_2), 28.2 (CH_3), 24.7 (CH_3), 24.6 (CH_3), 23.0 (CH). **MS (ESI)** [m/z(%)]: 487 ([MNa]$^+$), 465 ([MH]$^+$). **HRMS (ESI) calculated** for $C_{24}H_{37}N_2O_7$: 465.2595, **found**: 465.2595.

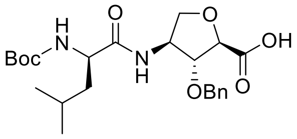

Boc-*D*-Leu-*L*-Ahf(Bn)-OH (dp2): A solution of dipeptide **3** (387 mg, 0.83 mmol) in a mixture of MeOH and water (3:1, 20 mL) was treated with LiOH (100 mg, 4.2 mmol). The solution was stirred at rt for 5 h and then the solvent was removed under reduced pressure. The resulting residue was diluted with water (10 mL) and washed with Et_2O (10 mL), and the resulting aqueous solution was acidified to pH 3 with aqueous HCl (5%). The acidic solution was extracted with CH_2Cl_2 (4 x 10 mL), the combined organic extracts were dried with anhydrous Na_2SO_4, filtered and concentrated under reduced pressure to give 365 mg of dipeptide **dp2**. [Yellow foam, 97%, R_f = 0.20 (5% MeOH/CH_2Cl_2)]. **^{1}H RMN** ($CDCl_3$, 300 MHz, δ): 10.1 (br, 1H), 7.56 (d, *J* = 6.8 Hz, 1H), 7.49-7.14 (m, 5H), 6.42 (d, *J* = 7.0 Hz, 1H), 5.35 (d, *J* = 8.4 Hz, 1H), 4.78 (AB, *J* = 11.9 Hz, 1H), 4.69 (AB, *J* = 11.6 Hz, 1H), 4.57-3.91 (m, 5H), 1.74-1.39 (m, 3H), 1.41 and 1.37 (s, 9H), 0.91 (d, *J* = 6.3 Hz,

[2] N. Rodríguez-Vázquez, S. Salzinger, L. F. Silva, M. Amorín, J. R. Granja, *Eur. J. Org. Chem.* **2013**, *17*, 3477–3493.

3H), 0.89 (d, *J* = 6.3 Hz, 3H). **^{13}C RMN** ($CDCl_3$, 75.4 MHz, δ): 173.7 (CO), 172.6 (CO), 156.5 (CO), 137.4 (C), 128.4 (CH), 127.8 (CH), 127.6 (CH), 87.1 (CH), 82.0 (CH), 81.8 (C), 72.6 (CH_2), 71.8 (CH_2), 54.9 (CH), 53.5 (CH), 41.2 (CH_2), 28.2 (CH_3), 24.7 (CH_3), 23.0 (CH_3), 21.9 (CH). **MS (ESI)** [m/z(%)]: 473 ($[MNa]^+$), 452 ($[MH]^+$). **HRMS (ESI) calculated** for $C_{23}H_{35}N_2O_7$: 452.2425, **found**: 452.2439.

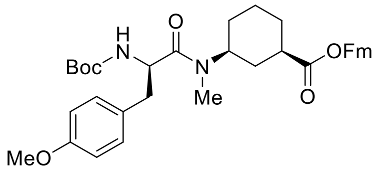

Boc-*D*-Tyr(Me)-*L*-^{Me}*N*-Ach-OFm (dp1): A solution of Boc-*L*-^{Me}*N*-Ach-OFm (811 mg, 1.86 mmol) in mixture of TFA and CH_2Cl_2 (1:1, 10 mL) was stirred at rt for 10 min. After removal of the solvent under vacuum, the residue was dried under high vacuum for 3 h. The resulting TFA salt was dissolved under Argon in CH_2Cl_2 (10 mL), then DIEA (1.3 mL, 7.5 mmol), Boc-*D*-Tyr(Me)-OH (550 mg, 1.9 mmol) and *N*-HATU (778 mg, 2.0 mmol) were successively added. After 1 h stirring at rt, the solution was washed with aqueous HCl (5%, 2 x 10 mL) and aqueous saturated $NaHCO_3$ (2 x 10 mL). The organic layer was dried with anhydrous Na_2SO_4, filtered and concentrated under reduced pressure, providing a yellow oil that was purified by flash chromatography (0-2% MeOH/CH_2Cl_2) to give 1.0 g of dipeptide **dp1**. [White foam, 95%, R_f = 0.75 (5% MeOH/CH_2Cl_2)]. **^{1}H NMR** ($CDCl_3$, 300 MHz, δ): 7.71 (m, 2H), 7.61 (d, , *J* = 7.4 Hz, 1H), 7.54 (m, 2H), 7.43-7.20 (m, 4H), 7.08 (m, 2H), 6.76 (m, 2H), 5.51 (m, 1H), 4.73 (m, 1H), 4.46-4.32 (m, 2H), 4.22-4.04 (m, 1H), 4.14 (AB, *J* = 6.6 Hz, 1H), 3.95 (AB, *J* = 6.6 Hz, 1H), 3.73 and 3.60 (s, 3H), 2.89 (m, 1H), 2.70 and 2.46 (s, 3H), 1.96-0.71 (m, 8H), 1.42 and 1.40 (s, 9H). **^{13}C NMR** ($CDCl_3$, 75.4 MHz, δ): 174.6 (CO), 171.3 (CO), 158.5 (C), 155.0 (CO), 144.7 (C), 130.4 (CH), 128.4 (C), 127.7 (CH), 127.0 (CH), 124.8 (CH), 119.9 (CH), 113.7 (CH), 79.5 (C), 65.9 (CH_2), 55.1 (CH), 51.5 (CH), 50.3 (CH_3), 46.9 (CH), 42.2 (CH), 39.3 (CH_2), 38.4 (CH_3), 31.3 (CH_2), 29.7 (CH_2), 28.2 (CH_3), 24.2 (CH_2). **MS (ESI)** [m/z(%)]: 635.3 ($[MNa]^+$, 62), 613.3 ($[MH]^+$, 38), 557.3 ($[MH\text{-}^tBu]^+$, 97), 513.3 ($[MH\text{-}Boc]^+$, 100). **HRMS (ESI) calculated** for $C_{37}H_{45}N_2O_6$: 613.3272, **found**: 613.3273.

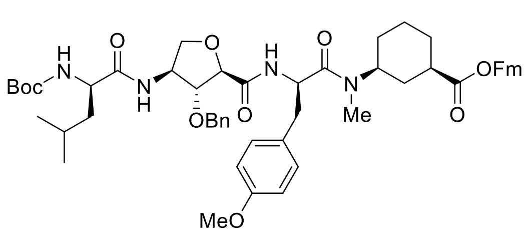

Boc-*D*-Leu-*L*-Ahf(Bn)-*D*-Tyr(Me)-*L*-^{Me}*N*-Ach-OFm (tp1): A solution of dipeptide **dp1** (497.0 mg, 0.81 mmol) in mixture of TFA and CH_2Cl_2 (1:1, 10.0 mL) was stirred at rt for 30 min. After removal of the solvent under vacuum, the residue was dried under high vacuum for 3 h. The resulting TFA salt (**dp3**) was dissolved under Argon in dry CH_2Cl_2 (14 mL), then DIEA (560 µL, 3.2 mmol), dipeptide **dp2** (365 mg, 0.81 mmol), and *N*-HBTU (338 mg, 0.89 mmol) were successively added. After 2 h stirring at rt, the solution was washed with aqueous HCl (5%, 2 x 15 mL) and aqueous saturated $NaHCO_3$ (2 x 15 mL). The organic layer was dried with anhydrous Na_2SO_4, filtered and concentrated under reduced pressure. The resulting

crude was purified by flash chromatography (0-2% MeOH/CH_2Cl_2) to give 645 mg of tetrapeptide **tp1**. [White foam, 84%, R_f = 0.42 (5% MeOH/CH_2Cl_2)]. **MS (ESI)** [m/z(%)]: 967.5 ([MNa]$^+$, 30), 945.5 ([MH]$^+$, 12), 748.4 (100). **HRMS (ESI) calculated** for $C_{55}H_{69}N_4O_{10}$: 945.5008, **found**: 945.5001.

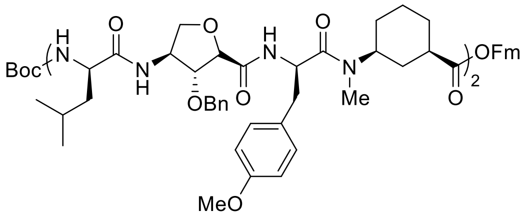

Boc-[*D*-Leu-*L*-Ahf(Bn)-*D*-Tyr(Me)-*L*-^{Me}*N*-Ach]$_2$-OFm (op1): A solution of tetrapeptide **tp1** (305 mg, 0.32 mmol) in a mixture of piperidine and CH_2Cl_2 (1:4, 6 mL) was stirred at rt for 45 min. The solution was washed with aqueous HCl (5%, 4 x 5 mL), dried with anhydrous Na_2SO_4, filtered and concentrated, to give Boc-*D*-Leu-*L*-Ahf(Bn)-*D*-Leu-*L*-^{Me}*N*-Ach-OH (**tp2**), which was used without further purification.

A solution of tetrapeptide **tp1** (305 mg, 0.32 mmol) in mixture of TFA and CH_2Cl_2 (1:1, 8 mL) was stirred at rt for 15 min. After removal of the solvent under vacuum, the residue was dried under high vacuum for 3 h. The resulting TFA salt (**tp3**) was dissolved in dry CH_2Cl_2 (12 mL) under Argon, and successively treated with DIEA (222 µL, 1.3 mmol), the previously prepared **tp2** and *N*-HBTU (134 mg, 0.35 mmol). After 1 h stirring at rt, the solution was washed with aqueous HCl (5%, 2 x 10 mL) and aqueous saturated $NaHCO_3$ (2 x 10 mL). The organic layer was dried with anhydrous Na_2SO_4, filtered and concentrated under reduced pressure. The resulting crude was purified by flash chromatography (0-2% MeOH/CH_2Cl_2) to give 360 mg of octapeptide **op1**. [White foam, 70%, R_f = 0.45 (5% MeOH/CH_2Cl_2)]. **MS (ESI)** [m/z(%)]: 1593.8 ([MH]$^+$, 8), 845.4 ([tetrapeptide-Boc]$^+$, 100), 747.9 ([MH-Boc]$^{2+}$, 14). **HRMS (ESI) calculated** for $C_{91}H_{117}N_8O_{17}$: 1593.8531, **found**: 1593.8586.

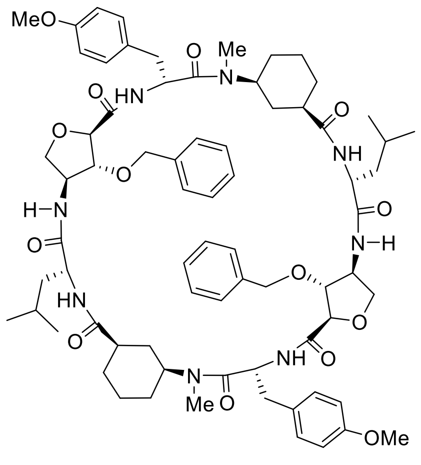

***c*-{[*L*-Ahf(Bn)-*D*-Tyr(Me)-*L*-^{Me}*N*-Ach-*D*-Leu-]$_2$} (CP1):** The linear octapeptide **op1** (150.0 mg, 94.1 µmol) was dissolved in a mixture of piperidine and CH_2Cl_2 (1:4, 5 mL) and stirred at rt for 45 min. The solution was washed with aqueous HCl (5%, 4 x 5 mL), dried with anhydrous Na_2SO_4, filtered and concentrated to give Boc-[*D*-Leu-*L*-Ahf(Bn)-*D*-Tyr(Me)-*L*-^{Me}*N*-Ach]$_2$-OH, which was used without further purification. The resulting C-unprotected octapeptide was dissolved in a mixture of TFA and CH_2Cl_2 (1:1, 10 mL) and stirred at rt for 30 min. After removal of the solvent under reduced pressure, the residue was dried under high vacuum for 3 h and used without further purification. The

resulting unprotected linear peptide (**op2**) was dissolved in dry CH_2Cl_2 (95 mL) and successively treated with DIEA (66 µL, 0.38 mmol) and *N*-TBTU (39.2 mg, 0.10 mmol). After 12 h, the solvent was removed under reduced pressure, and the residue was dissolved in CH_2Cl_2 (15 mL), washed with aqueous HCl (10%, 2 x 10 mL), dried with anhydrous Na_2SO_4, filtered and concentrated to dryness. The crude was purified by flash chromatography (0-5% MeOH/CH_2Cl_2) to give 62.5 mg of the desired cyclicpeptide. [White solid, 51%, R_f = 0.47 (5% MeOH/CH_2Cl_2)]. **^{1}H NMR** (7.5 mM, $CDCl_3$, 300 MHz, δ): 7.29 (m, 12H), 7.09 (d, *J* = 8.6 Hz, 5H), 6.82 (d, *J* = 8.5 Hz, 5H), 6.53 (d, *J* = 8.7 Hz, 1H), 5.59 (d, *J* = 9.3 Hz, 1H), 4.99-3.82 (m, 18H), 3.80 (s, 6H), 3.44 (m, 2H), 2.53 (m, 2H), 2.24 (s, 6H), 2.11-1.08 (m, 26H), 0.90 (d, *J* = 7.5 Hz, 6H), 0.87 (d, *J* = 6.8 Hz, 6H). **^{13}C NMR** ($CDCl_3$, 75.4 MHz, δ): 175.2 (CO), 174.0 (CO), 172.7 (CO), 172.0 (CO), 170.0 (C), 159.2 (C), 137.7 (C), 130.5 (CH), 128.5 (CH), 127.4 (CH), 114.2 (CH), 87.7 (CH), 84.9 (CH), 72.4 (CH_2), 72.1 (CH_2), 55.7 (CH), 55.5 (CH_3), 51.9 (CH), 45.0 (CH), 42.1 (CH_2), 39.6 (CH_2), 32.8 (CH_2), 29.6 (CH_3), 28.5 (CH_2), 25.2 (CH), 24.9 (CH_2), 23.3 (CH_3), 21.4 (CH_3). **FTIR** (298 K, $CHCl_3$): 3297 (amide A), 3006, 2958, 2933, 2869, 1655 (amide I_{II}), 1626 (amide I), 1514 cm^{-1} (amide II). **MS (ESI)** [m/z(%)]: 1319.7 ($[MNa]^+$, 19), 1297.7 ($[MH]^+$, 12), 668.3 ($[MHK]^{2+}$, 100), 660.8 ($[MHNa]^{2+}$, 23). **HRMS (ESI) calculated** for $C_{72}H_{97}N_8O_{14}$: 1297.7119, **found**: 1297.7111.

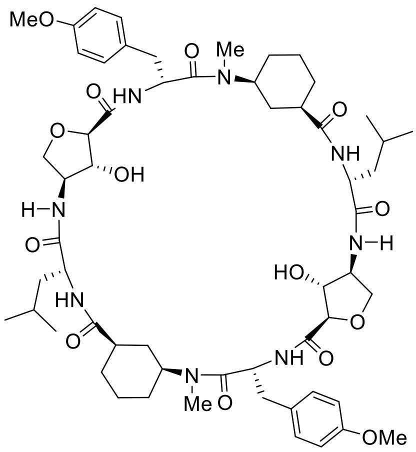

***c*-{[*L*-Ahf-*D*-Tyr(Me)-*L*-^{Me}*N*-Ach-*D*-Leu-]$_2$} (CP2):** A solution of **CP1** (42 mg, 0.032 mmol) in TFA (1 mL) was successively treated with pentamethylbenzene (21 mg, 0.13 mmol), anisole (21 µL, 0.18 mmol) and HBr in AcOH (0.5 mL, 33% in wt). The resulting mixture was stirred at rt for 1h. The solvent was removed under reduced pressure and the crude was purified by HPLC (Phenomenex Luna 5µ silica, 4-8% MeOH/CH_2Cl_2, 25 min) to give 16.3 mg of the desired cyclic peptide. [White solid, 45%, t_R = 19 min]. **^{1}H NMR** (20 mM, $CDCl_3$, 300 MHz, δ): 9.27 (d, *J* = 9.1 Hz, 2H, NH_{Tyr}), 8.52 (d, *J* = 8.9 Hz, 2H, NH_{Ahf}), 8.24 (d, *J* = 8.9 Hz, 2H, NH_{Leu}), 7.07 (m, 4H, Ar), 6.79 (m, 4H, Ar), 5.19 (m, 2H, $H\alpha_{Tyr}$), 4.83 (br, 2H, OH), 4.74 (s, 2H, $H\alpha_{Ahf}$), 4.61 (m, 4H, $H\alpha_{Leu}$ + $H\gamma_{Ach}$), 4.47 (dd, J_1 = 9.7 Hz, J_2 = 3.8 Hz, 2H, $H\gamma_{Ahf}$), 4.22 (s, 2H, $H\beta_{Ahf}$), 4.13 (dd, J_1 = 9.4 Hz, J_2 = 4.2 Hz, 2H, $H\delta_{Ahf}$), 3.86 (d, *J* = 8.9 Hz, 2H, $H\delta_{Ahf}$), 3.79 (s, 6H, OMe_{Tyr}), 2.97 (m, 4H, $H\beta_{Tyr}$), 2.48 (s, 6H, NMe), 1.95 (m, 2H, $H\alpha_{ACH}$), 1.85-1.09 (m, 22H, $CH_{2\ Ach}$ + $CH_{2\ Leu}$ + CH_{Leu}), 0.94 (d, *J* = 6.4 Hz, 6H, $CH_{3\ Leu}$), 0.91 (d, *J* = 5.6 Hz, 6H, $CH_{3\ Leu}$). **^{13}C NMR** ($CDCl_3$, 75.4 MHz, δ): 175.0 (CO), 172.2 (CO), 171.4 (CO), 170.4 (CO), 158.9 (C), 130.5 (CH), 128.1 (C), 114.0 (CH), 85.3 (CH), 78.7 (CH), 74.8 (CH_2), 57.9

(CH), 55.5 (CH_3), 51.6 (CH), 50.3 (CH), 43.8 (CH_2), 43.5 (CH), 39.6 (CH_2), 32.8 (CH_2), 29.9 (CH_3), 28.6 (CH_2), 28.1 (CH_2), 25.2 (CH), 24.8 (CH_2), 22.9 (CH_3), 22.8 (CH_3). **FTIR** (298 K, $CHCl_3$): 3399, 3299 (amide A), 3006, 2958, 2933, 2865, 1652 (amide I_{II}), 1629 (amide I), 1513 cm^{-1} (amide II). **MS (ESI)** [m/z(%)]: 1140.6 ($[MNa]^+$, 7), 1117.6 ($[MH]^+$, 8), 578.3 ($[MHK]^{2+}$, 100), 570.3 ($[MHNa]^{2+}$, 40). **HRMS (ESI) calculated** for $C_{58}H_{85}N_8O_{14}$: 1117.6180, **found**: 1117.6227.

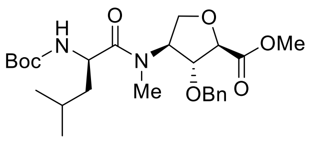

Boc-*D*-Leu-*L*-^{Me}*N*-Ahf(Bn)-OMe (4): A solution of *L*-^{Me}*N*-Ahf(Bn)-OMe2 (600 mg, 2.26 mmol) in dry CH_2Cl_2 (22 mL) was successively treated with DIEA (1.6 mL, 9.0 mmol), Boc-*D*-Leu-OH (523 mg, 2.3 mmol) and *N*-HATU (946 mg, 2.5 mmol). The mixture was stirred at rt under Argon for 90 min. The solution was washed with aqueous HCl (5%, 3 x 20 mL) and aqueous saturated $NaHCO_3$ (2 x 20 mL). The organic layer was dried with anhydrous Na_2SO_4, filtered and concentrated under reduced pressure. The resulting crude was purified by flash chromatography (0-2% MeOH/CH_2Cl_2) to give 1.0 g of dipeptide **4**. [Yellow foam, 93%, R_f = 0.53 (5% MeOH/CH_2Cl_2)]. **^{1}H NMR** ($CDCl_3$, 300 MHz, δ): 7.44-7.19 (m, 5H), 5.17 (m, 1H), 5.00 (m, 1H), 4.76 (AB, *J* = 11.9 Hz, 1H), 4.68-4.31 (m, 1H), 4.59 (AB, *J* = 12.0 Hz, 1H), 4.38 (d, *J* = 4.5 Hz, 1H), 4.19-3.98 (m, 3H), 3.72 (s, 3H), 2.79 (s, 3H), 1.80-1.25 (m, 3H), 1.42 (s, 9H), 0.98 (d, *J* = 6.7 Hz, 3H), 0.93 (d, *J* = 6.7 Hz, 3H). **^{13}C NMR** ($CDCl_3$, 75.4 MHz, δ): 173.5 (CO), 170.6 (CO), 155.7 (CO), 137.3 (C), 128.3 (CH), 128.0 (CH), 127.8 (CH), 85.5 (CH), 82.4 (CH), 79.5 (C), 71.9 (CH_2), 69.3 (CH_2), 61.1 (CH), 52.3 (CH_3), 49.2 (CH), 42.2 (CH_2), 31.5 (CH_3), 28.3 (CH_3), 24.6 (CH), 23.4 (CH_3), 21.6 (CH_3). **MS (ESI)** [m/z(%)]: 501.2 ($[MNa]^+$, 100), 423.2 ($[MH\text{-}^tBu]^+$, 13), 379.2 ($[MH\text{-}Boc]^+$, 6). **HRMS (ESI) calculated** for $C_{25}H_{38}N_2NaO_7$: 501.2571, **found**: 501.2581.

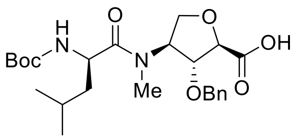

Boc-*D*-Leu-*L*-^{Me}*N*-Ahf(Bn)-OH (dp5): A solution of dipeptide **4** (700 mg, 1.5 mmol) in a mixture of MeOH and water (3:1, 29 mL) was treated with LiOH (175 mg, 7.3 mmol). The solution was stirred at rt for 2 h and then the solvent was removed under reduced pressure. The resulting residue was diluted with water (15 mL), washed with Et_2O (10 mL) and the resulting aqueous solution was acidified to pH 3 with aqueous HCl (5%). The acidic solution was extracted with CH_2Cl_2 (4 x 10 mL) and the combined organic extracts were dried with anhydrous Na_2SO_4, filtered and concentrated under reduced pressure to give 670 mg of the desired dipeptide. [White foam, 99%, R_f = 0.32 (5% MeOH/CH_2Cl_2)]. **^{1}H RMN** ($CDCl_3$, 300 MHz, δ): 9.70 (br, 1H), 7.52-7.10 (m, 5H),

[2] N. Rodríguez-Vázquez, S. Salzinger, L. F. Silva, M. Amorín, J. R. Granja, *Eur. J. Org. Chem.* **2013**, *17*, 3477–3493.

5.61 (m, 1H), 4.90 (m, 1H), 4.75 (AB, *J* = 11.9 Hz, 1H), 4.68-4.40 (m, 1H), 4.63 (AB, *J* = 11.6 Hz, 1H), 4.43 (dd, J_1 = 4.3 Hz, J_2 = 1.4 Hz, 1H), 4.26 (m, 1H), 4.11 (m, 2H), 3.04 (s, 3H), 1.78-1.23 (m, 3H), 1.41 (s, 9H), 0.96 (d, *J* = 6.5 Hz, 3H), 0.92 (d, *J* = 6.6 Hz, 3H). **^{13}C RMN** ($CDCl_3$, 75.4 MHz, δ): 174.3 (CO), 172.8 (CO), 156.2 (CO), 137.4 (C), 128.5 (CH), 128.0 (CH), 127.9 (CH), 86.4 (CH), 82.5 (CH), 80.1 (C), 72.2 (CH_2), 69.4 (CH_2), 62.3 (CH), 49.6 (CH), 41.9 (CH_2), 32.3 (CH_3), 28.4 (CH_3), 24.7 (CH), 23.5 (CH_3), 21.7 (CH_3). **MS (ESI)** [m/z(%)]: 487.2 ([MNa]$^+$, 100), 409.2 ([MH-^{t}Bu]$^+$, 21), 365.2 ([MH-Boc]$^+$, 16). **HRMS (ESI) calculated** for $C_{24}H_{36}N_2NaO_7$: 487.2415, **found**: 487.2395.

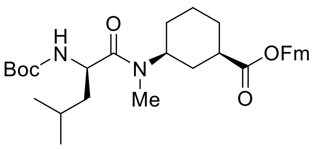

Boc-*D*-Leu-*L*-^{Me}*N*-Ach-OFm (dp4): A solution of Boc-*L*-^{Me}*N*-Ach-OFm (700 mg, 1.6 mmol) in a mixture of TFA and CH_2Cl_2 (1:1, 8 mL) was stirred at rt for 15 min. After removal of the solvent under vacuum, the residue was dried under high vacuum for 3 h. The resulting TFA salt was dissolved under Argon in dry CH_2Cl_2 (16 mL), and DIEA (1.1 mL, 6.4 mmol), Boc-*D*-Leu-OH (372 mg, 1.6 mmol), and *N*-HATU (669 mg, 1.8 mmol) were successively added. After 1 h stirring at rt, the solution was washed with aqueous HCl (5%, 2 x 15 mL) and aqueous saturated $NaHCO_3$ (2 x 15 mL). The organic layer was dried with anhydrous Na_2SO_4, filtered and concentrated under reduced pressure, providing a yellow oil that was purified by flash chromatography (30% EtAcO/hexanes) to give 755 mg of the dipeptide. [White foam, 86%, R_f = 0.65 (50% EtAcO/hexanes)]. **^{1}H NMR** ($CDCl_3$, 300 MHz, δ): 7.76 (d, *J* = 7.6 Hz, 2H), 7.57 (d, *J* = 7.6 Hz, 2H), 7.36 (td, J_1 = 25.0 Hz, J_2 = 7.6 Hz, 4H), 5.31 (d, *J* = 9.2 Hz, 1H), 4.64 (m, 1H), 4.53-4.35 (m, 3H), 4.19 (t, *J* = 6.7 Hz, 1H), 2.90 and 2.79 (s, 3H), 2.08-1.12 (m, 11H), 1.42 (s, 9H), 0.99 (dd, *J* = 6.5 Hz, *J* = 1.9 Hz, 3H), 0.91 (dd, *J* = 6.7 Hz, *J* = 1.4 Hz, 3H).**^{13}C NMR** ($CDCl_3$, 75.4 MHz, δ): 174.9 (CO), 172.8 (CO), 155.7 (CO), 143.8 (C), 141.4 (C), 127.9 (CH), 127.2 (CH), 125.1 (CH), 120.1 (CH), 79.5 (C), 66.2 (CH_2), 55.1 (CH), 51.7 (CH), 49.2 (CH), 47.1 (CH), 42.9 (CH_2), 42.6 (CH), 32.8 (CH_2), 31.6 (CH_2), 29.3 (CH_3), 29.0 (CH_2), 28.5 (CH_3), 24.8 (CH), 24.5 (CH_2), 23.6 (CH_3), 21.8 (CH_3). **MS (ESI)** [m/z(%)]: 571.3 ([MNa]$^+$, 24), 549.3 ([MH]$^+$, 11), 493.3 ([MH-^{t}Bu]$^+$, 100), 449.3 ([MH-Boc]$^+$, 100). **HRMS (ESI) calculated** for $C_{33}H_{45}N_2O_5$: 549.3323, **found**: 549.3323.

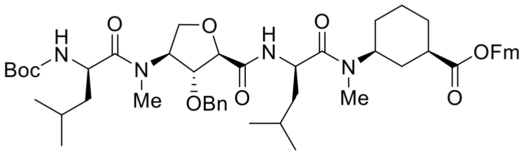

Boc-*D*-Leu-*L*-^{Me}*N*-Ahf(Bn)-*D*-Leu-*L*-^{Me}*N*-Ach-OFm (tp4): A solution of dipeptide **dp4** (700 mg, 1.2 mmol) in a mixture of TFA and CH_2Cl_2 (1:1, 10 mL) was stirred at rt for 30 min. After removal of the solvent under vacuum, the residue was dried under high vacuum for 3 h. The resulting TFA salt (**dp6**) was dissolved in dry CH_2Cl_2 (12 mL) under Argon, then DIEA (850 µL, 4.8 mmol), dipeptide **dp5** (562 mg, 1.2 mmol) and *N*-HBTU (504 mg, 1.3 mmol) were successively added. After 2 h stirring at rt, the solution was washed with

aqueous HCl (5%, 2 x 10 mL) and aqueous saturated $NaHCO_3$ (2 x 10 mL). The organic layer was dried with anhydrous Na_2SO_4, filtered and concentrated under reduced pressure. The resulting crude was purified by flash chromatography (0-2% MeOH/CH_2Cl_2) to give 1.1 g of the tetrapeptide **tp4**. [White foam, 99%, R_f = 0.41 (2% MeOH/CH_2Cl_2)]. **MS (ESI)** [m/z(%)]: 933.4 ([MK]$^+$, 3), 917.5 ([MNa]$^+$, 82), 895.5 ([MH]$^+$, 100), 795.5 ([MH-Boc]$^+$, 4). **HRMS (ESI) calculated** for $C_{52}H_{71}N_4O_9$: 895.5216, **found**: 895.5204.

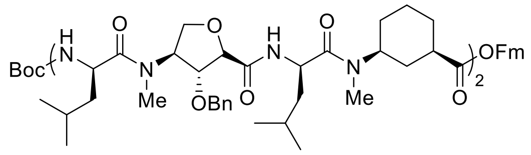

Boc-[*D*-Leu-*L*-^{Me}*N*-Ahf(Bn)-*D*-Leu-*L*-^{Me}*N*-Ach]$_2$-OFm (op3): A solution of tetrapeptide **tp4** (515 mg, 0.46 mmol) in a mixture of piperidine and CH_2Cl_2 (1:4, 5 mL) was stirred at rt for 45 min. The solution was washed with aqueous HCl (5%, 4 x 10 mL), dried with anhydrous Na_2SO_4, filtered and concentrated, to give Boc-*D*-Leu-*L*-^{Me}*N*-Ahf(Bn)-*D*-Leu-*L*-^{Me}*N*-Ach-OH (**tp5**), which was used without further purification.

A solution of tetrapeptide **tp4** (515 mg, 0.46 mmol) in a mixture of TFA and CH_2Cl_2 (1:1, 6 mL) was stirred at rt for 30 min. After removal of the solvent under vacuum, the residue was dried under high vacuum for 3 h. The resulting TFA salt (**tp6**) was dissolved in dry CH_2Cl_2 (5 mL) under Argon, and successively treated with DIEA (320 µL, 1.9 mmol), the previously prepared **tp5** and *N*-HBTU (193 mg, 0.51 mmol). After 2 h stirring at rt, the solution was washed with aqueous HCl (5%, 2 x 10 mL) and aqueous saturated $NaHCO_3$ (2 x 10 mL). The organic layer was dried with anhydrous Na_2SO_4, filtered and concentrated under reduced pressure. The resulting crude was purified by flash chromatography (0-5% MeOH/CH_2Cl_2) to give 683 mg of the desired octapeptide (**op3**). [White foam, 98%, R_f = 0.35 (2% MeOH/CH_2Cl_2)]. **MS (ESI)** [m/z(%)]: 1531.8 ([MK]$^+$, 1), 1516.9 ([MNa]$^+$, 24), 1493.9 ([MH]$^+$, 10), 774.7 (100), 769.9 ([MNa]$^{2+}$, 24), 697.4 ([MH-Boc]$^{2+}$, 97). **HRMS (ESI) calculated** for $C_{85}H_{121}N_8O_{15}$: 1493.8946, **found**: 1493.8971.

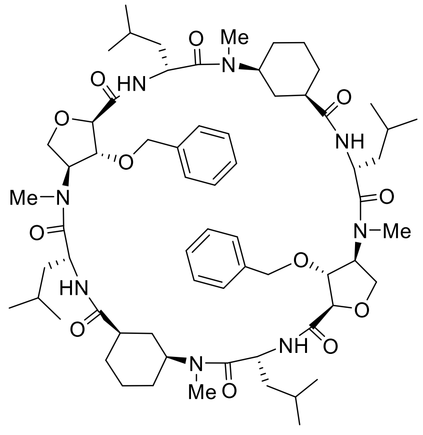

***c*-{[*L*-^{Me}*N*-Ahf(Bn)-*D*-Leu^1-*L*-^{Me}*N*-Ach-*D*-Leu^2-]$_2$} (CP3):** The linear octapeptide **op3** (400 mg, 0.27 mmol) was dissolved in a mixture of piperidine and CH_2Cl_2 (1:4, 5 mL) and stirred at rt for 45 min. The solution was washed with aqueous HCl (5%, 4 x 10 mL), dried with anhydrous Na_2SO_4, filtered and concentrated to dryness to give Boc-[*D*-Leu-*L*-^{Me}*N*-Ahf(Bn)-*D*-Leu-*L*-^{Me}*N*-Ach]$_2$-OH, which was used without further purification. The resulting octapeptide was dissolved in a mixture of TFA and CH_2Cl_2 (1:1, 6 mL) and stirred at rt for 20 min. After removal of the solvent under reduced pressure, the residue was dried under high vacuum for 3 h and used without

further purification. The resulting unprotected linear peptide (**op4**) was dissolved in dry CH_2Cl_2 (268 mL) and successively treated with DIEA (280 µL, 1.6 mmol) and *N*-TBTU (112 mg, 0.29 mmol). After 12 h, the solvent was removed under reduced pressure and the residue was dissolved in CH_2Cl_2 (25 mL), washed with aqueous HCl (10%, 2 x 20 mL), dried with anhydrous Na_2SO_4, filtered and concentrated to dryness. The crude was purified by flash chromatography (0-6% MeOH/CH_2Cl_2) to give 200 mg of **CP3**. [White solid, 62%, R_f = 0.37 (5% MeOH/CH_2Cl_2)]. **^{1}H NMR** (16 mM, $CDCl_3$, 500 MHz, δ): 9.08 (d, *J* = 9.4 Hz, 1.33H, $NH_{Leu1\text{-}D3E}$), 8.79 (d, *J* = 9.4 Hz, 0.66H, $NH_{Leu1\text{-}D3A}$), 8.08 (d, *J* = 9.2 Hz, 1.33H, $NH_{Leu2\text{-}D3E}$), 8.03 (d, *J* = 9.6 Hz, 0.66H, $NH_{Leu2\text{-}D3A}$), 6.96 (br, 4.66H, Bn), 6.68 (br, 4H, Bn), 6.58 (m, 1.33H, Bn), 5.53 (m, 1.33H, $H\gamma_{Ahf\text{-}D3E}$), 5.45 (m, 0.66H, $H\gamma_{Ahf\text{-}D3A}$), 5.29 (m, 2H, $H\alpha_{Leu1}$), 5.21 (d, *J* = 5.4 Hz, 0.66H, $H\alpha_{Ahf\text{-}D3A}$), 5.13 (m, 2H, $H\alpha_{Leu2}$), 4.78 (d, *J* = 8.3 Hz, 1.33H, $H\alpha_{Ahf\text{-}D3E}$), 4.53 (m, 1.33H, $H\gamma_{Ach\text{-}D3E}$), 4.37 (m, 1.33H, $H\beta_{Ahf\text{-}D3A}$ + $H\delta_{Ahf\text{-}D3A}$), 4.22 (dd, J_1 = 10.2 Hz, J_2 = 8.8 Hz, 1.33H, $H\beta_{Ahf\text{-}D3E}$), 4.06 (m, 2.66H, $H\delta_{Ahf\text{-}D3E}$ + $H\gamma_{Ach\text{-}D3A}$ + $H\delta_{Ahf\text{-}D3A}$), 3.96 (br, 2.66H, $H\beta_{Bn\text{-}D3E}$), 3.73 (dd, J_1 = 10.7 Hz, J_2 = 9.3 Hz, 1.33H, $H\delta_{Ahf\text{-}D3E}$), 3.66 (AB, *J* = 9.9 Hz, 0.66H, $H\beta_{Bn\text{-}D3A}$), 3.60 (AB, *J* = 9.8 Hz, 0.66H, $H\beta_{Bn\text{-}D3A}$), 3.33 (s, 2H, $NMe_{Ahf\text{-}D3A}$), 3.04 (s, 4H, $NMe_{Ach\text{-}D3E}$), 2.80 (m, 1.33H, $H\alpha_{Ach\text{-}D3E}$), 2.67 (s, 2H, $NMe_{Ach\text{-}D3A}$), 2.62 (s, 4H, $NMe_{Ahf\text{-}D3E}$), 2.31 (m, 0.66H, $H\alpha_{Ach\text{-}D3A}$), 1.91-1.05 (m, 27.33H, $CH_{2\ Ach}$ + $CH_{2\ Leu}$ + CH_{Leu}), 1.04-0.80 (m, 24H, $CH_{3\ Leu}$), -1.20 (d, *J* = 11.5 Hz, 0.66H, $H\beta_{Ach\text{-}D3A}$). **^{13}C NMR** ($CDCl_3$, 75.4 MHz, δ): 175.2 (CO), 175.0 (CO), 174.4 (CO), 173.8 (CO), 172.4 (CO), 171.8 (CO), 171.4 (CO), 169.5 (CO), 136.8 (C), 136.0 (C), 128.7 (CH), 128.4 (CH), 128.1 (CH), 127.3 (CH), 127.0 (CH), 82.8 (CH), 82.3 (CH), 79.9 (CH), 77.1 (CH), 72.2 (CH_2), 71.8 (CH_2), 67.9 (CH_2), 66.2 (CH_2), 59.6 (CH), 57.7 (CH), 51.5 (CH), 51.1 (CH), 47.0 (CH), 46.6 (CH), 46.5 (CH), 43.9 (CH_2), 43.5 (CH_2), 31.4 (CH), 30.7 (CH_2), 30.1 (CH_2), 29.8 (CH), 29.3 (CH), 29.0 (CH), 28.7 (CH_2), 25.4 (CH_2), 25.0 (CH_2), 24.7 (CH_3), 24.6 (CH_3), 24.5 (CH_3), 23.7 (CH_3), 23.4 (CH_3), 23.2 (CH_3), 22.5 (CH), 22.1 (CH_3), 21.7 (CH_3). **FTIR** (298 K, $CHCl_3$): 3307 (amide A), 3066, 2954, 2925, 2866, 1678 (amide I_{II}), 1626 (amide I), 1526 cm^{-1} (amide II). **MS (ESI)** [m/z(%)]: 1219.7 ($[MNa]^+$, 10), 1197.7 ($[MH]^+$, 100), 945.6 (30), 683.6 (60), 599.4 ($[MH]^{2+}$, 9). **HRMS (ESI) calculated** for $C_{66}H_{101}N_8O_{12}$: 1197.7533, **found**: 1197.7556.

X-Ray Crystallographic Determination of $D3_A$

Preparation of single crystals for X-Ray analysis: In a typical experiment, 1.0 mg of pure **CP3** was dissolved in 900 µL of $CHCl_3$, and equilibrated by vapour-phase diffusion against 3.0 mL of hexanes. The corresponding dimer crystallized spontaneously after 7 days.

X-Ray Crystallographic Analysis: Data were collected at 100 K, using Bruker X8 APEXII CCD diffractometer. All calculations were performed on a PC compatible computer using the programs: SORTAV (Blessing, 1995), SHELXT-2014 (Sheldrick, 2014), SHELXL204/7 (Sheldrick,

2014), ORTEP-3 (Farrugia, 2012), WinGX (Farrugia, 2012). Supplementary crystallographic data for D3$_A$ (CIF format) can be obtained free of charge from the journal.
The crystal structure was deposited at the Cambridge Crystallographic Data Centre, and the data was assigned to the following deposition number: CCDC 1400134.

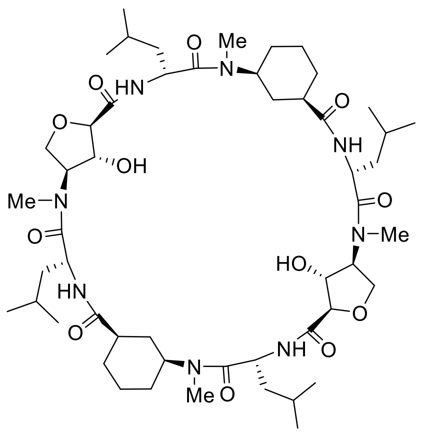

***c*-{[*L*-^{Me}*N*-Ahf-*D*-Leu^1-*L*-^{Me}*N*-Ach-*D*-Leu^2-]$_2$} (CP4):** A solution of **CP3** (50 mg, 0.042 mmol) and Pd/C (36 mg, 10% in wt) in a mixture of MeOH and CH_2Cl_2 (3:1, 20 mL) was stirred at rt for 20 h under hydrogen atmosphere (balloon pressure). The resulting mixture was filtered through a Celite pad, the residue was washed with CH_2Cl_2, and the combined filtrates and washes were concentrated under reduced pressure. The crude was purified by HPLC (Phenomenex Luna 5µ silica, 6-13% MeOH/CH_2Cl_2, 30 min) to give 30 mg of the cyclic peptide **CP4**. [White solid, 71%, t_R = 22 min]. **^{1}H NMR** (5 mM, $CDCl_3$, 298 K, 300 MHz, δ): 7.92 (br, 2H), 5.31 (br, 2H), 5.08 (m, 4H), 4.47 (m, 6H), 4.23 (br, 2H), 3.83 (dd, J_1 = 10.7 Hz, J_2 = 9.0 Hz, 2H), 3.08 (s, 6H), 3.03 (s, 6H), 2.79 (m, 2H), 2.01-1.10 (m, 28H), 1.03-0.80 (m, 24H). **^{1}H NMR** (32 mM, $CDCl_3$, 253 K, 500 MHz, δ): 8.72 (d, J = 9.1 Hz, 0.8H, NH_{Leu1}), 8.47 (d, J = 9.1 Hz, 1.2H, $NH_{Leu1\text{-}D4A}$), 7.95 (d, J = 8.6 Hz, 0.8H, NH_{Leu2}), 7.72 (d, J = 9.4 Hz, 1.2H, $NH_{Leu2\text{-}D4A}$), 5.97 (br, 0.8H, OH), 5.48 (br, 1.2H, OH), 5.32 (m, 2H, $H\gamma_{Ahf}$), 5.09 (m, 4H, $H\alpha_{Leu}$), 4.76 (br, 1H, $H\alpha_{Ahf\text{-}D4A}$), 4.45 (m, 5H, $H\gamma_{Ach}$ + $H\beta_{Ahf}$ + $H\alpha_{Ahf}$), 3.98 (m, 4H, $H\delta_{Ahf}$), 3.13 (s, 2H, NMe), 3.07 (s, 7H, NMe_{Ahf} + $H\alpha_{Ach}$), 2.98 (s, 4H, NMe_{Ach}), 2.62 (m, 1H, $H\alpha_{Ach}$), 2.07-1.14 (m, 28H, $CH_{2\ Ach}$ + $CH_{2\ Leu}$ + CH_{Leu}), 1.07-0.68 (m, 24H, $CH_{3\ Leu}$). **^{1}H NMR** (32 mM, 5% CD_3OH/$CDCl_3$, 253 K, 500 MHz, δ): 8.83 (d, J = 9.3 Hz, 0.2H, NH_{Leu1}), 8.64 (d, J = 8.9 Hz, 1.8H, $NH_{Leu1\text{-}D4A}$), 8.15 (d, J = 9.5 Hz, 1.8H, $NH_{Leu2\text{-}D4A}$), 7.94 (d, J = 8.0 Hz, 0.2H, NH_{Leu2}), 6.11 (s, 1.8H, OH), 5.76 (s, 0.2H, OH), 5.31 (m, 2H, $H\gamma_{Ahf}$), 5.13 (m, 4H, $H\alpha_{Leu}$), 4.62 (d, J = 7.9 Hz, 2H, $H\alpha_{Ahf}$), 4.57 (m, 2H, $H\gamma_{Ach}$), 4.31 (dd, J_1 = 9.7 Hz, J_2 = 8.7 Hz, 2H, $H\beta_{Ahf}$), 4.05 (m, 0.2H, $H\delta_{Ahf}$), 3.96 (d, J = 9.6 Hz, 3.6H, $H\delta_{Ahf\text{-}D4A}$), 3.84 (m, 0.2H, $H\delta_{Ahf}$), 3.37 (s, CH_3OH), 3.09 (s, 5.4H, $NMe_{Ahf\text{-}D4A}$), 3.06 (s, 0.6H, NMe_{Ahf}), 2.93 (s, 6H, NMe_{Ach}), 2.78 (m, 2H, $H\alpha_{Ach}$), 1.97-1.15 (m, 28H, $CH_{2\ Ach}$ + $CH_{2\ Leu}$ + CH_{Leu}), 0.98 (d, J =6.3 Hz, 4H, $CH_{3\ Leu}$), 0.87 (dd, J_1 =8.1 Hz, J_2 = 6.8 Hz, 16H, $CH_{3\ Leu}$), 0.82 (d, J =6.9 Hz, 4H, $CH_{3\ Leu}$). **FTIR** (298 K, $CHCl_3$): 3437, 3298 (amide A), 2955, 2931, 2867, 1676 (amide I_{II}), 1621 (amide I), 1537 cm^{-1} (amide II). **MS (ESI)** [m/z(%)]: 1017.6 ($[MH]^+$, 16), 536.8 (32), 528.3 ($[MK]^{2+}$, 21), 509.3 ($[MH]^{2+}$, 9), 381.3 (100). **HRMS (ESI) calculated** for $C_{52}H_{89}N_8O_{12}$: 1017.6594, **found**: 1017.6556.

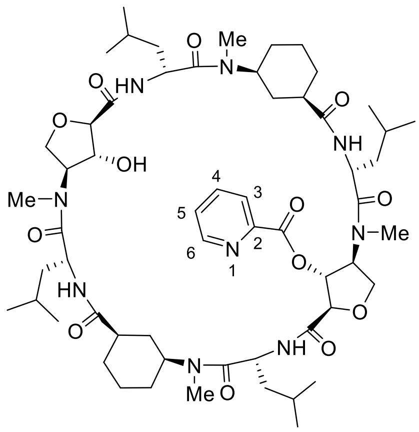

***c*-[*L*-^{Me}*N*-Ahf(pic)-*D*-Leu^{1}-*L*-^{Me}*N*-Ach^{1}-*D*-Leu^{2}-*L*-^{Me}*N*-Ahf-*D*-Leu^{3}-*L*-^{Me}*N*-Ach^{2}-*D*-Leu^{4}-] (CP5):** A solution of **CP4** (5.5 mg, 5.4 µmol) in CH_2Cl_2 (1 mL) was treated with 2-picolinic acid (0.73 mg, 5.9 µmol), EDC·HCl (1.1 mg, 5.9 µmol), HOBt (0.79 mg, 5.9 µmol) and DMAP (0.72 mg, 5.9 µmol). Each 12 h, additional 2-picolinic acid (1.5 mg, 11.8 µmol), EDC (2.2 mg, 11.8 µmol) and DMAP (1.4 mg, 11.8 µmol) was added during 3-4 days. Then, the solution was washed with aqueous saturated $NaHCO_3$ (2 x 5 mL) and aqueous HCl (5%, 2 x 5 mL). The organic layer was dried with anhydrous Na_2SO_4, filtered and concentrated to dryness. The crude was purified by HPLC (Phenomenex Luna 5µ silica, 7-11% MeOH/CH_2Cl_2, 30 min) to give 3.2 mg of the cyclic peptide **CP5**. [White solid, 54%, t_R = 20 min]. **^{1}H NMR** ($CDCl_3$, 300 MHz, δ): 8.80-8.15 (m, 3H), 7.99-7.56 (m, 3H), 7.43 (m, 1H), 7.11 (m, 0.5H), 6.72 (m, 0.5H), 6.06-3.67 (m, 17H), 3.35 (s, 1.5H), 3.21 (s, 1H), 3.20 (s, 2H), 3.17 (s, 1H), 3.13 (s, 1H), 3.01 (s, 4H), 2.84 (s, 1.5H), 2.37 (m, 2H), 2.12-0.54 (m, 52H). **FTIR** (298 K, $CHCl_3$): 3298 (amide A), 3068, 3003, 2956, 2929, 2868, 1747 (C=O), 1683 (amide I_{II}), 1623 (amide I), 1540 cm^{-1} (amide II). **MS (ESI)** [m/z(%)]: 1144.7 ([MNa]$^+$, 23), 1122.7 ([MH]$^+$, 32), 572.8 ([MNa]$^{2+}$, 8), 511.3 (34), 500.3 (100). **HRMS (ESI) calculated** for $C_{58}H_{92}N_9O_{13}$: 1122.6809, **found**: 1122.6807.

ADDITION EXPERIMENT OF $AgBF_4$:

CP5 (2.2 mg, 1.9 µmol) was dissolved in $CDCl_3$ (400 µL) in a NMR tube. In a flask under Ar and protected from light, $AgBF_4$ (15.8 mg, 0.081 mmol) (dry box) was dissolved in CD_3CN (2.0 mL). Then, different portions (0.1, 0.2, 0.3, 0.4 and 0.5 equiv) of the solution of $AgBF_4$ (24 µL, 0.95 µmol) were added to the NMR tube to form mainly the complex ***s*-D5$_E$⊃Ag**. **^{1}H NMR** (4.5 mM, $CDCl_3$, 500 MHz, δ): 8.66 (d, *J* = 9.1 Hz, 0.25H, NH_{minor}), 8.55 (d, *J* = 9.2 Hz, 1H, $NH_{Leu3(major)+minor}$), 8.43 (d, *J* = 9.6 Hz, 0.75H, $NH_{Leu1(major)}$), 7.99 (d, *J* = 9.1 Hz, 0.75H, $NH_{Leu4(major)}$), 7.93 (d, *J* = 9.2 Hz, 0.75H, $NH_{Leu2(major)}$), 7.87 (d, *J* = 4.3 Hz, 0.25H, Pic_{minor}), 7.78 (s, 1H, NH_{minor}+ H6-Pic_{major}), 7.65 (d, *J* = 9.1 Hz, 0.25H, NH_{minor}), 7.51 (m, 1H, H3-Pic_{major}+Pic_{minor}), 7.23 (m, 0.25H, Pic_{minor}), 7.10 (m, 1.50H, H4/H5-Pic_{major}), 6.93 (m, 0.25H, Pic_{minor}), 5.82 (m, 1.50 H, $H\gamma_{Ahf(pic)}$), 5.63 (dd, J_1 = 9.7 Hz, J_2 = 9.2 Hz, 0.75H, $H\beta_{Ahf(pic)}$), 5.41 (m, 1H, $H\gamma_{Ahf}$), 5.27 (m, 1.25H, $H\alpha_{Leu4}$), 5.19 (m, 1.5H, $H\alpha_{Leu3}$ + $H\alpha_{Ahf(pic)(minor)}$), 5.12 (m, 1.25H, $H\alpha_{Leu2}$), 5.07 (m, 1.5H, $H\alpha_{Leu1}$), 4.93 (d, *J* = 9.0 Hz, 0.75H, $H\alpha_{Ahf(pic)}$), 4.81 (br, 0.25H), 4.59 (d, *J* = 8.5 Hz, 1.50H, $H\alpha_{Ahf}$), 4.40 (dd, J_1 = 10.6 Hz, J_2 = 8.9 Hz, 0.75H, $H\beta_{Ahf}$), 4.33 (dd, J_1 = 11.7 Hz, J_2 = 9.6 Hz, 0.75H, $H\delta_{Ahf(pic)}$), 4.27 (m, 0.75H, $H\gamma_{Ach2}$), 4.12 (dd, J_1 = 9.3 Hz, J_2 = 8.8 Hz, 0.75H, $H\delta_{Ahf}$), 4.02 (dd, J_1 = 9.8 Hz, J_2 = 8.3 Hz, 0.75H, $H\delta_{Ahf(pic)}$), 3.97 (dd, J_1 = 10.2 Hz, J_2 = 9.7 Hz, 0.75H, $H\delta_{Ahf}$), 3.92 (m, 0.75H, $H\gamma_{Ach1}$), 3.82 (m, 0.5H), 3.28 (s, 2.25H, $NMe_{Ahf(pic)}$), 3.16 (s,

0.75H, NMe), 3.14 (s, 0.75H, NMe), 3.13 (s, 2.25H, NMe_{Ahf}), 3.06 (s, 2.25H, NMe_{Ach2}), 2.96 (s, 0.75H), 2.94 (s, 0.75H), 2.82 (s, 2.25H, NMe_{Ach1}), 2.57 (m, 0.75H, $H\alpha_{Ach2(major)}$), 2.48 (m, 0.25H, $H\alpha_{Ach(minor)}$), 2.35 (m, 0.75H, $H\alpha_{Ach1(major)}$), 2.26 (m, 0.25H, $H\alpha_{Ach(minor)}$), 2.02-1.17 (m, 27H, $CH_{2\ Ach}$ + $CH_{2\ Leu}$ + CH_{Leu}), 1.04-0.77 (m, 24H, $CH_{3\ Leu}$), 0.11 (d, *J* = 9.0 Hz, 0.75H, $H\beta_{Ach1(major)}$). **MS (ESI)** [m/z(%)]: 2957.6 (35), 2546.2 (Dimer+Ag+$AgBF_4$, 71), 2352.3 (Dimer+Ag, 100), 2247.2 (Dimer, 28), 2057.3 (28), 1230.6 (CP+Ag, 19), 942.1 (17). **HRMS (ESI) calculated** for $C_{116}H_{182}AgN_{18}O_{16}$: 2350.2518, **found**: 2350.2604.

ADDITION EXPERIMENT OF OXALIC ACID:

CP5 (2.5 mg, 2.3 µmol) was dissolved in $CDCl_3$ (400 µL) in a NMR tube. In another flask, $(CO_2H)_2 \cdot 2H_2O$ (7.9 mg, 0.063 mmol) was dissolved in CD_3CN (1.0 mL). Then, different portions (0.1, 0.2, 0.3, 0.4 and 0.5 equiv) of the solution of $(CO_2H)_2 \cdot 2H_2O$ (17.8 µL, 1.15 µmol) were added to the NMR tube to form mainly the complex ***s*-D5$_E$⊃(CO$_2$H)$_2$**. **^{1}H NMR** (5mM, $CDCl_3$, 500 MHz, δ): 8.65 (d, *J* = 9.2 Hz, 1H, NH_{Leu3}), 8.47 (d, *J* = 9.5 Hz, 1H, NH_{Leu1}), 8.15 (d, *J* = 9.4 Hz, 1H, NH_{Leu4}), 7.96 (S, 1H, H6-Pic), 7.92 (d, *J* = 10.0 Hz, 1H, NH_{Leu2}), 7.48 (br, 1H, H3-Pic), 7.18 (br, 2H, H4/H5-Pic), 5.82 (m, 1H, $H\gamma_{Ahf(pic)}$), 5.60 (dd, J_1 = 10.3 Hz, J_2 = 9.4 Hz, 1H, $H\beta_{Ahf(pic)}$), 5.46 (m, 1H, $H\gamma_{Ahf}$), 5.30 (m, 1H, $H\alpha_{Leu4}$), 5.23 (m, 1H, $H\alpha_{Leu3}$), 5.14 (m, 2H, $H\alpha_{Leu2}$ + OH), 5.08 (m, 1H, $H\alpha_{Leu1}$), 4.92 (d, *J* = 9.1 Hz, 1H, $H\alpha_{Ahf(pic)}$), 4.71 (d, *J* = 8.5 Hz, 1H, $H\alpha_{Ahf}$), 4.49 (dd, J_1 = 11.0 Hz, J_2 = 8.7 Hz, 1H, $H\beta_{Ahf}$), 4.32 (m, 2H, $H\delta_{Ahf(pic)}$ + $H\gamma_{Ach2}$), 4.14 (m, J_1 = 11.5 Hz, J_2 = 9.0 Hz, 1H, $H\delta_{Ahf}$), 4.02 (dd, J_1 = 10.1 Hz, J_2 = 8.9 Hz, 1H, $H\delta_{Ahf(pic)}$), 3.96 (dd, J_1 = 11.1 Hz, J_2 = 9.6 Hz, 1H, $H\delta_{Ahf}$), 3.92 (m, 1H, $H\gamma_{Ach1}$), 3.28 (s, 3H, $NMe_{Ahf(pic)}$), 3.12 (s, 3H, NMe_{Ahf}), 3.08 (s, 3H, NMe_{Ach2}), 2.83 (s, 3H, NMe_{Ach1}), 2.65 (m, 1H, $H\alpha_{Ach2}$), 2.40 (m, 1H, $H\alpha_{Ach1}$), 1.88-1.07 (m, 27H, $CH_{2\ Ach}$ + $CH_{2\ Leu}$ + CH_{Leu}), 1.06-0.70 (m, 24H, $CH_{3\ Leu}$), 0.15 (d, *J* = 11.8 Hz, 1H, $H\beta_{Ach1}$).

NMR and FTIR Spectra:

Boc-*D*-Leu-*L*-Ahf(Bn)-OMe (3): ^{1}H NMR ($CDCl_3$, 298 K, 250 MHz), **DEPT** and **^{13}C NMR** ($CDCl_3$, 298 K, 75.4 MHz).

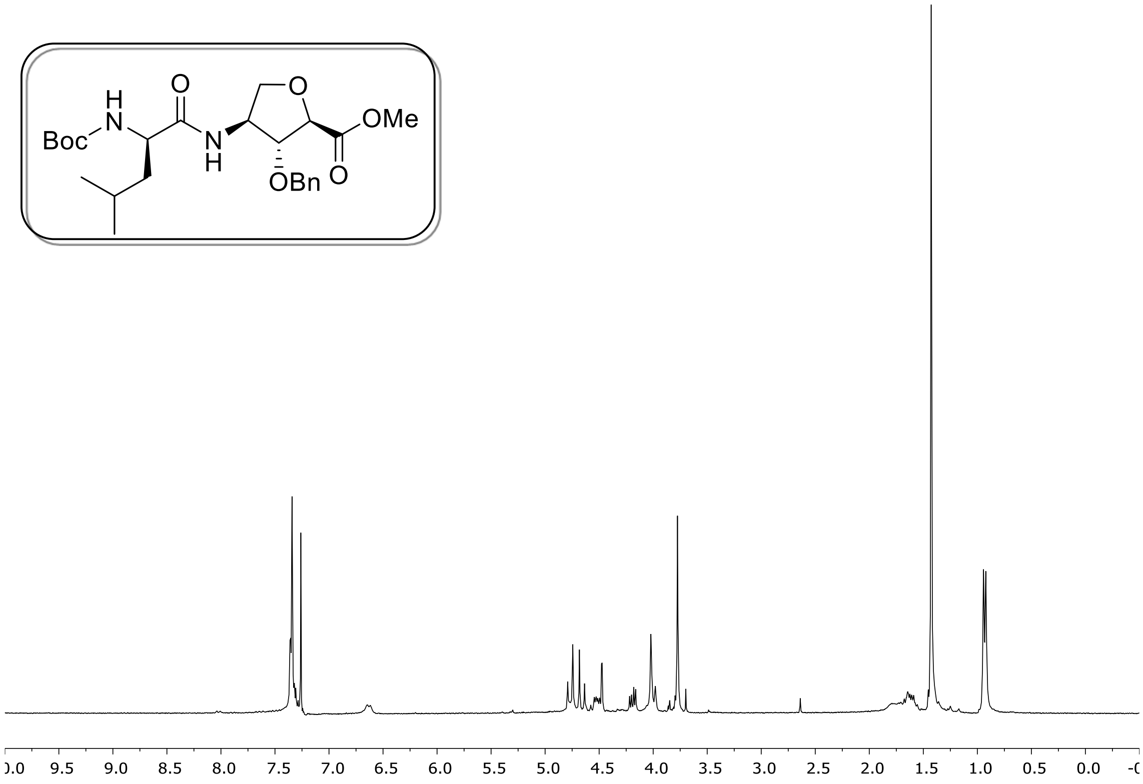

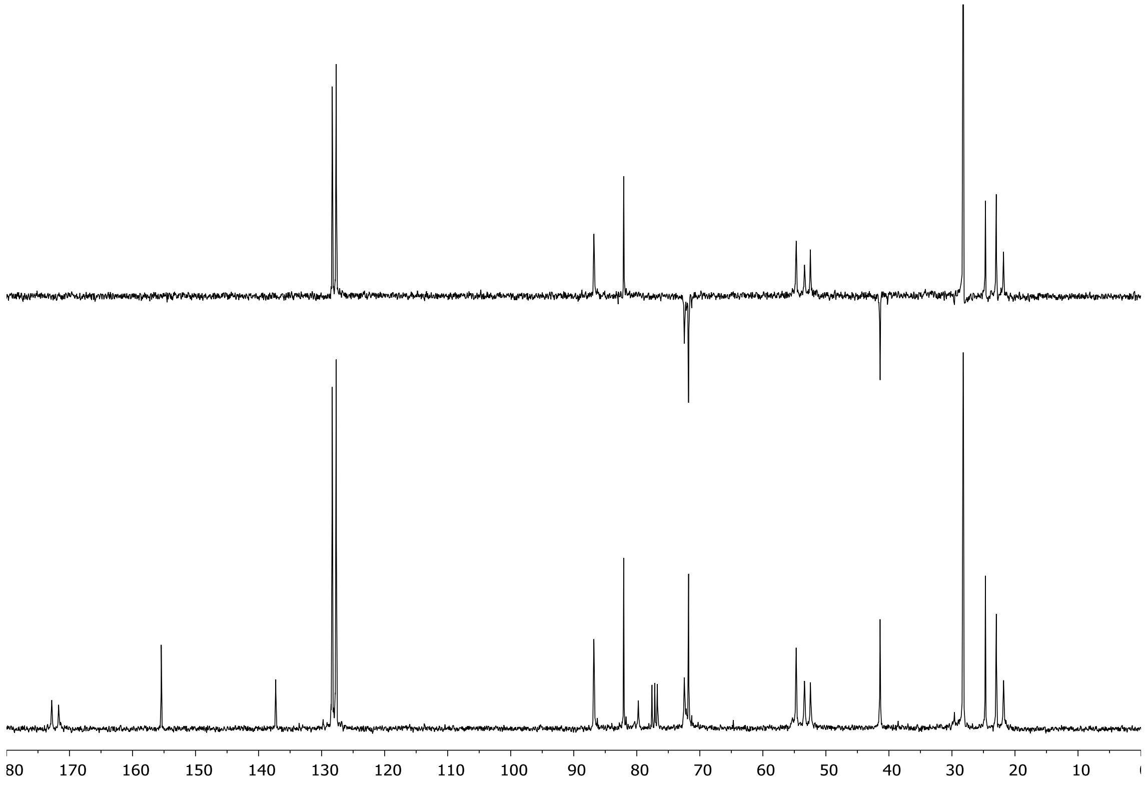

Boc-*D*-Leu-*L*-Ahf(Bn)-OH (dp2): ^{1}H NMR (CDCl$_3$, 298 K, 300 MHz), **DEPT** and **^{13}C NMR** (CDCl$_3$, 298 K, 75.4 MHz).

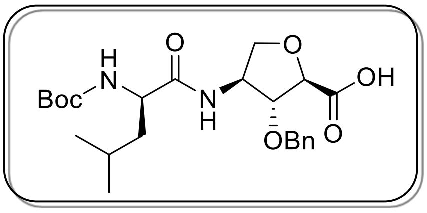

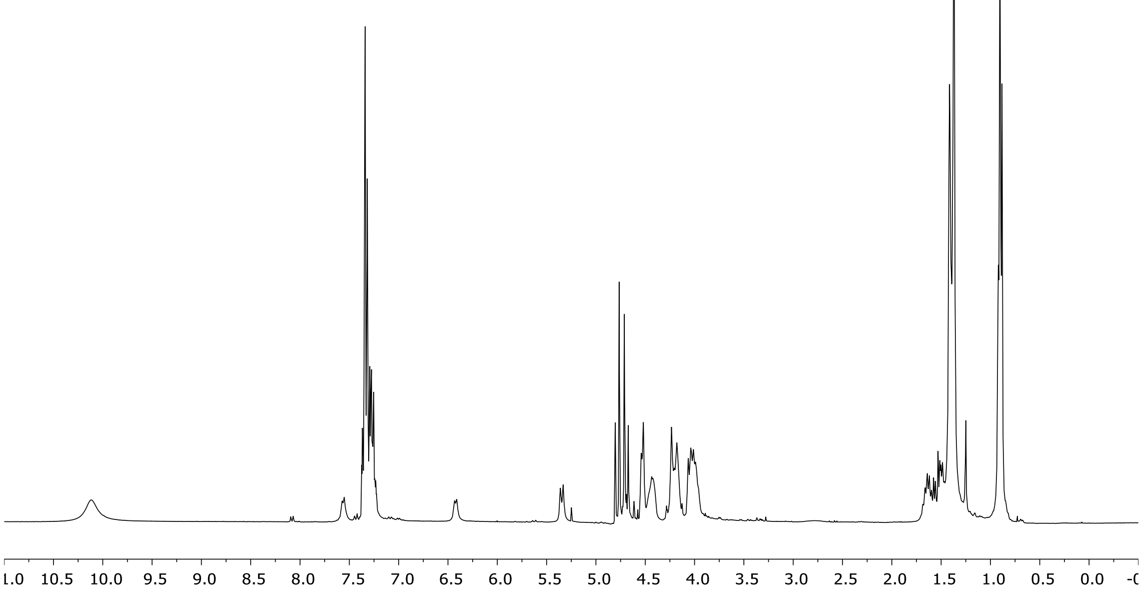

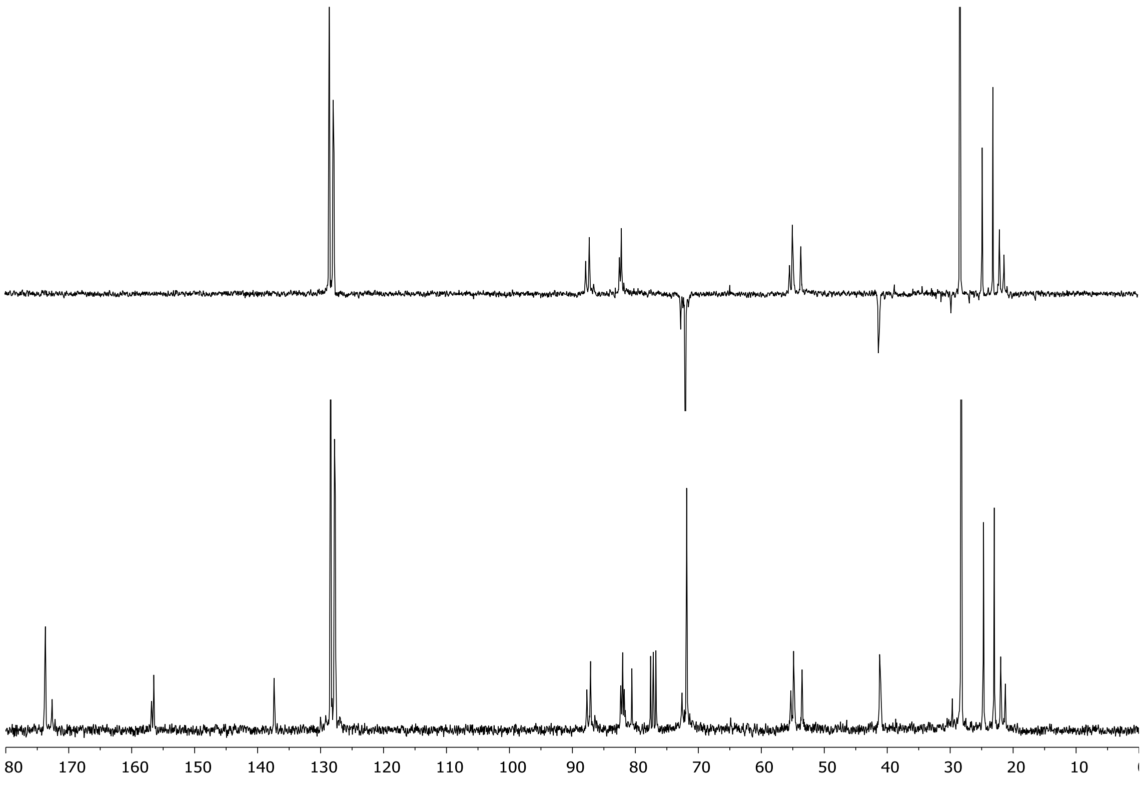

Boc-*D*-Tyr(Me)-*L*-^{Me}*N*-Ach-OFm (dp1): ^{1}H NMR (CDCl$_3$, 298 K, 300 MHz), **DEPT** and **^{13}C NMR** (CDCl$_3$, 298 K, 75.4 MHz).

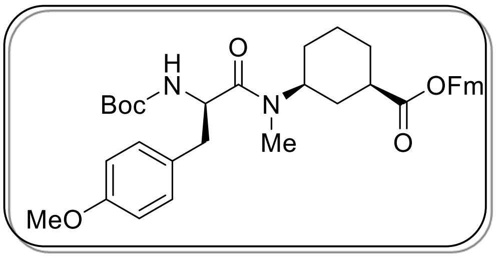

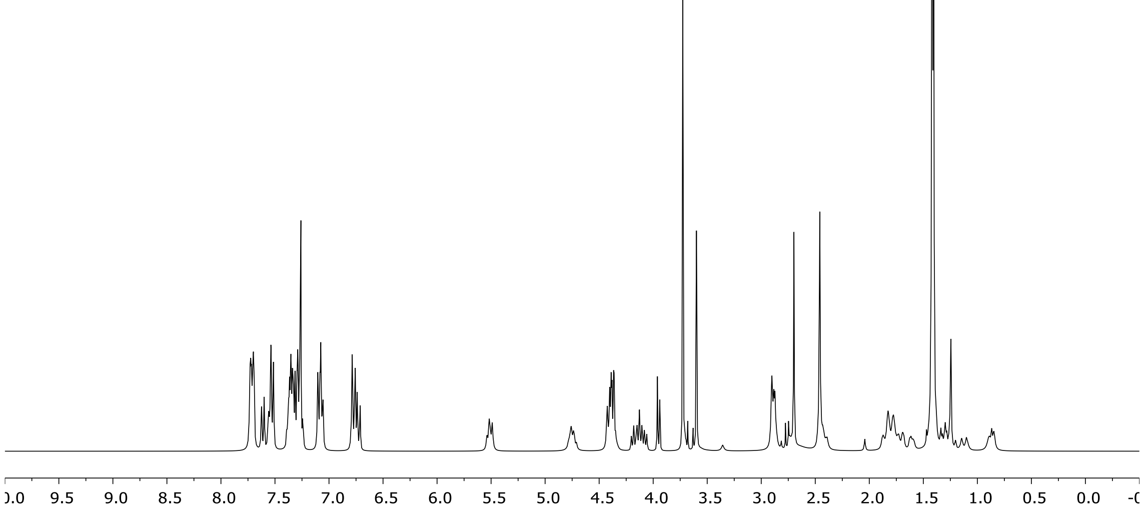

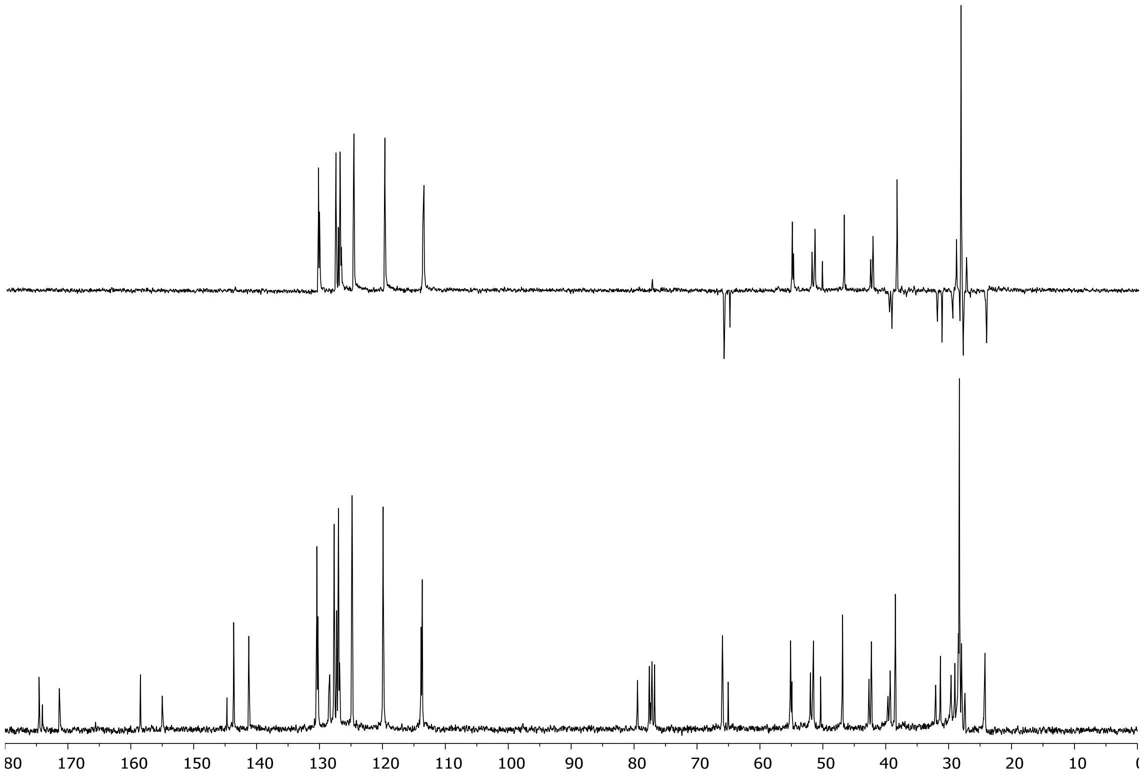

***c*-{[*L*-Ahf(Bn)-*D*-Tyr(Me)-*L*-^{Me}*N*-Ach- *D*-Leu-]$_2$} (CP1):** **^{1}H NMR** (7.5 mM, $CDCl_3$, 298 K, 300 MHz) and **^{13}C NMR** ($CDCl_3$, 298 K, 75.4 MHz).

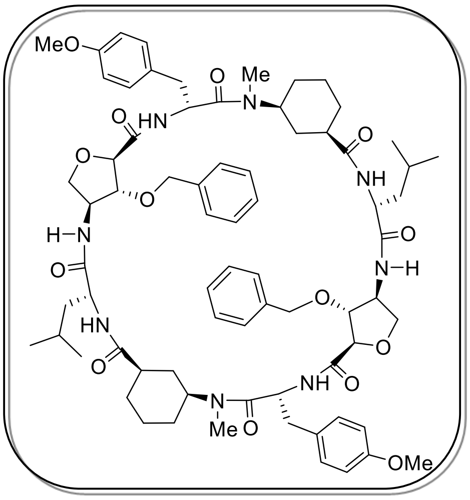

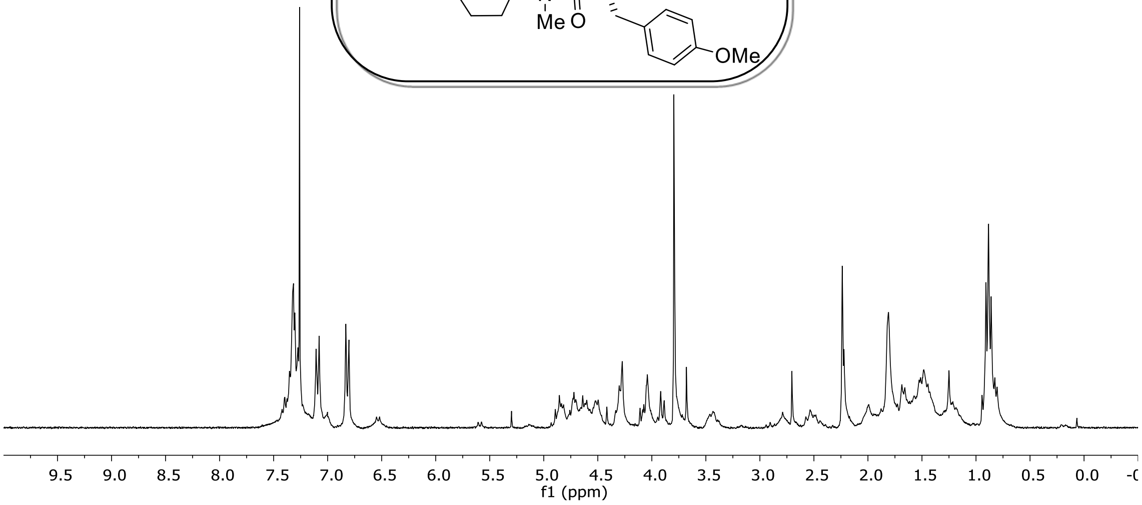

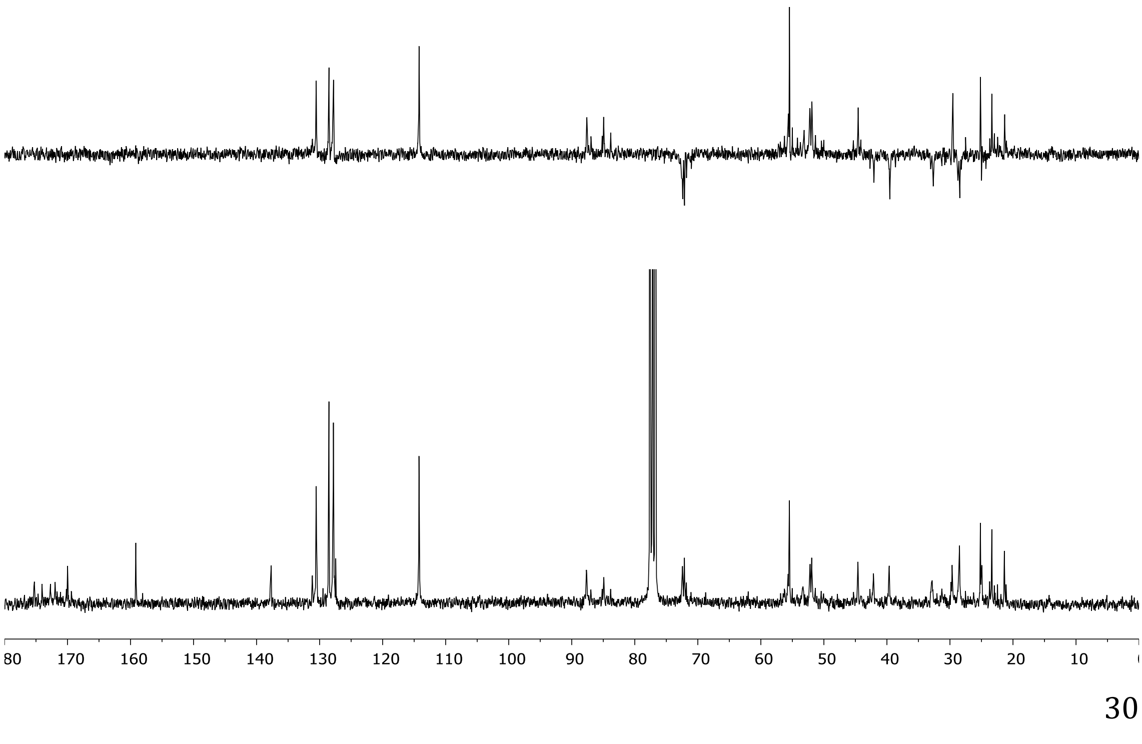

FTIR (298 K, $CHCl_3$):

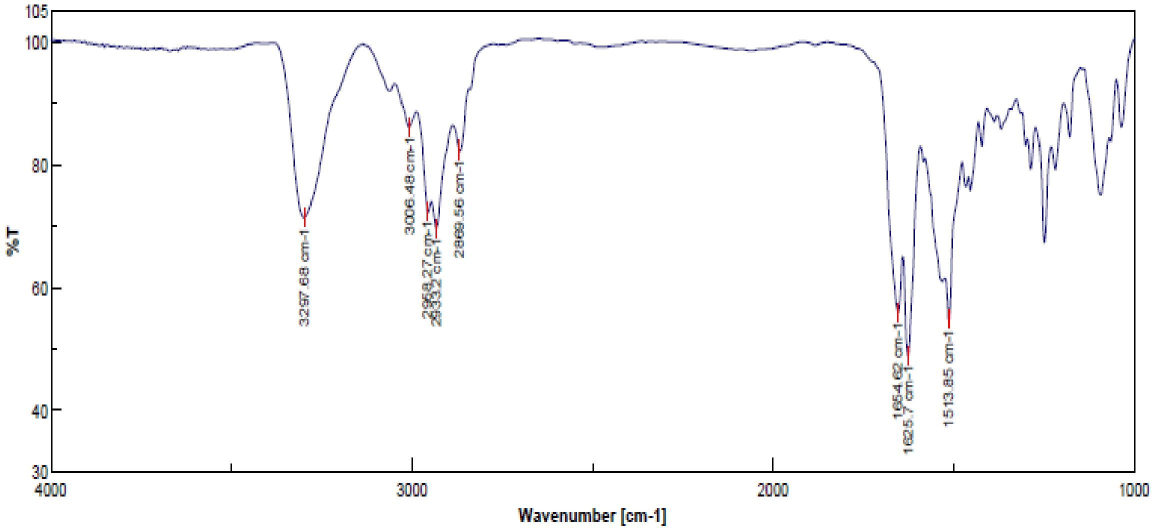

***c*-{[*L*-Ahf-*D*-Tyr(Me)-*L*-^{Me}*N*-Ach-*D*-Leu-]$_2$} (CP2): ^{1}H NMR** (20 mM, $CDCl_3$, 298 K, 300 MHz), **DEPT** and **^{13}C NMR** ($CDCl_3$, 298 K, 75.4 MHz).

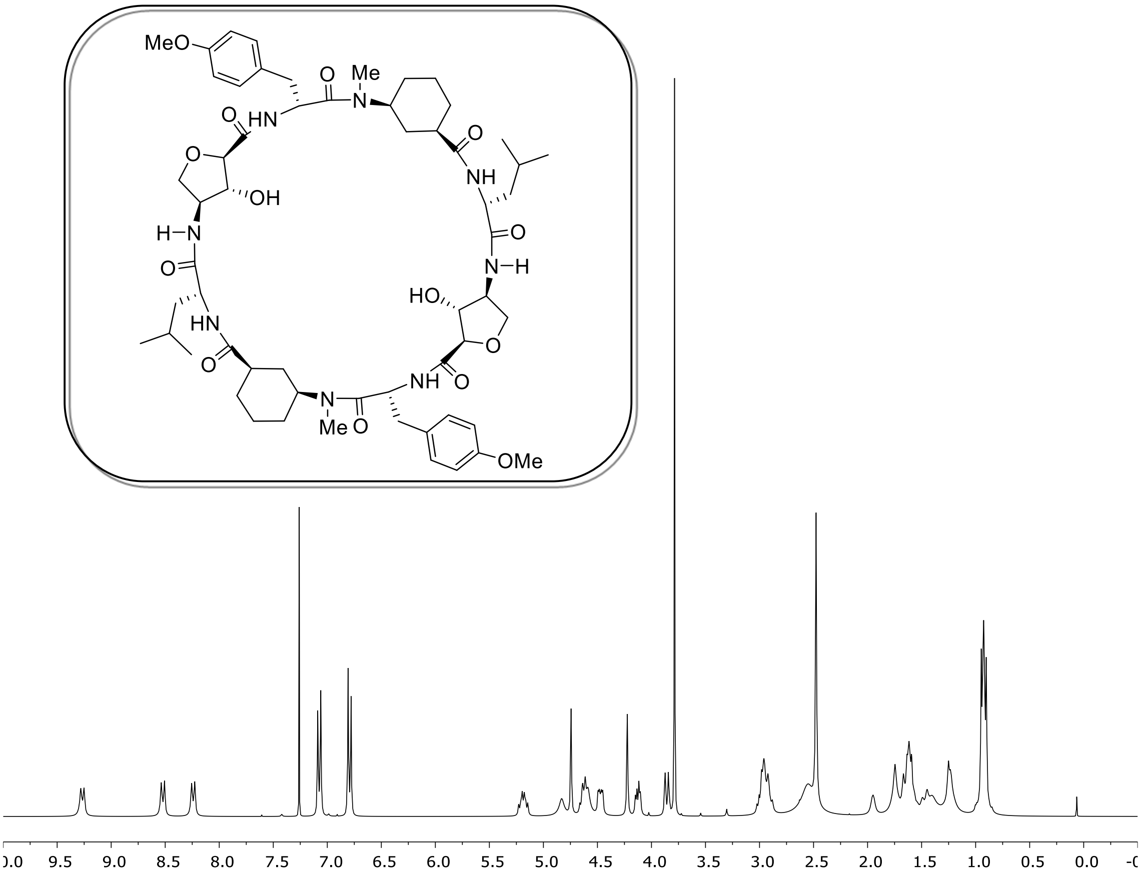

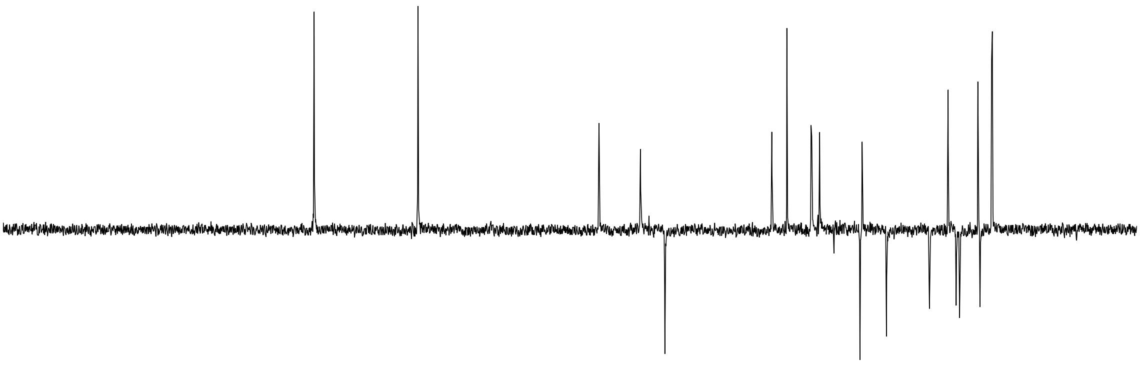

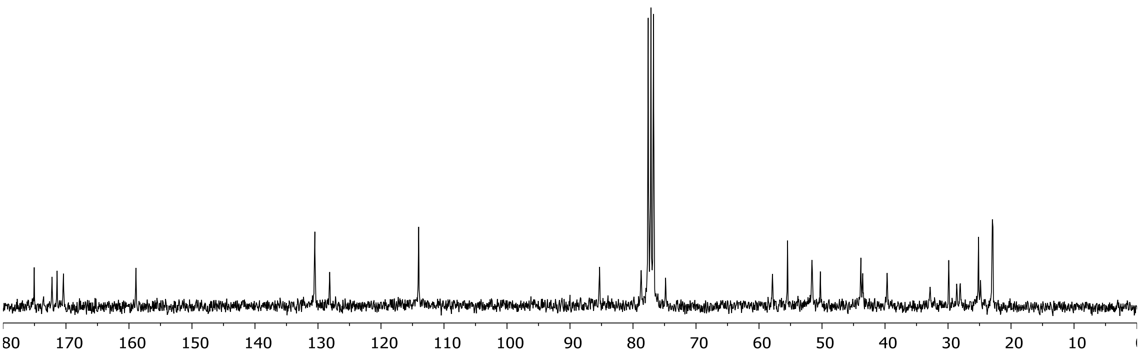

COSY (20 mM, $CDCl_3$, 298 K, 500 MHz).

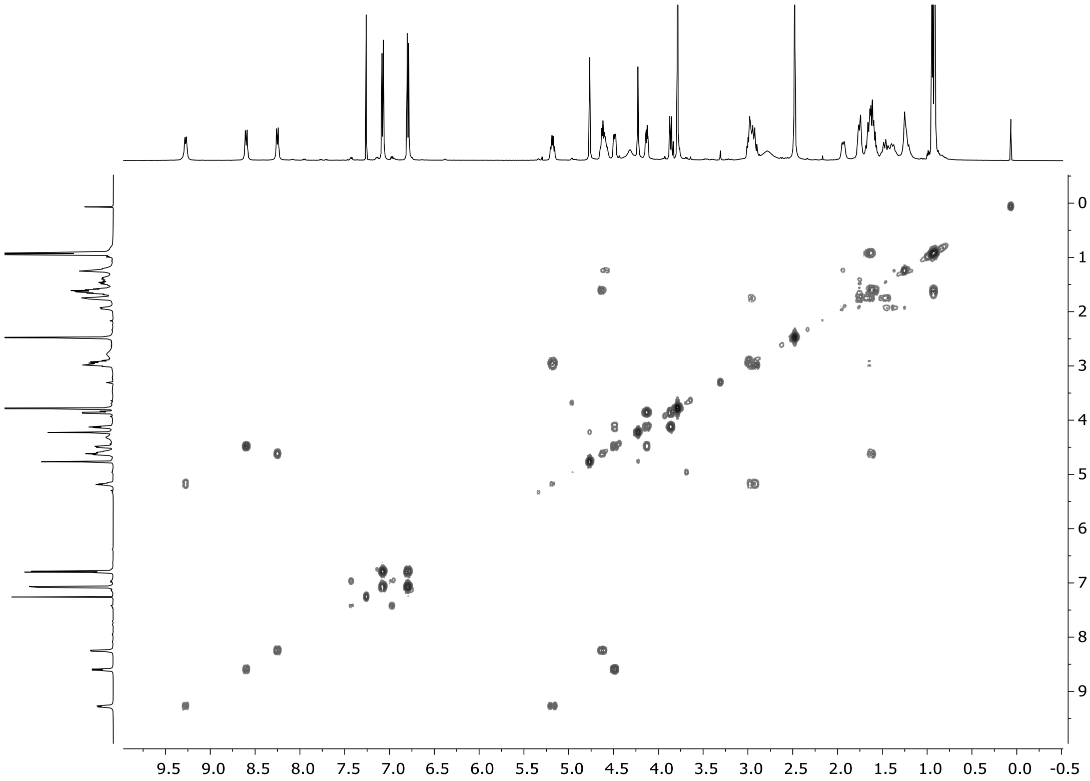

TOCSY (20 mM, $CDCl_3$, 298 K, 500 MHz).

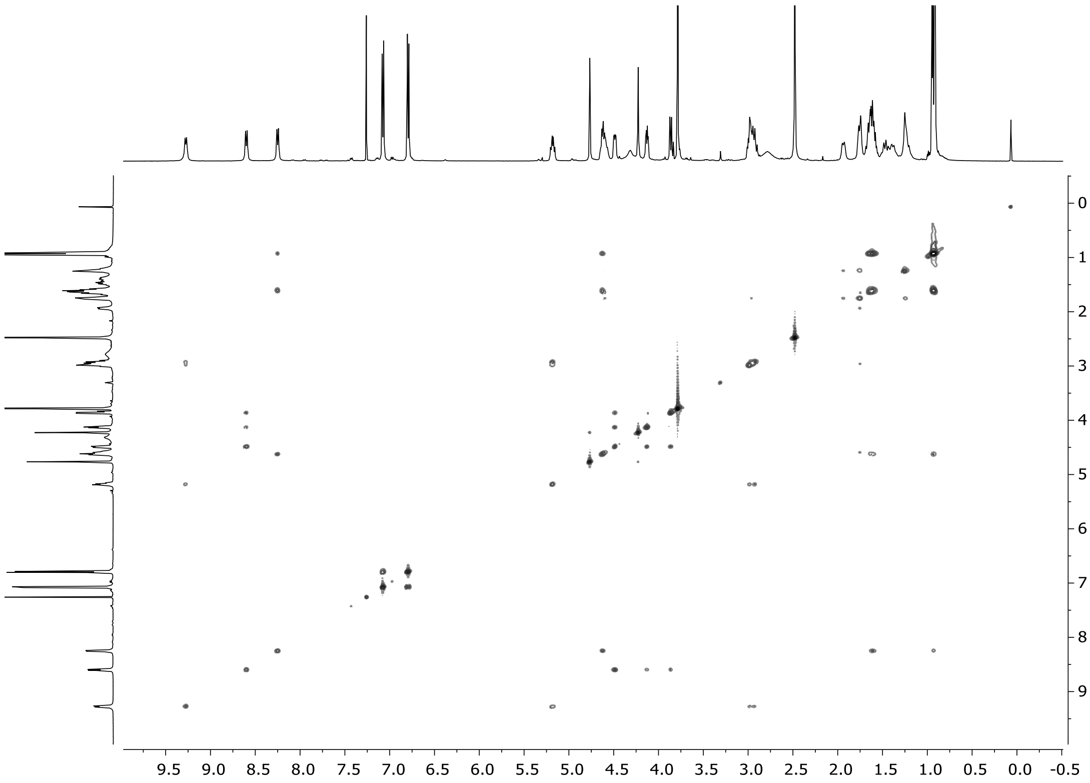

NOESY (20 mM, $CDCl_3$, 298 K, 500 MHz).

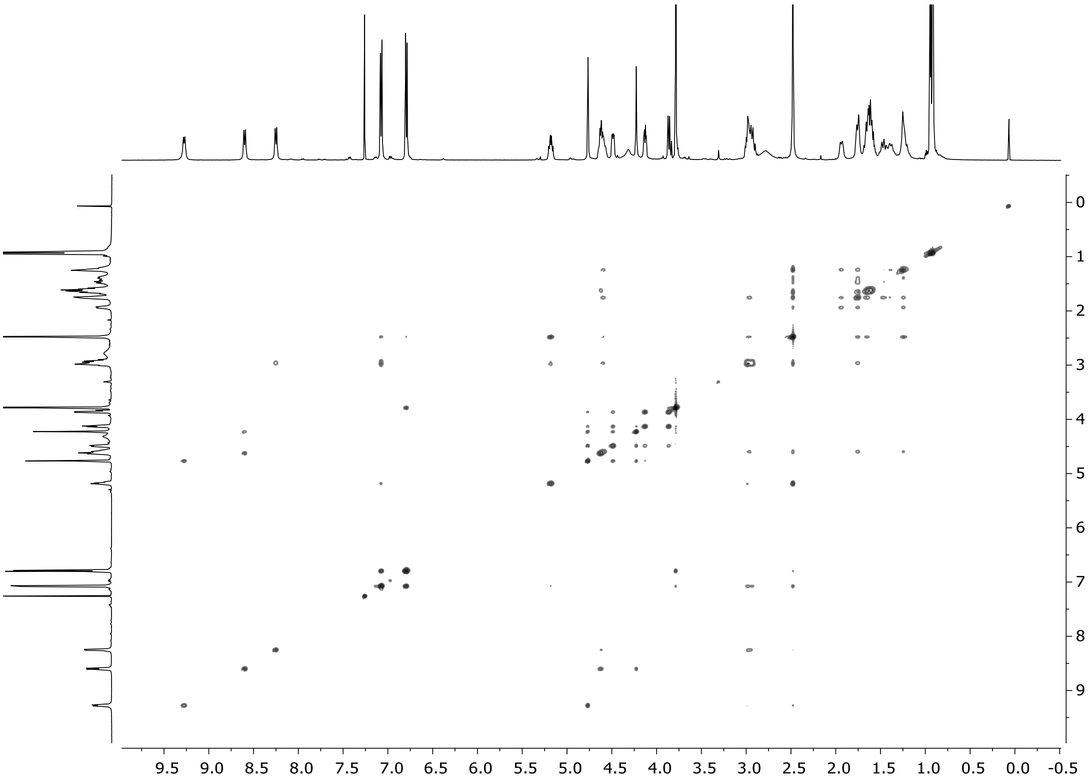

FTIR (298 K, $CHCl_3$):

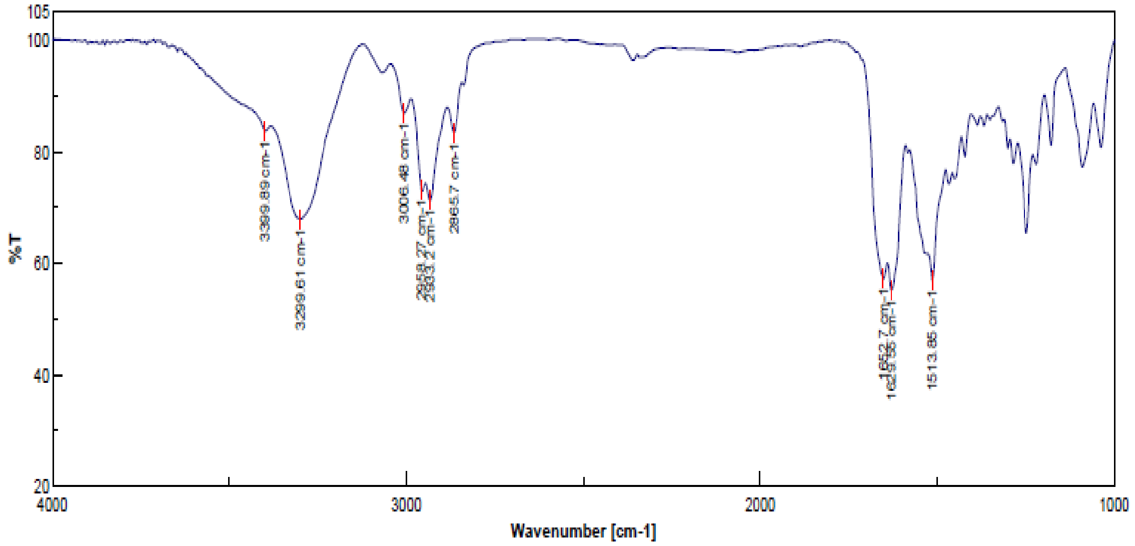

Boc-*D*-Leu-*L*-^{Me}*N*-Ahf(Bn)-OMe (4): ^{1}H NMR (CDCl$_3$, 298 K, 300 MHz), **DEPT** and **^{13}C NMR** (CDCl$_3$, 298 K, 75.4 MHz).

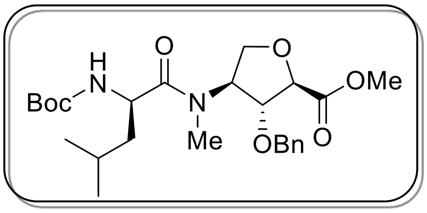

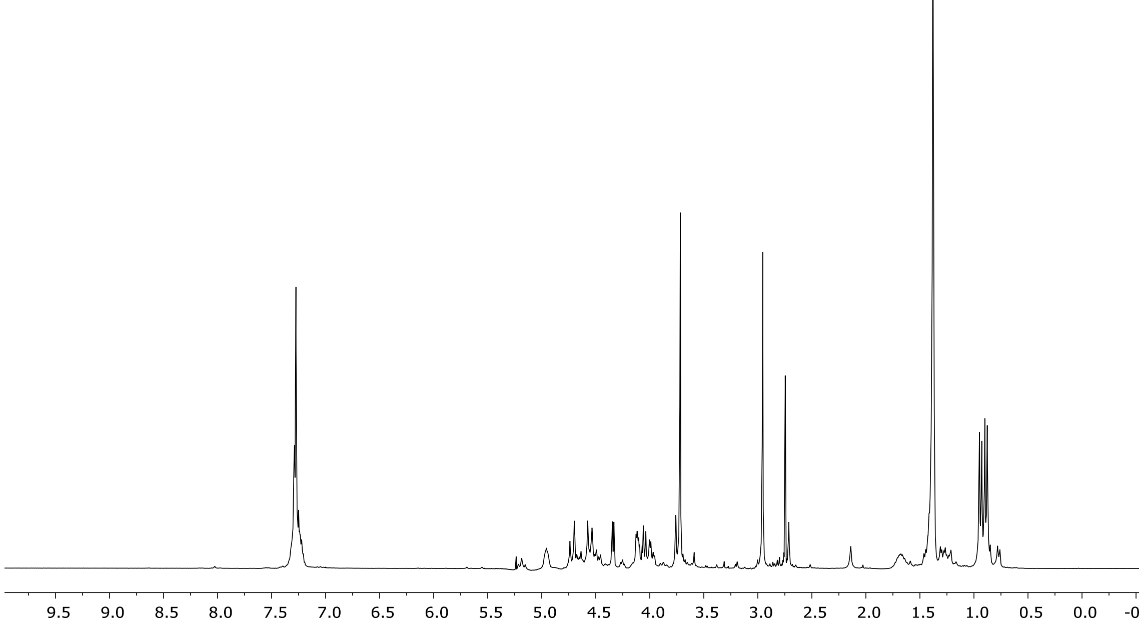

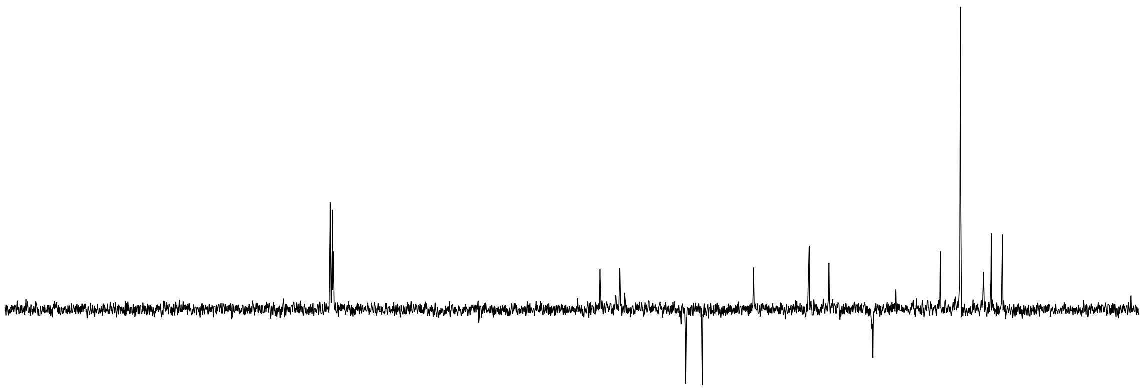

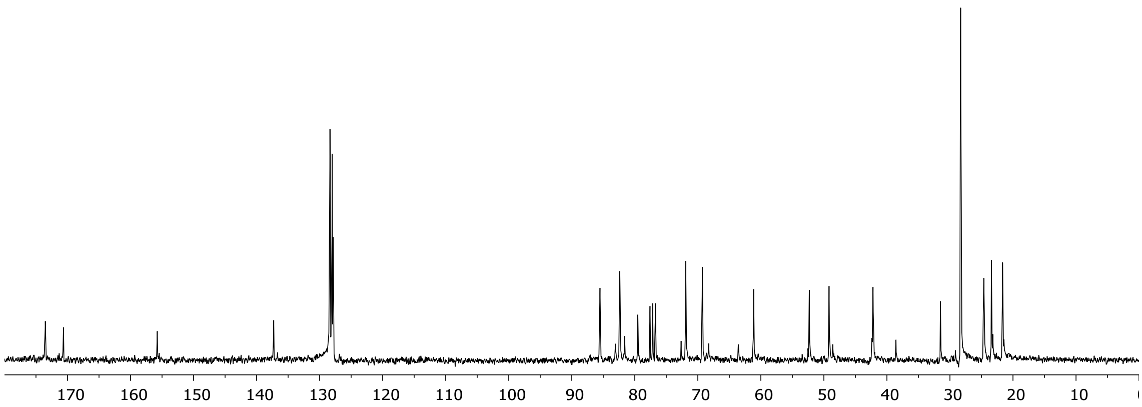

Boc-*D*-Leu-*L*-^{Me}*N*-Ahf(Bn)-OH (dp5): ^{1}**H NMR** ($CDCl_3$, 298 K, 300 MHz), **DEPT** and ^{13}**C NMR** ($CDCl_3$, 298 K, 75.4 MHz).

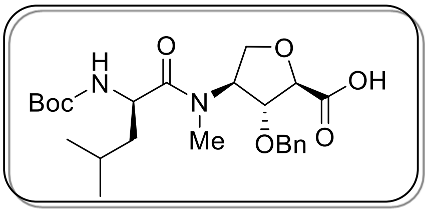

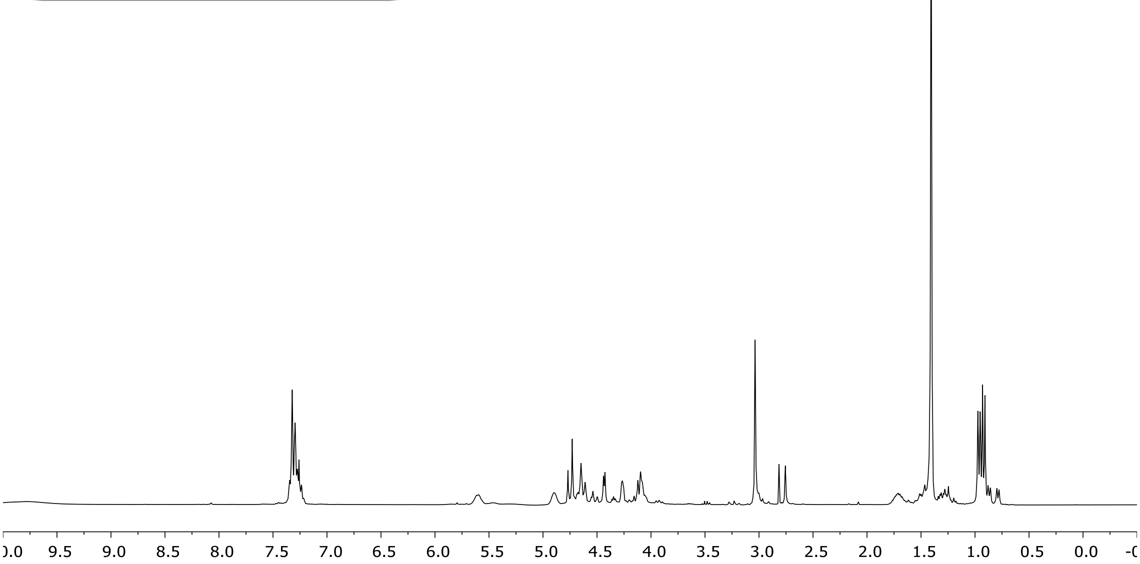

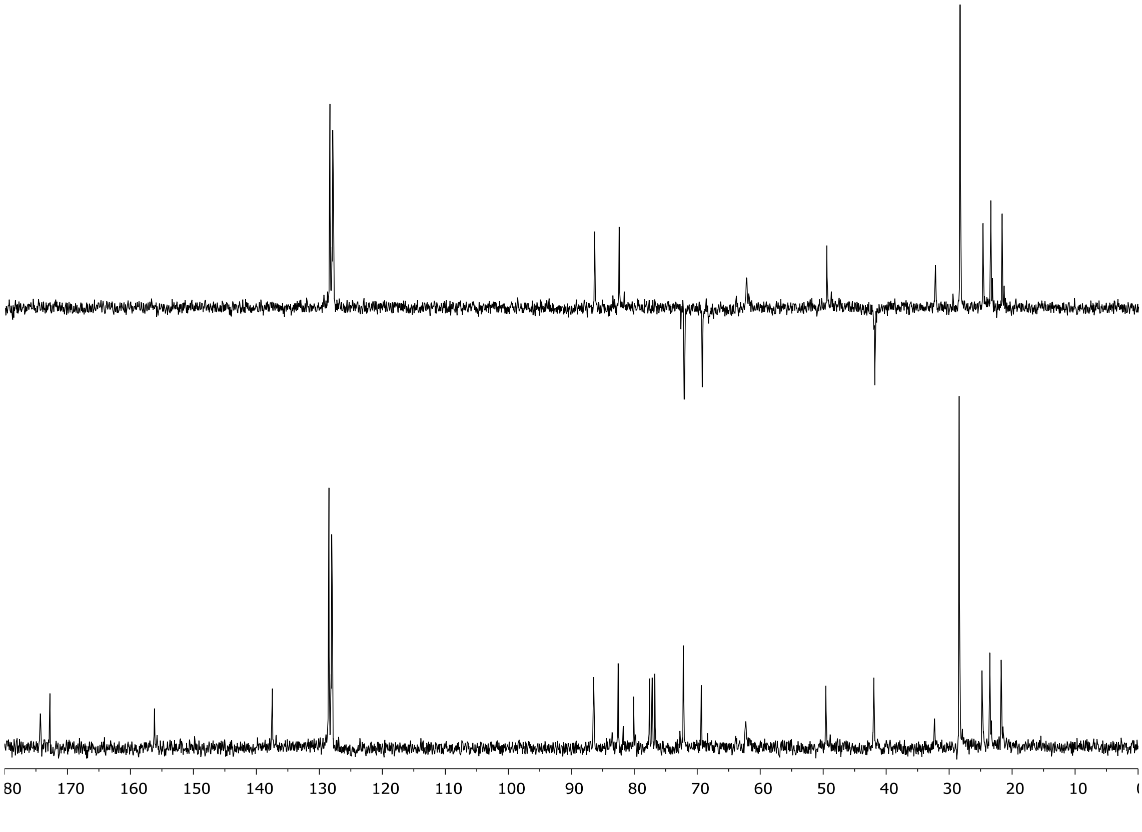

Boc-*D*-Leu-*L*-^{Me}*N*-Ach-OFm (dp4): ^{1}H NMR (CDCl$_3$, 298 K, 300 MHz), **DEPT** and **^{13}C NMR** (CDCl$_3$, 298 K, 75.4 MHz).

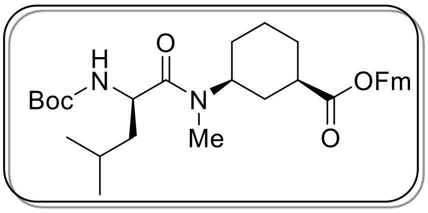

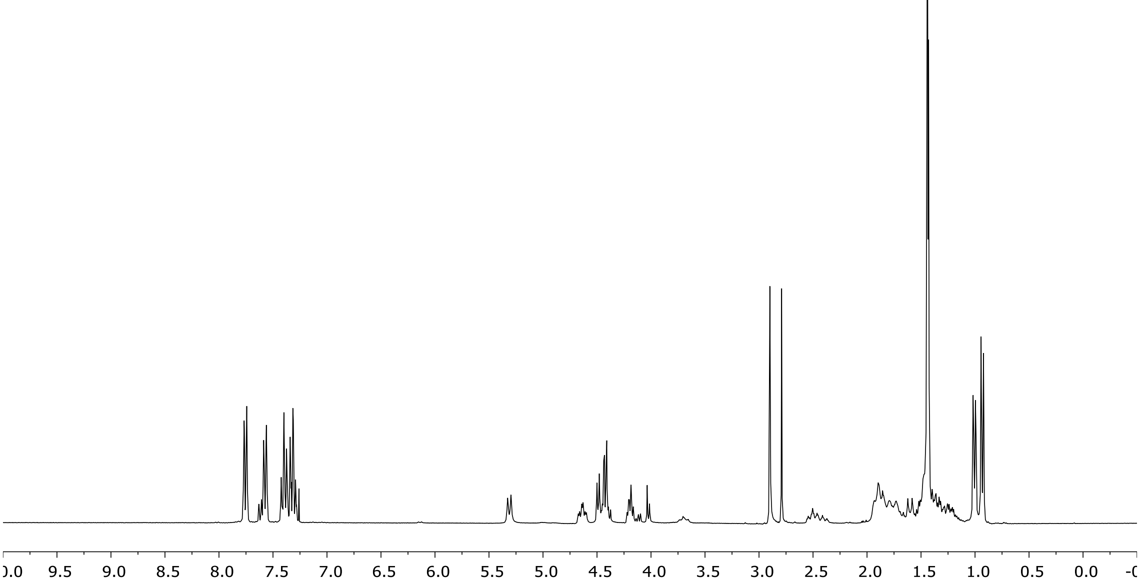

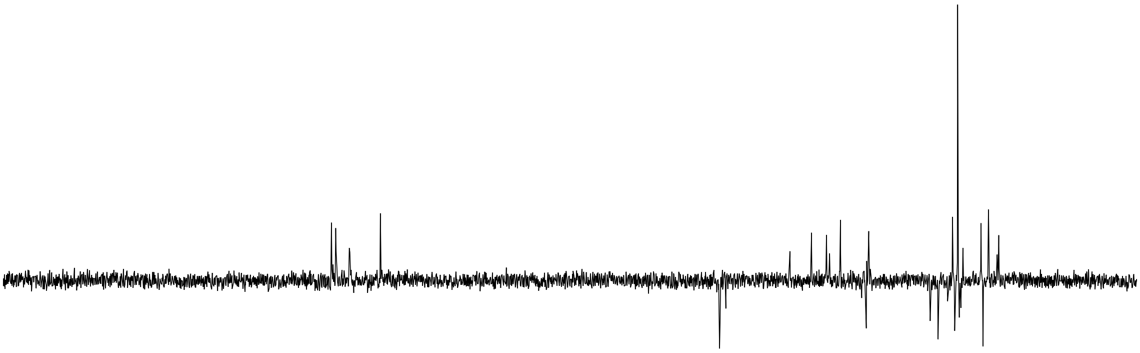

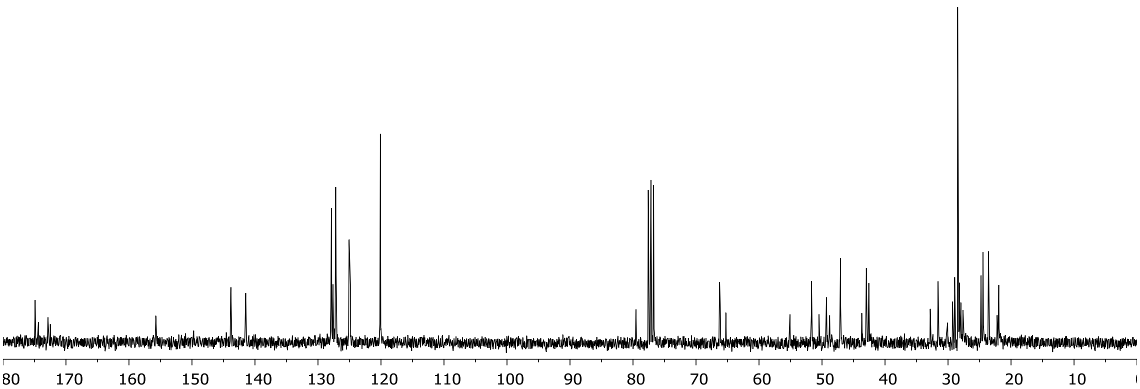

***c*-{[*L*-^{Me}*N*-Ahf(Bn)-*D*-Leu^{1}-*L*-^{Me}*N*-Ach-*D*-Leu^{2}-]$_{2}$} (CP3): ^{1}H NMR** (16 mM, $CDCl_3$, 298 K, 500 MHz), **DEPT** and **^{13}C NMR** ($CDCl_3$, 298 K, 75.4 MHz).

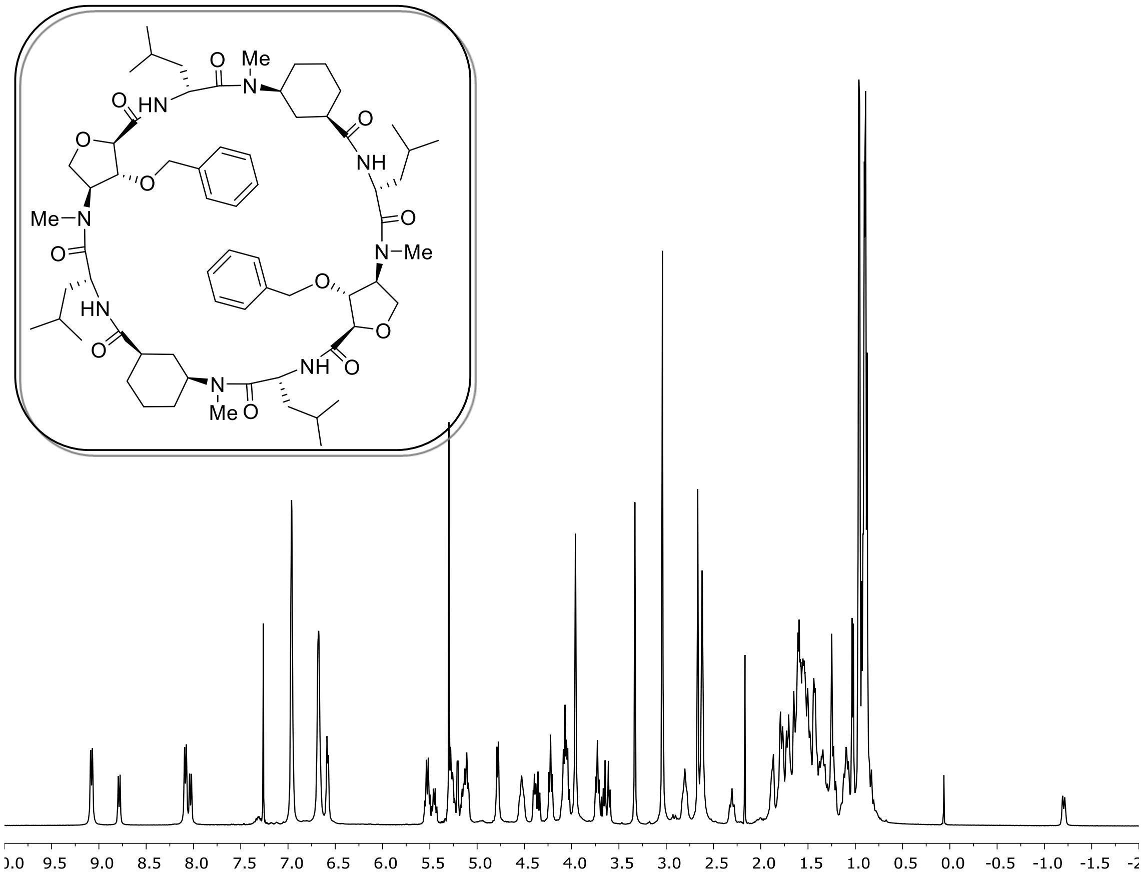

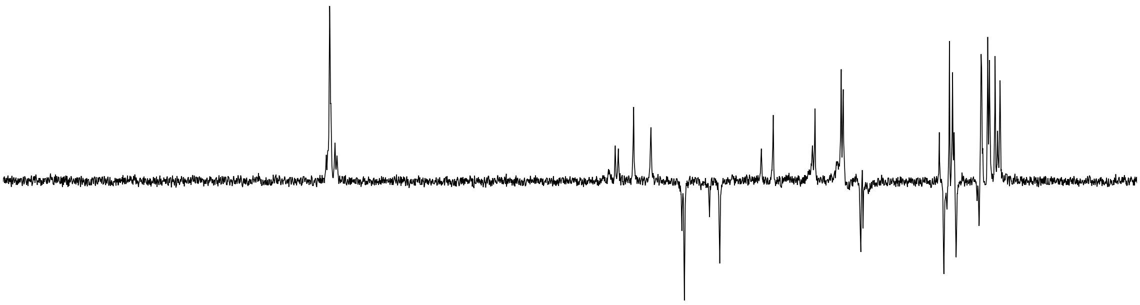

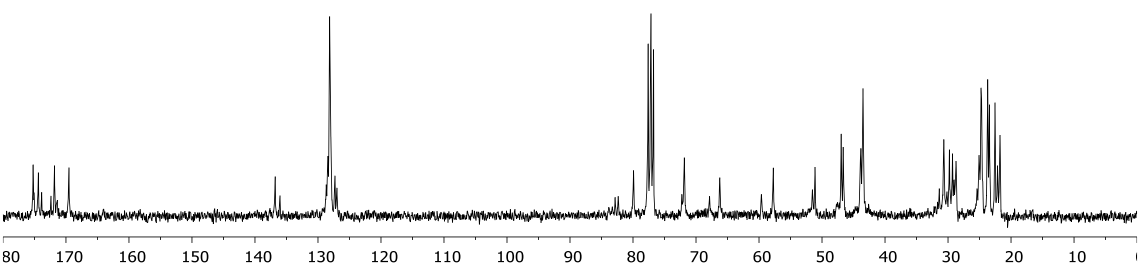

COSY (16 mM, $CDCl_3$, 298 K, 500 MHz).

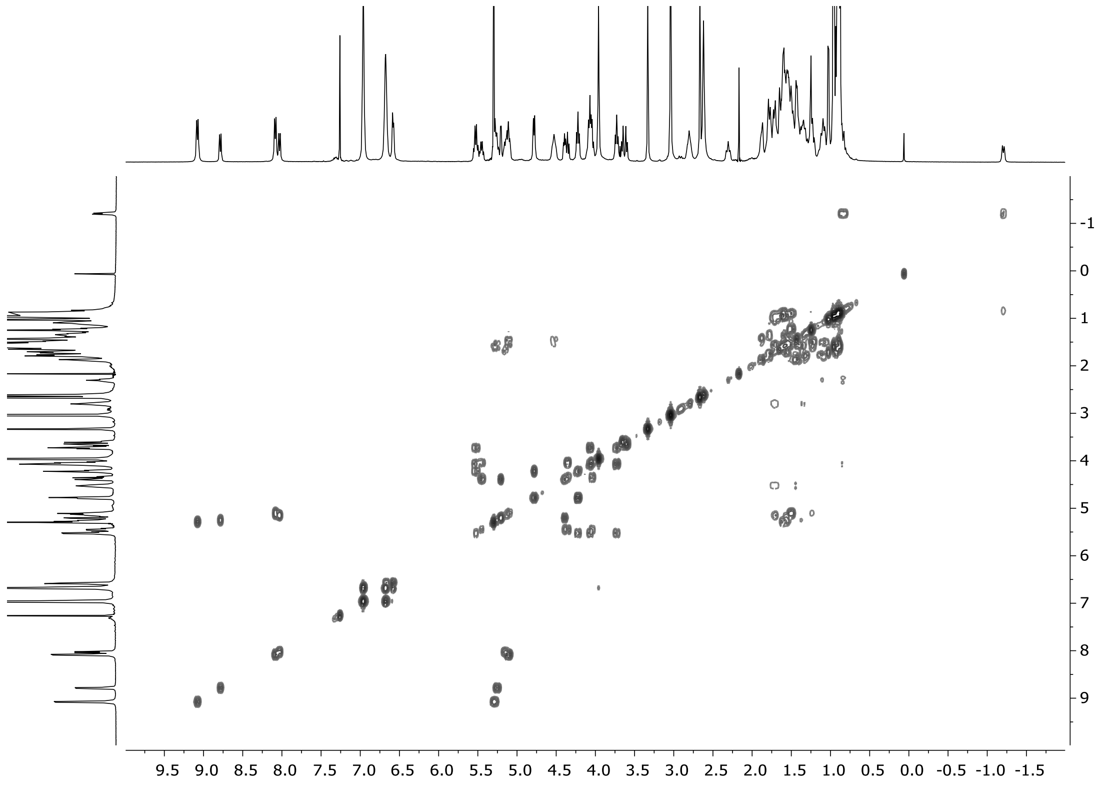

TOCSY (16 mM, $CDCl_3$, 298 K, 500 MHz).

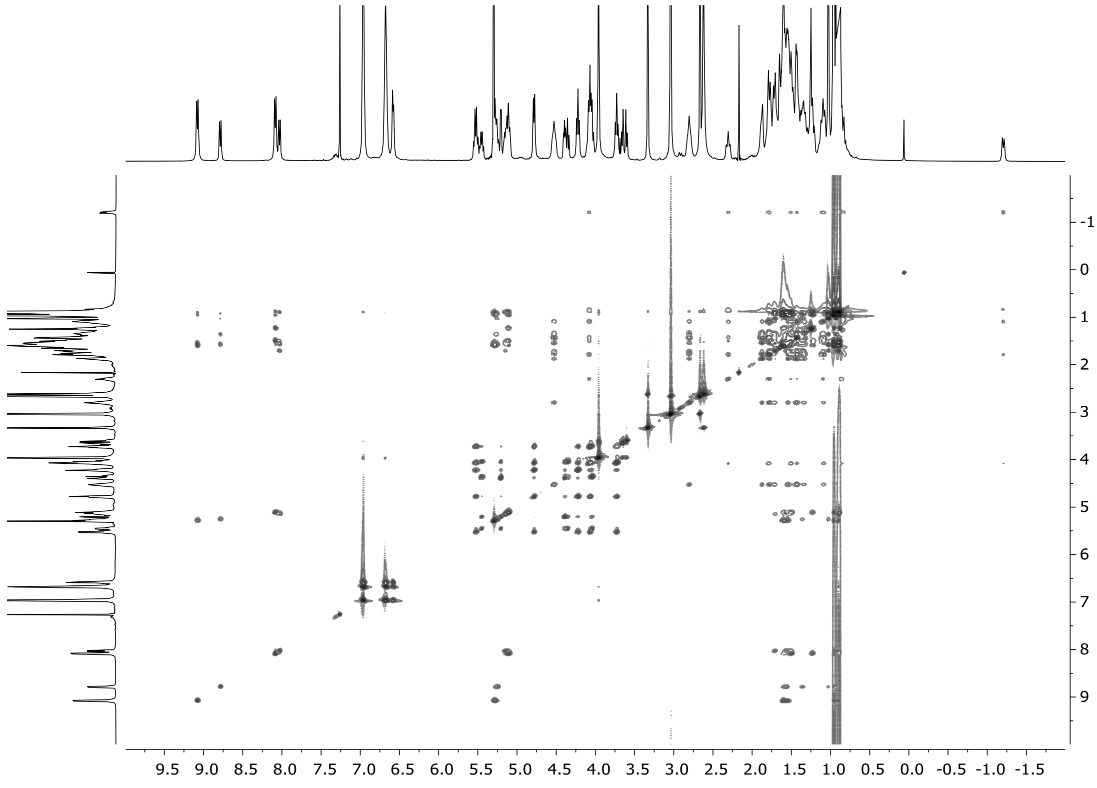

NOESY (16 mM, $CDCl_3$, 298 K, 500 MHz).

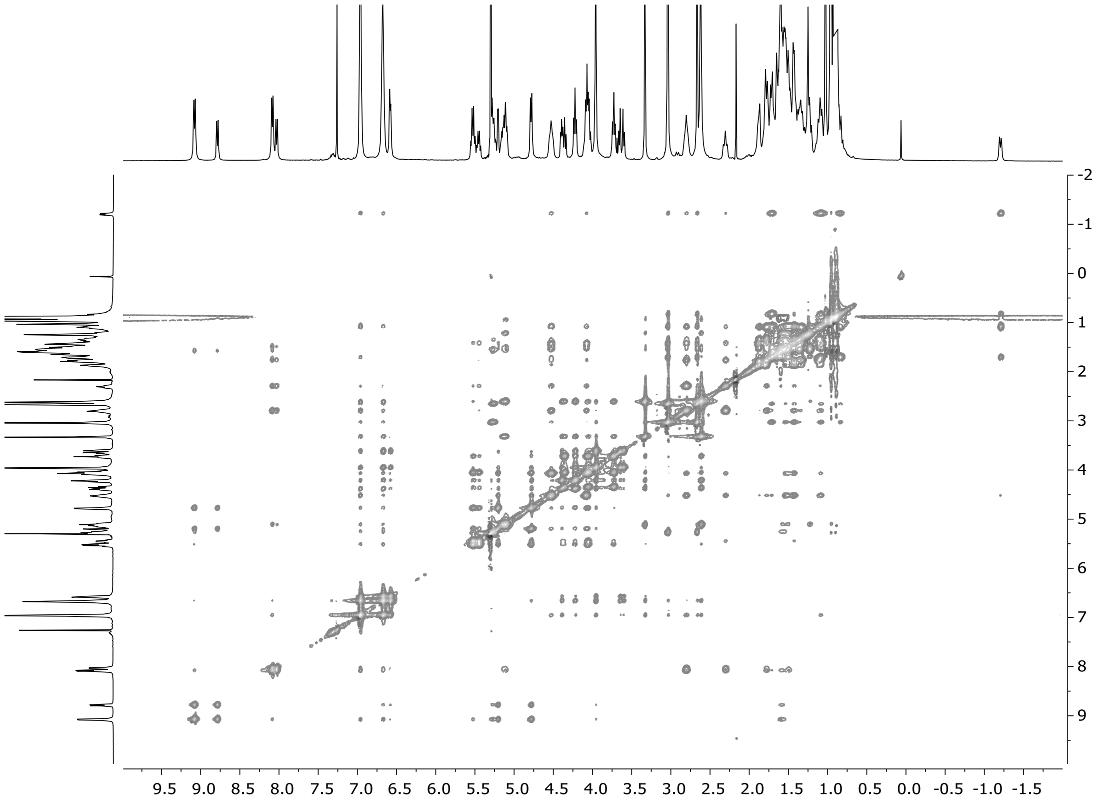

ROESY (16 mM, $CDCl_3$, 298 K, 500 MHz).

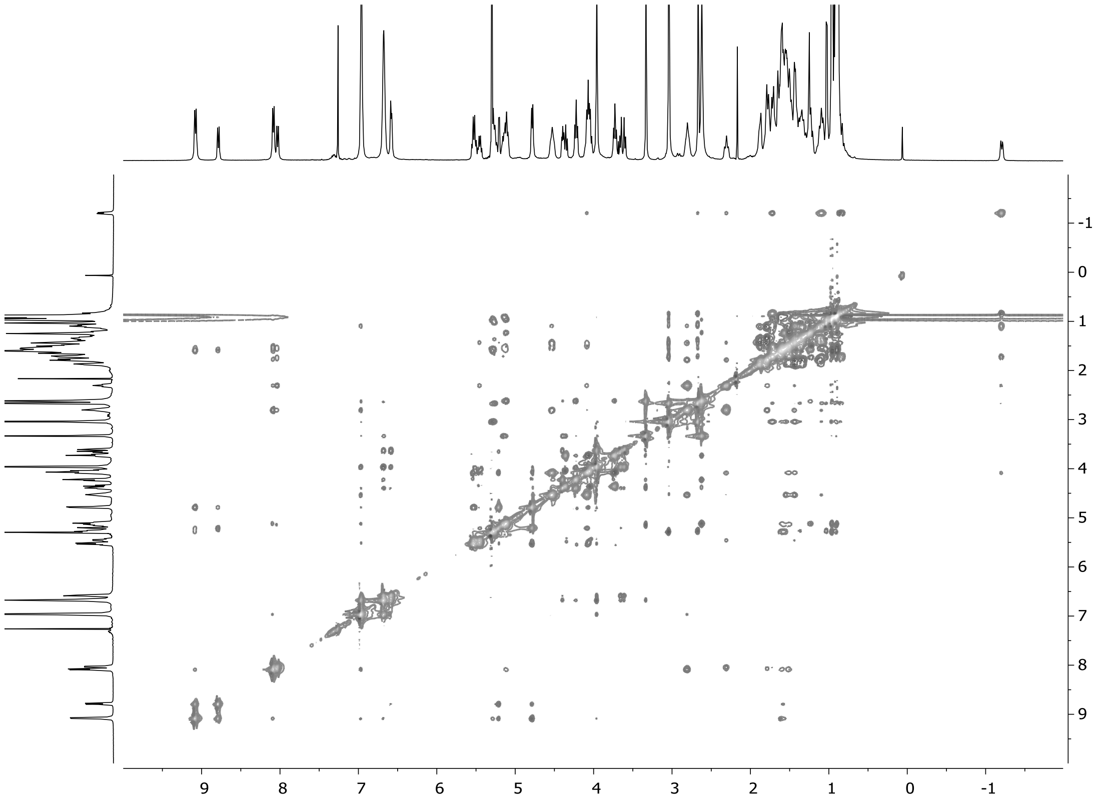

FTIR (298 K, $CHCl_3$):

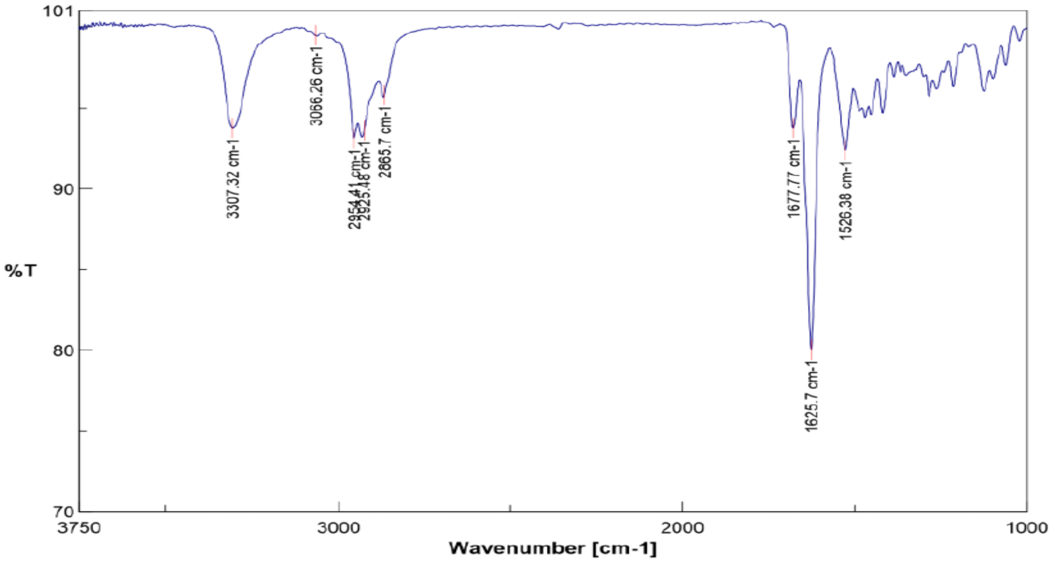

***c*-{[*L*-^{Me}*N*-Ahf-*D*-Leu^{1}-*L*-^{Me}*N*-Ach-*D*-Leu^{2}-]$_{2}$} (CP4):**

^{1}H NMR (5 mM, $CDCl_3$, 298 K, 500 MHz).

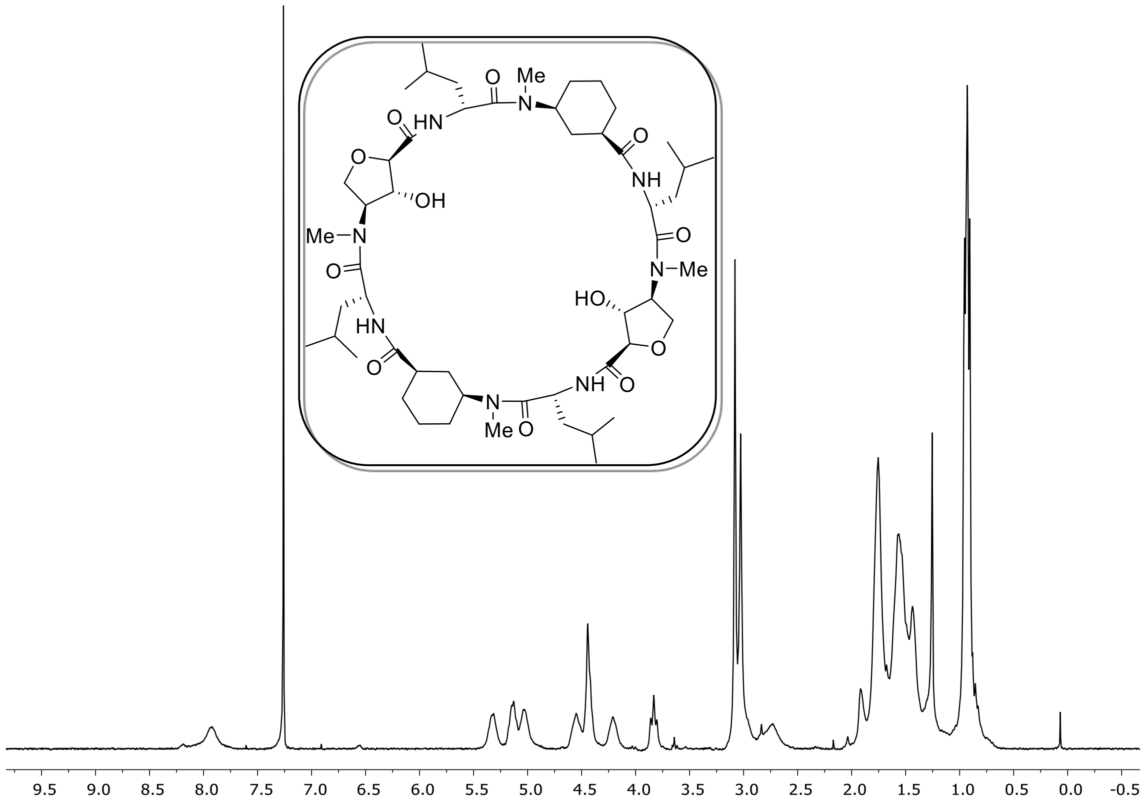

^{1}H NMR (32 mM, $CDCl_3$, 253 K, 500 MHz).

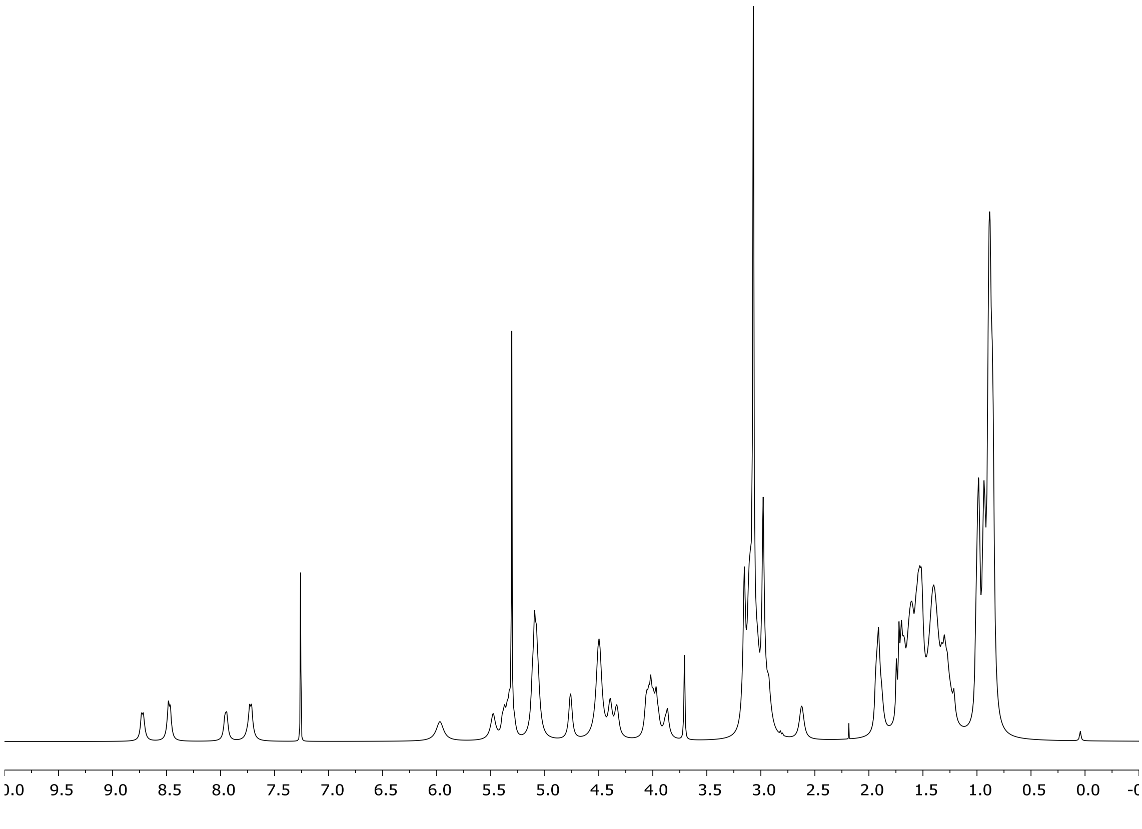

COSY (16 mM, $CDCl_3$, 253 K, 500 MHz).

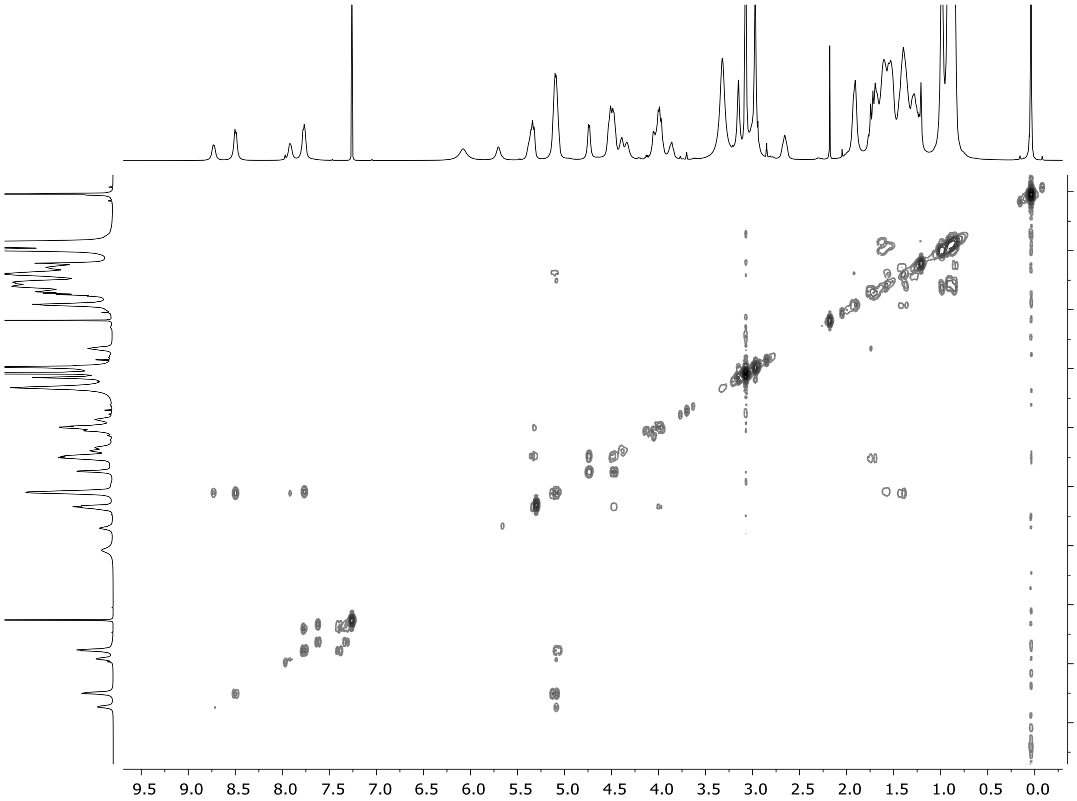

TOCSY (16 mM, $CDCl_3$, 253 K, 500 MHz).

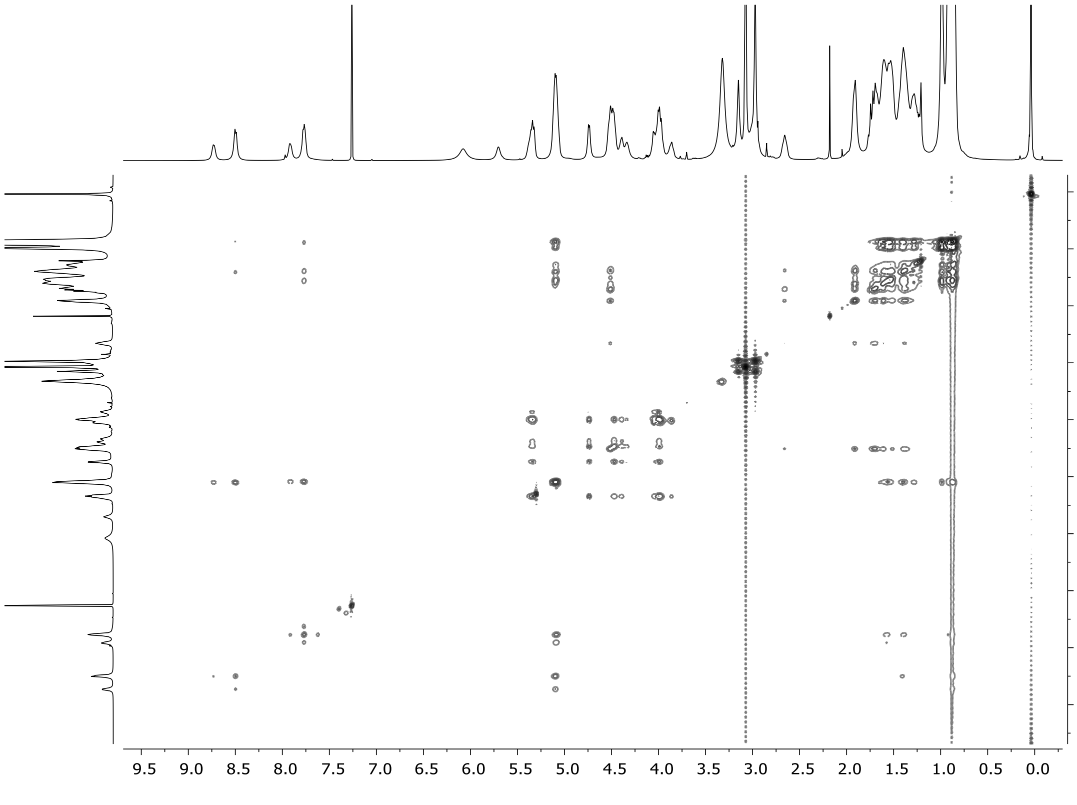

ROESY (16 mM, $CDCl_3$, 253 K, 500 MHz).

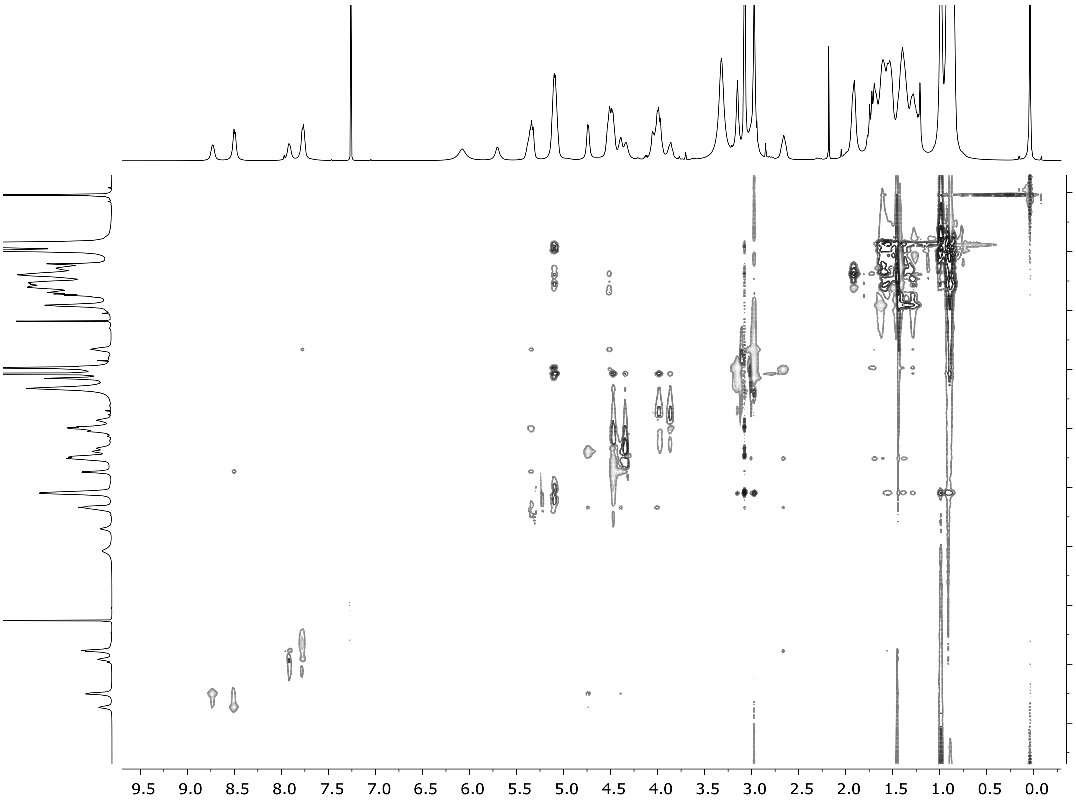

^{1}H NMR (32 mM, 5% $CD_3OH/CDCl_3$, 253 K, 500 MHz).

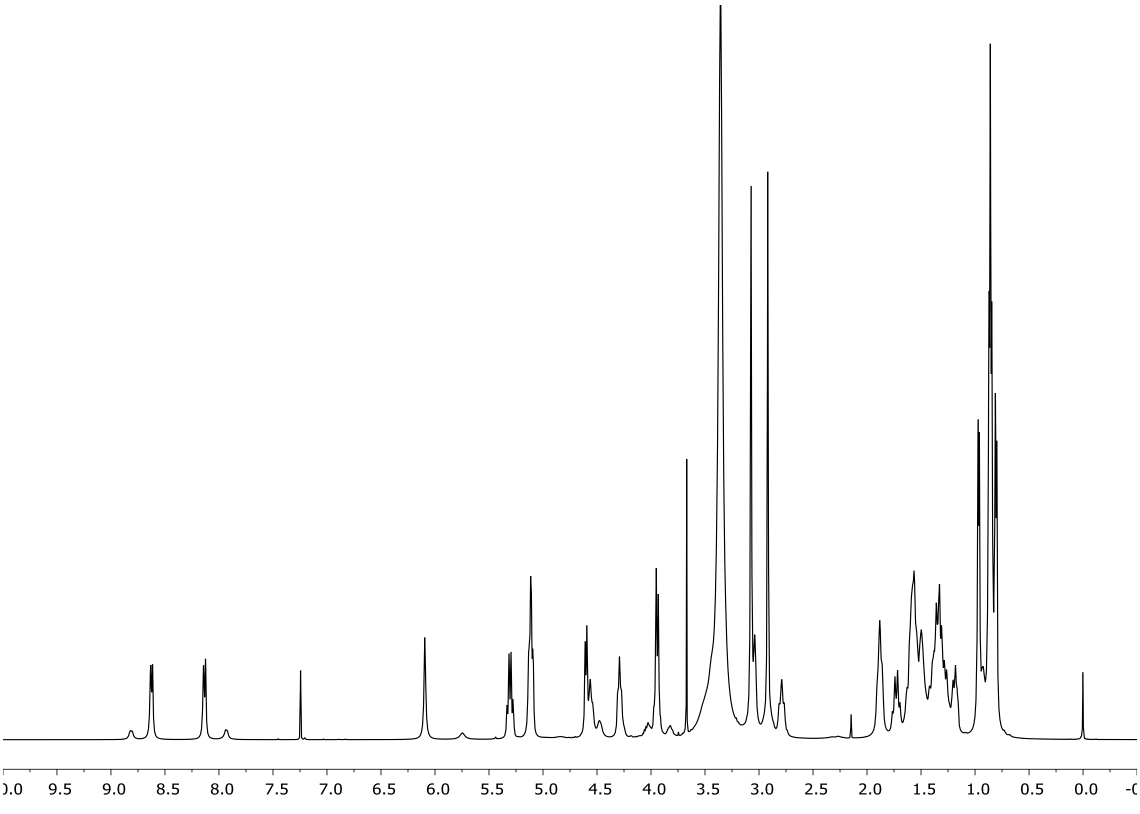

COSY (32 mM, 5% $CD_3OH/CDCl_3$, 253 K, 500 MHz).

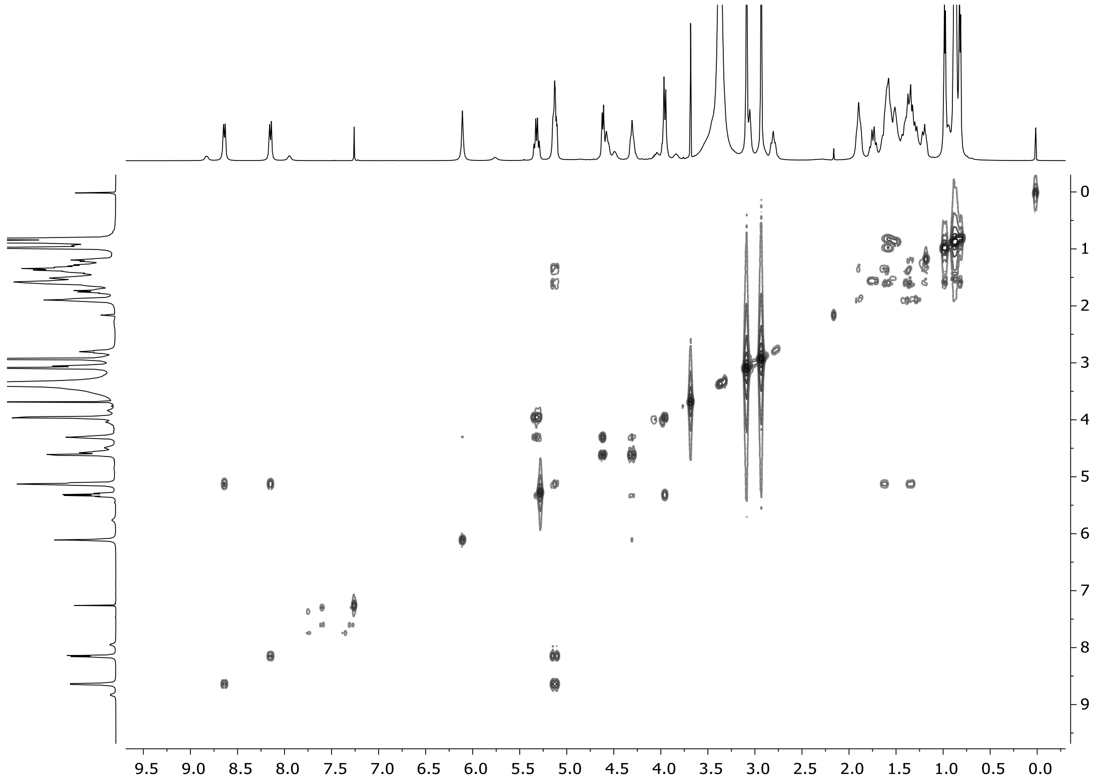

TOCSY (32 mM, 5% $CD_3OH/CDCl_3$, 253 K, 500 MHz).

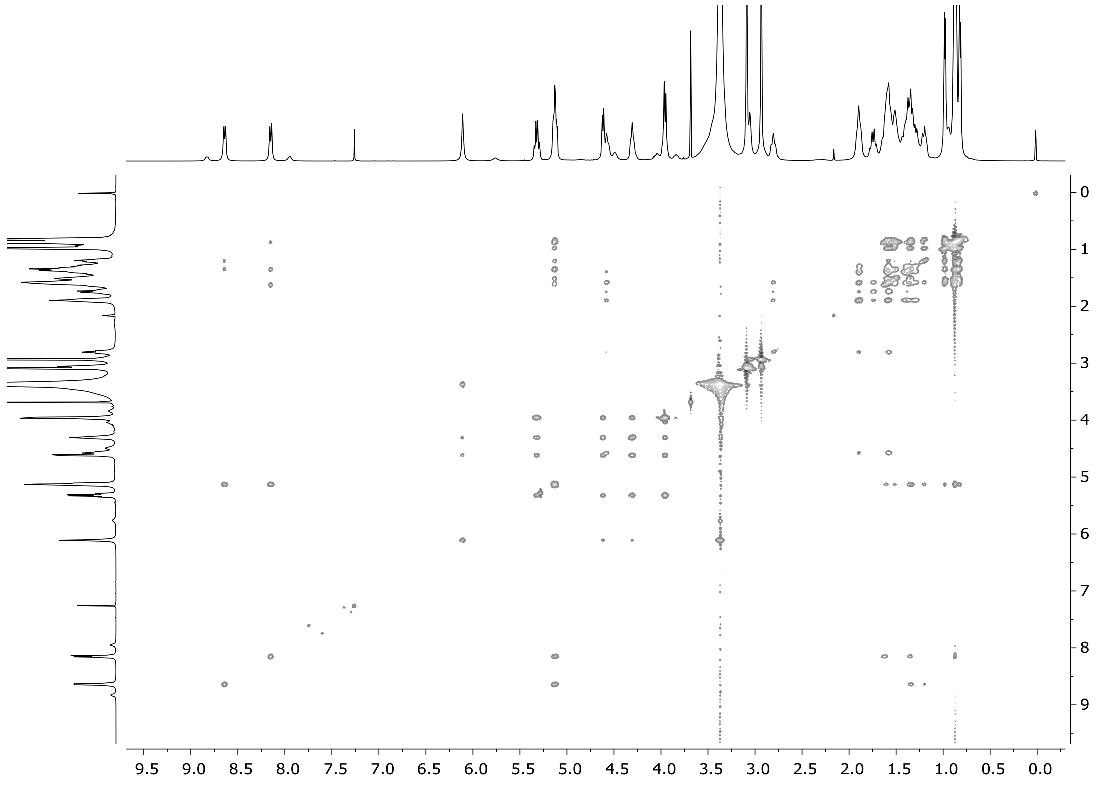

ROESY (32 mM, 5% $CD_3OH/CDCl_3$, 253 K, 500 MHz).

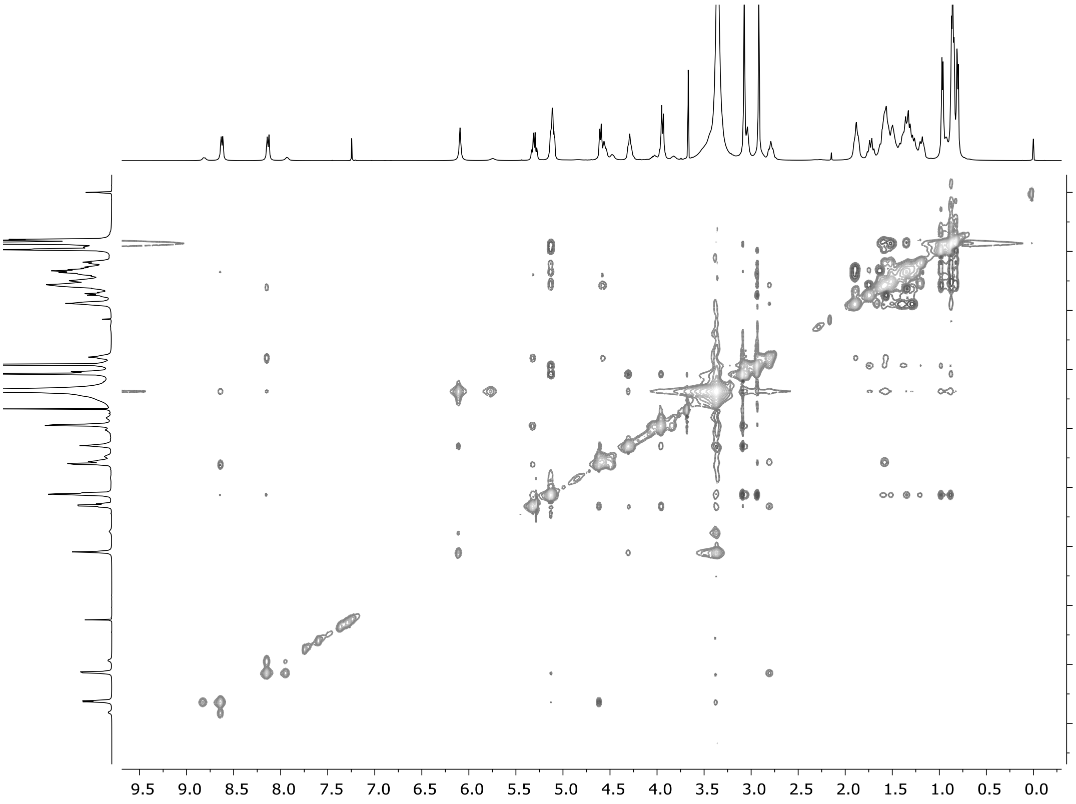

FTIR (298 K, $CHCl_3$):

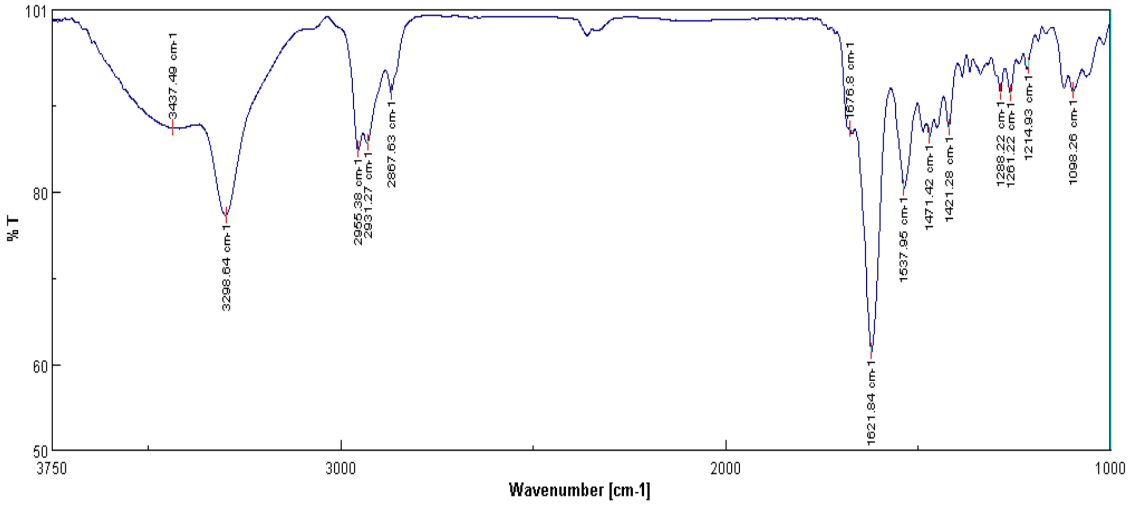

c-[L-^{Me}N-Ahf(pic)-D-Leu^{1}-L-^{Me}N-Ach^{1}-D-Leu^{2}-L-^{Me}N-Ahf-D-Leu^{3}-L-^{Me}N-Ach^{2}-D-Leu^{4}-] (CP5):

^{1}H NMR ($CDCl_3$, 298 K, 300 MHz).

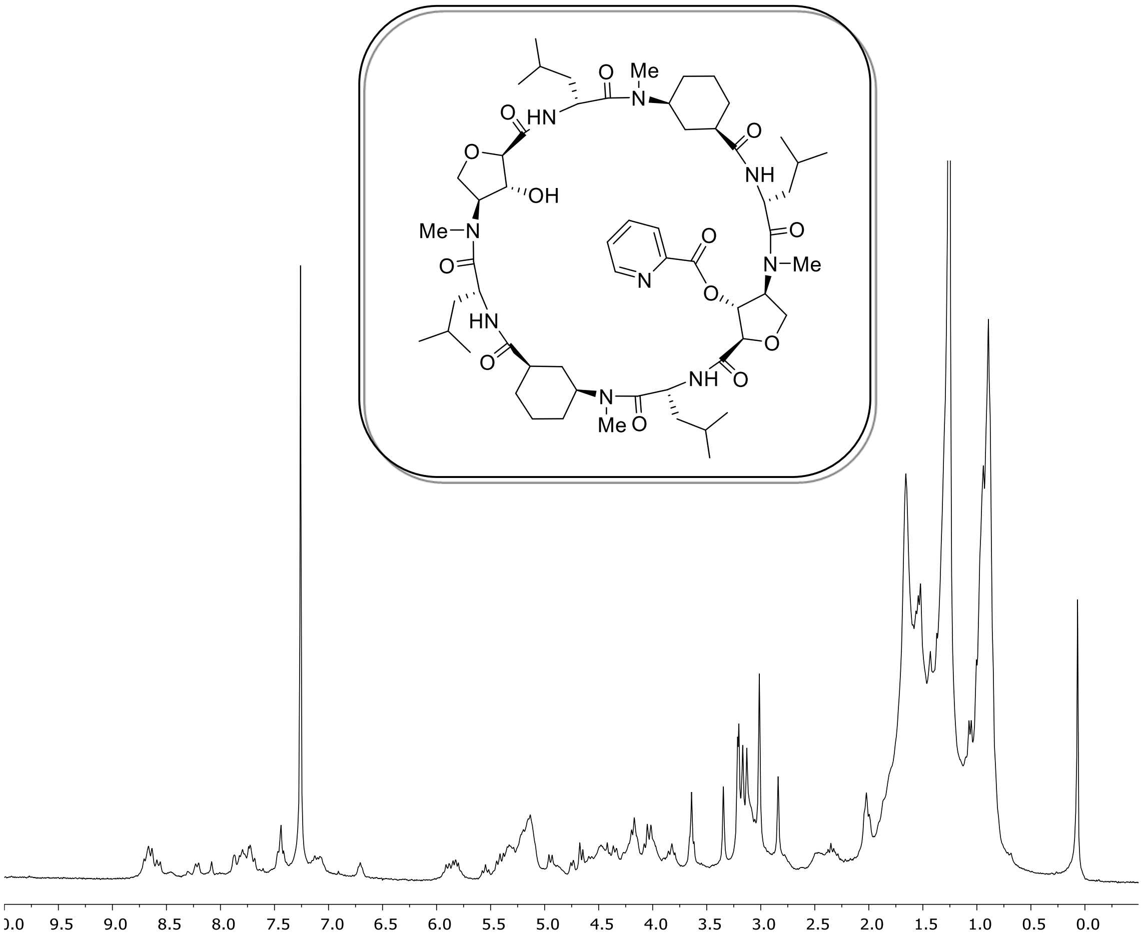

FTIR (298 K, $CHCl_3$):

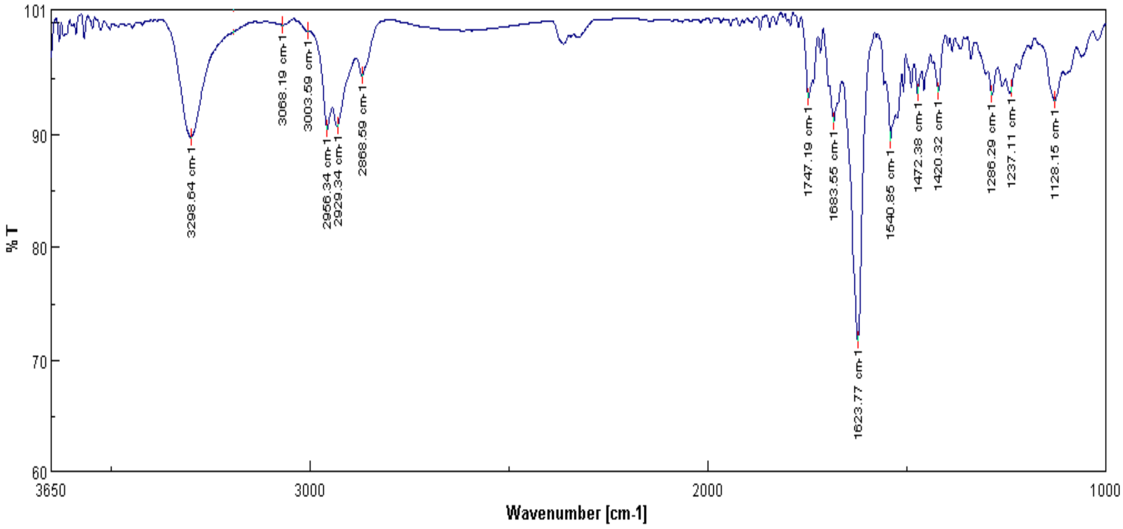

***c*-[*L*-^{Me}*N*-Ahf(pic)-*D*-Leu^{1}-*L*-^{Me}*N*-Ach^{1}-*D*-Leu^{2}-*L*-^{Me}*N*-Ahf-*D*-Leu^{3}-*L*-^{Me}*N*-Ach^{2}-*D*-Leu^{4}-] + 0.5 equiv of $AgBF_4$ (*s*-D5$_E$⊃Ag):** ^{1}H NMR (4.5 mM, $CDCl_3$, 298 K, 500 MHz).

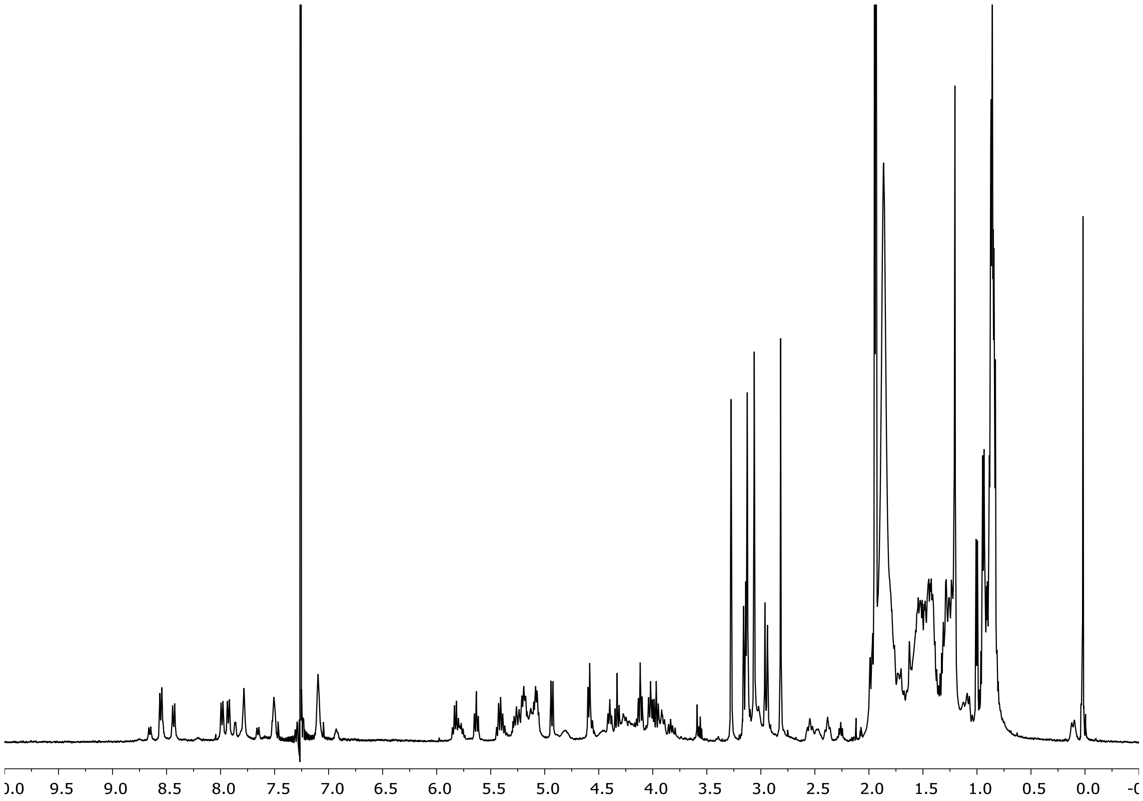

COSY (4.5 mM, $CDCl_3$, 298 K, 500 MHz).

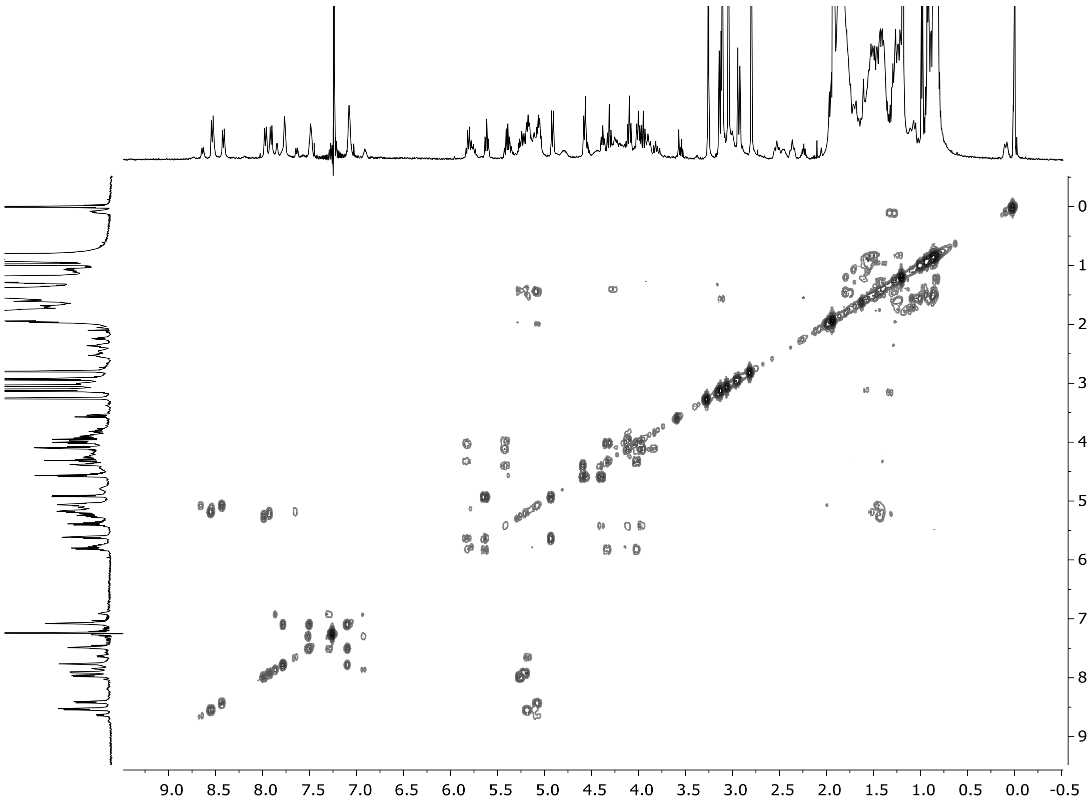

TOCSY (4.5 mM, $CDCl_3$, 298 K, 500 MHz).

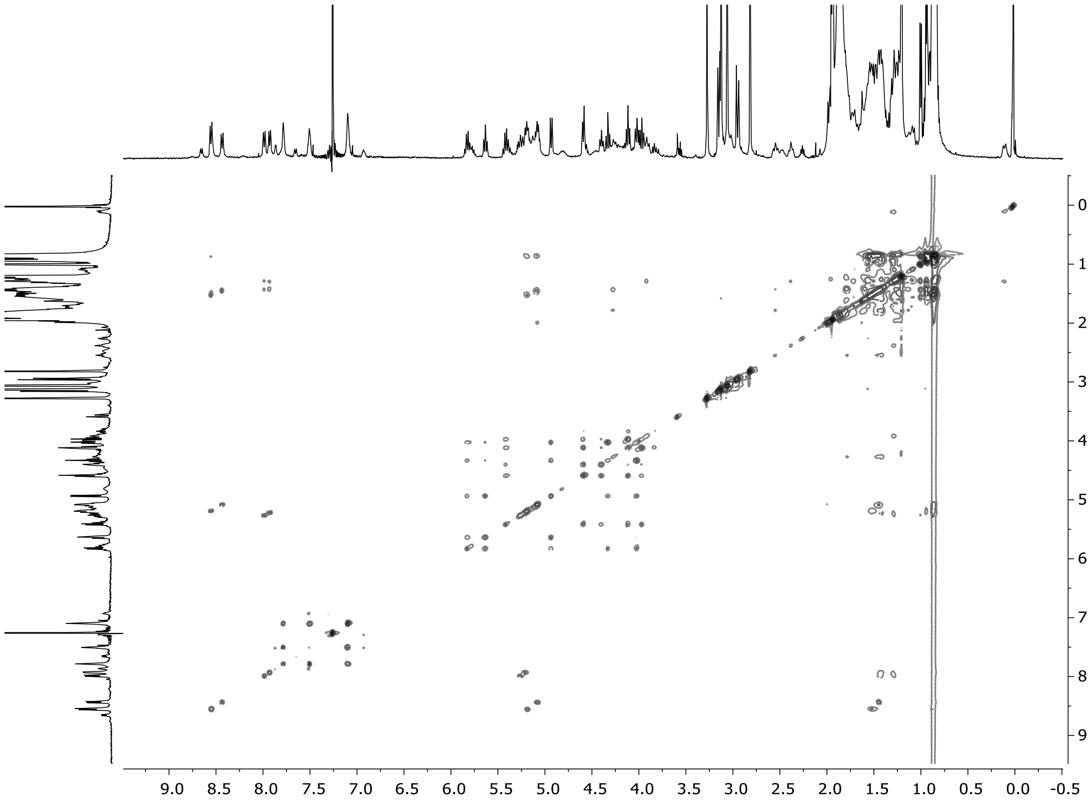

NOESY (4.5 mM, $CDCl_3$, 298 K, 500 MHz).

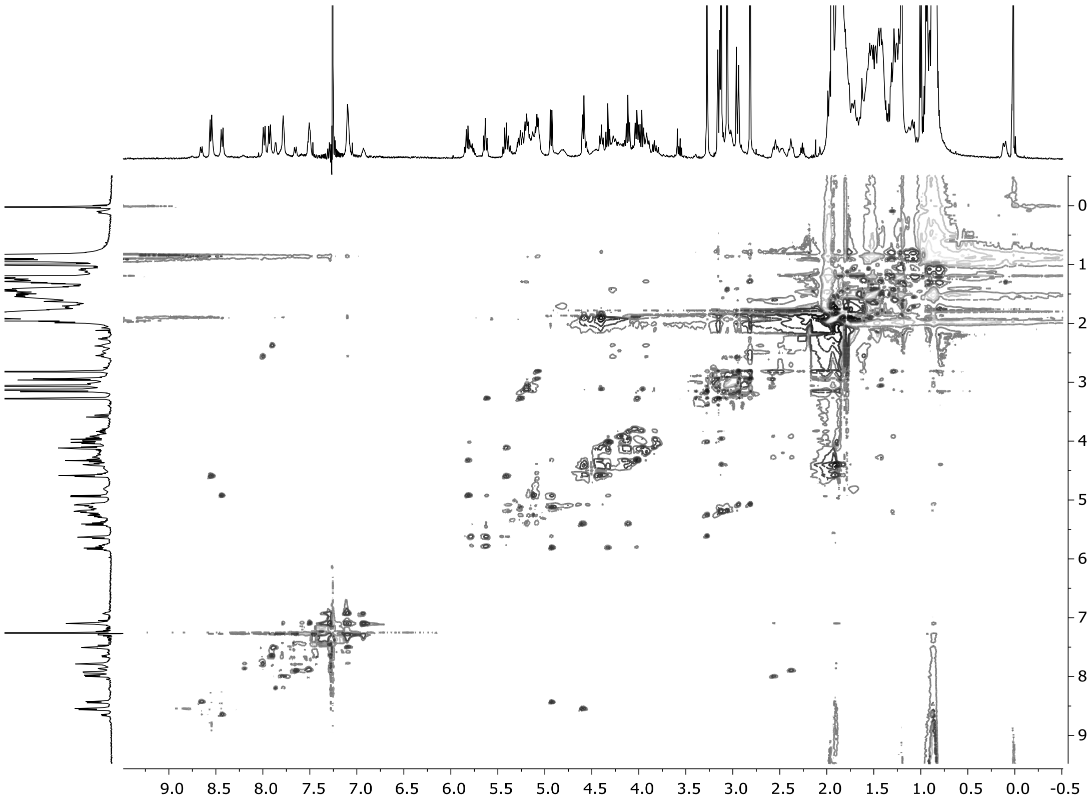

ROESY (4.5 mM, $CDCl_3$, 298 K, 500 MHz).

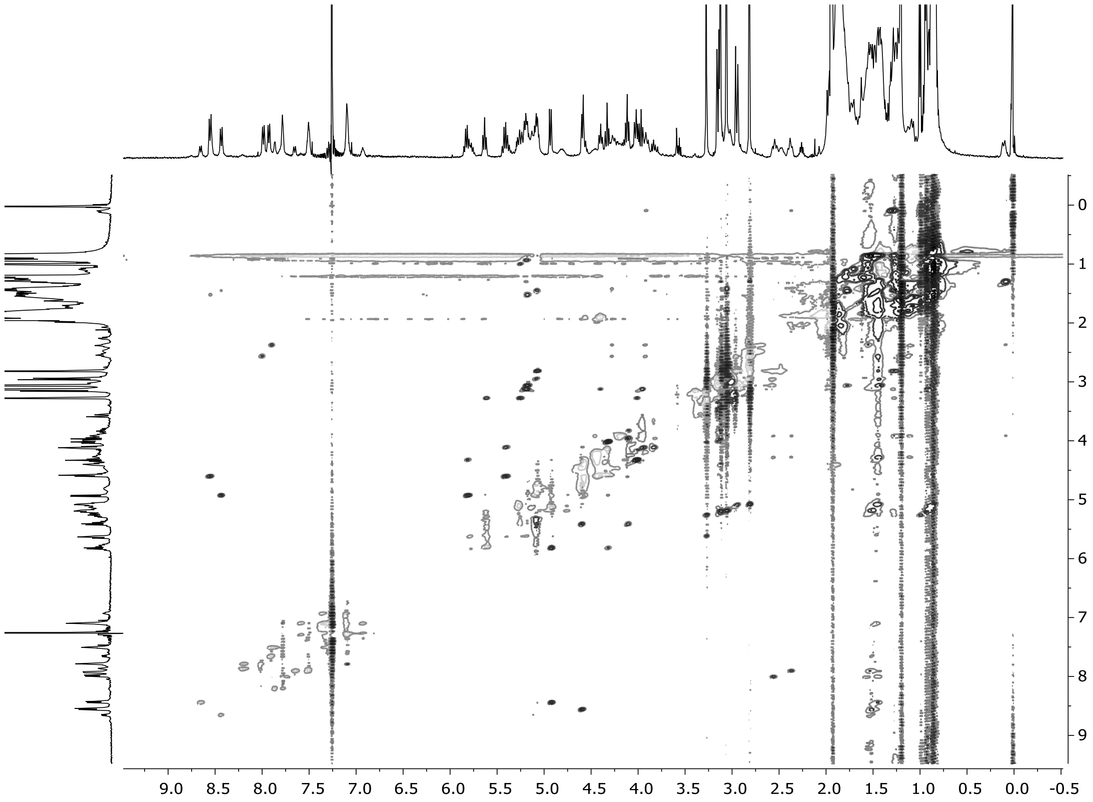

***c*-[*L*-^{Me}*N*-Ahf(pic)-*D*-Leu^{1}-*L*-^{Me}*N*-Ach^{1}-*D*-Leu^{2}-*L*-^{Me}*N*-Ahf-*D*-Leu^{3}-*L*-^{Me}*N*-Ach^{2}-*D*-Leu^{4}-] + 0.5 equiv of $(CO_2H)_2 \cdot 2H_2O$ (*s*-D5$_E$⊃$(CO_2H)_2$):** ^{1}H NMR (5 mM, $CDCl_3$, 298 K, 500 MHz).

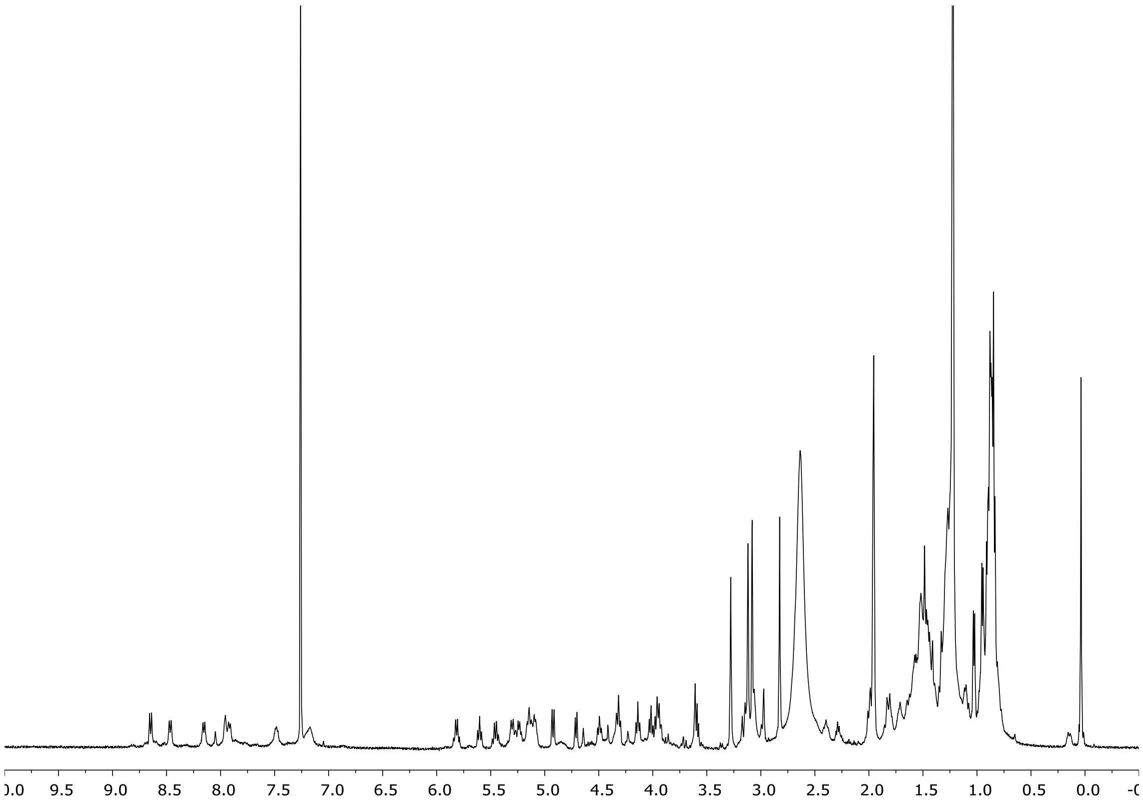

COSY (5 mM, $CDCl_3$, 298 K, 500 MHz).

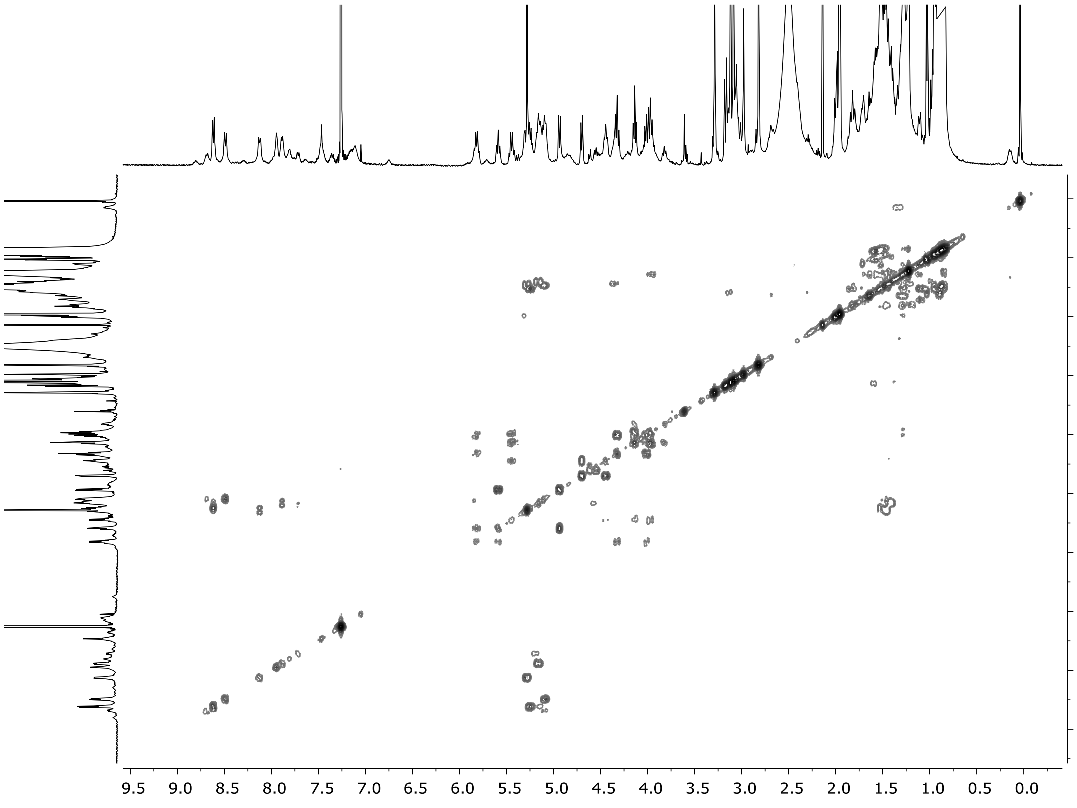

TOCSY (5 mM, $CDCl_3$, 298 K, 500 MHz).

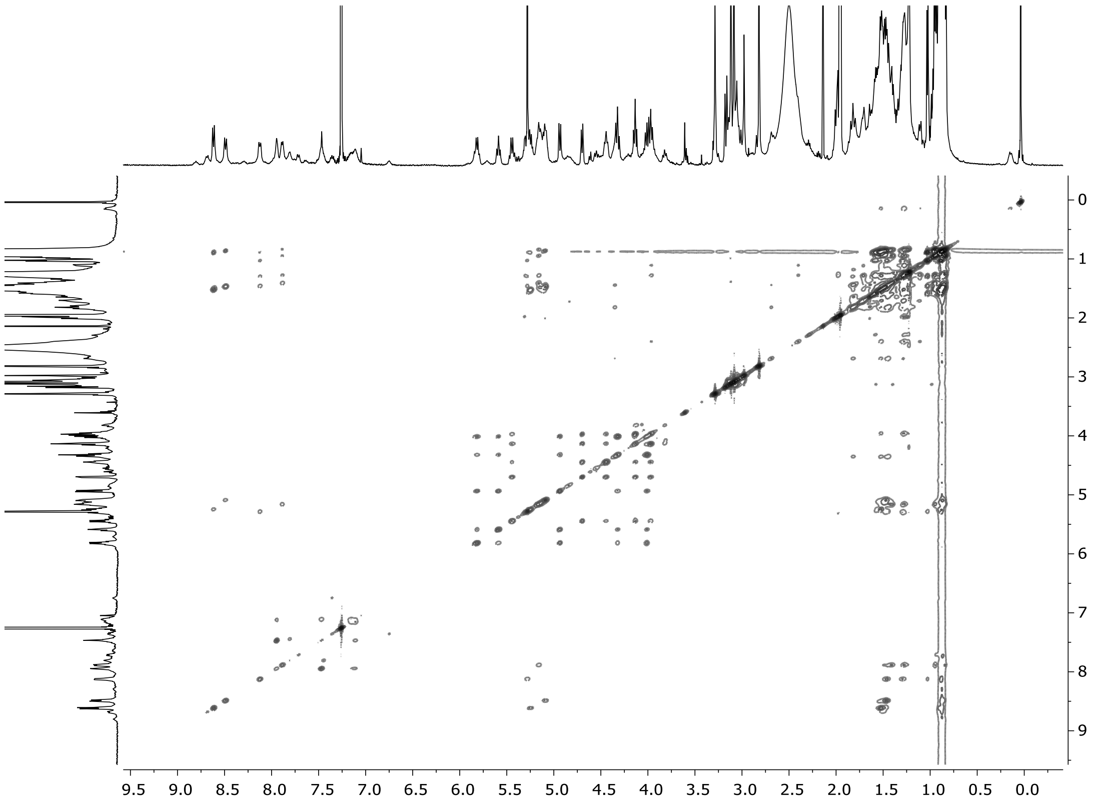

NOESY (5 mM, $CDCl_3$, 298 K, 500 MHz).

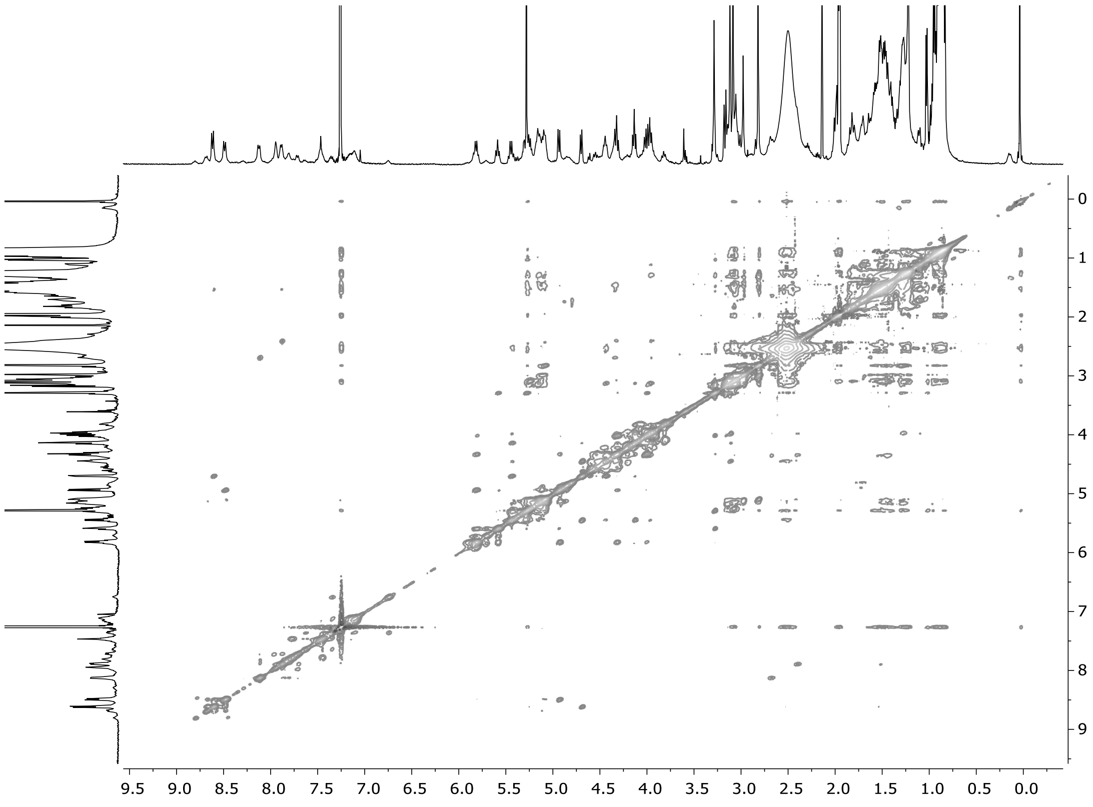

ROESY (5 mM, $CDCl_3$, 298 K, 500 MHz).

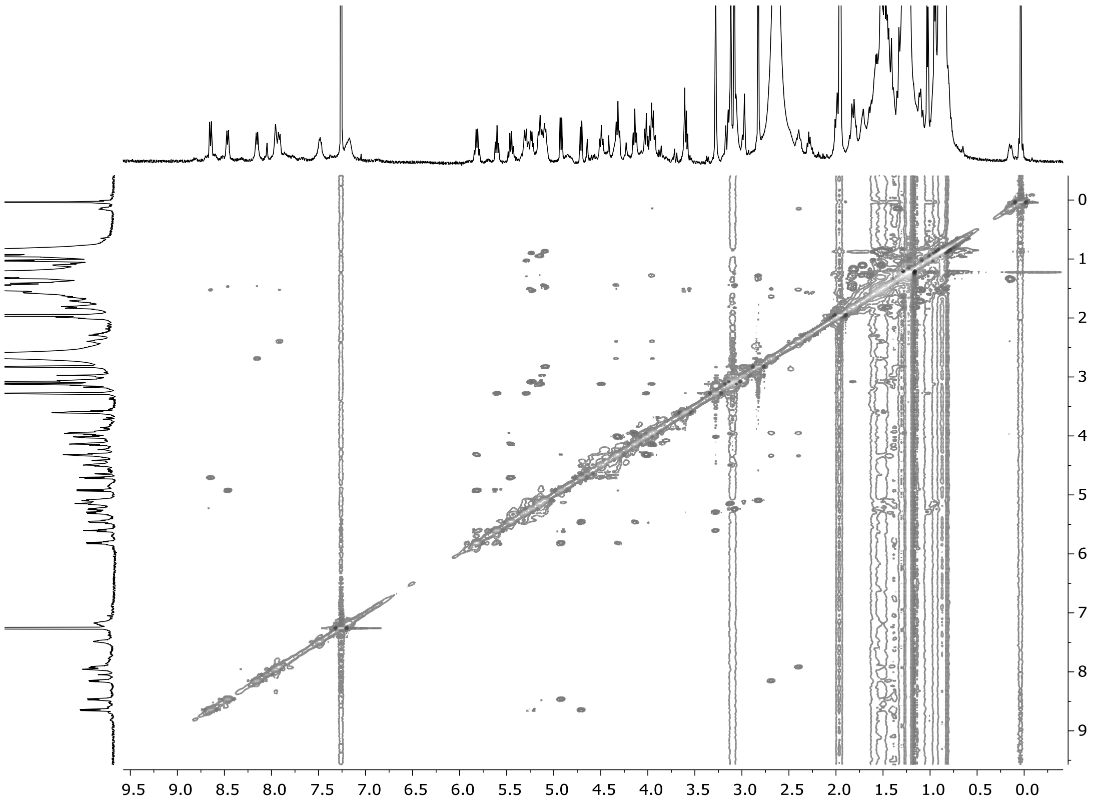

Computational methods:

The starting geometries of the cyclic peptides investigated in this work were constructed from X-ray crystallographic data of related compounds: *c*-[(*D*-Phe-*L*-^{Me}N-γ-Ach-)4][1] and previous simulations carried out with related systems[2]. The silver complex from these dimers was built using the optimized structures of bis(methyl picolinate) silver(I) complexes. All DFT calculations were carried out using the B3LYP[3] functional with the standard 6-31G(d) basis set for C, N, O and H[4] and Lanl2dz pseudopotential basis set for Ag[5], as implemented in the Gaussian 09[6] program package.

[1] Amorín, M., Castedo, L. and Granja, J. R.; Self-Assembled Peptide Tubulets with 7 Å Pores. *Chem. Eur. J.* **2005**, 11, 6543–6551.

[2] (a) Garcia-Fandino, R.; Castedo, L.; Granja, J. R.; Vázquez, S. Interaction and Dimerization Energies in Methyl-Blocked α,γ-Peptide Nanotube Segments. *J. Phys. Chem. B* **2010**, *114,* 4973–4983. (b) Garcia-Fandino, R.; Granja, J. R.; Marco, D. A.; Orozco, M. Theoretical characterization of the dynamical behavior and transport properties of α,γ-peptide nanotubes in solution. *J. Am. Chem. Soc.* **2009**, *131*, 15678-15686. (c) García-Fandiño, R; Granja, J. R. From α,γ-cyclic peptides to homo/hetero dimers and nanotubes in polar and non-polar solvents. Towards control of nanotube length: a computational study. *J. Phys. Chem. C*. **2013**, *117*, 10143-10162. (d) Garcia-Fandino, R.; Amorin, M.; Castedo, L.; Granja, J. R. Transmembrane ion transport by self-assembling α,γ-peptide nanotubes. *Chem. Sci*. **2012**, *3*, 3280-3285.

[3] Becke, A. D. Density-functional thermochemistry. III. The role of exact exchange, *J. Chem. Phys*. **1993**, *98*, 5648-52.

[4] Hehre, W. J.; Radom, L.; Schleyer, P. v. R.; Pople, J. A. *Ab Initio Molecular Orbital Theory*, 1st ed.; Wiley: New York, 1986.

[5] Hay, P. J.; Wadt, W. R. Ab initio effective core potentials for molecular calculations - potentials for the transition-metal atoms Sc to Hg. *J. Chem. Phys.* **1985**, *82*, 270-83.

[6] Frisch, M. J.; Trucks, G. W.; Schlegel, H. B.; Scuseria, G. E.; Robb, M. A.; Cheeseman, J. R. et al., *Gaussian 09, Revision A.01, Gaussian Inc.*, Wallingford, CT, USA, **2009**.

Geometries

Cartesian coordinates (Å) of conformations optimized at the B3LYP/6-31G(d) basis set for C, N, O and H and Lanl2dz pseudopotential basis set for Ag

Square planar bis(methyl picolinate) silver(I) complex

Center Number	Atomic Number	Atomic Type	Coordinates (Angstroms) X	Y	Z
1	6	0	4.129494	-3.235475	0.341259
2	6	0	3.326356	3.043674	-0.277417
3	6	0	2.106282	2.367067	-0.224799
4	7	0	2.023797	1.035051	-0.100148
5	6	0	3.164118	0.318997	-0.020096
6	6	0	4.423229	0.918155	-0.065254
7	6	0	4.503849	2.305581	-0.196990
8	6	0	2.998973	-1.171098	0.123802
9	8	0	4.169768	-1.796073	0.196482
10	8	0	1.917643	-1.736553	0.169547
11	6	0	-4.133667	3.233334	0.341120
12	8	0	-4.172184	1.793882	0.196269
13	6	0	-3.322343	-3.044836	-0.277613
14	6	0	-2.103201	-2.366545	-0.224914
15	7	0	-2.022628	-1.034423	-0.100252
16	6	0	-3.163919	-0.319958	-0.020261
17	6	0	-4.422205	-0.920830	-0.065500
18	6	0	-4.500866	-2.308371	-0.197258
19	6	0	-3.000653	1.170314	0.123702
20	8	0	-1.919984	1.737045	0.169578
21	47	0	-0.000048	0.001765	-0.022877
22	1	0	3.606483	-3.502706	1.261834
23	1	0	3.622283	-3.682319	-0.516340
24	1	0	5.171958	-3.546543	0.381762
25	1	0	3.340425	4.123593	-0.379162
26	1	0	1.164464	2.903538	-0.282526
27	1	0	5.313111	0.305151	0.002105
28	1	0	5.471180	2.796775	-0.234872
29	1	0	-5.176512	3.543130	0.381553
30	1	0	-3.611049	3.501150	1.261746
31	1	0	-3.626926	3.680825	-0.516419
32	1	0	-3.334935	-4.124773	-0.379366
33	1	0	-1.160595	-2.901643	-0.282567
34	1	0	-5.312946	-0.309063	0.001815
35	1	0	-5.467507	-2.800917	-0.235219

Perpendicularly oriented bis(methyl picolinate) silver(I) complex

Center Number	Atomic Number	Atomic Type	Coordinates (Angstroms) X	Y	Z
1	6	0	4.129494	-3.235475	0.341259
2	6	0	3.326356	3.043674	-0.277417
3	6	0	2.106282	2.367067	-0.224799
4	7	0	2.023797	1.035051	-0.100148
5	6	0	3.164118	0.318997	-0.020096
6	6	0	4.423229	0.918155	-0.065254
7	6	0	4.503849	2.305581	-0.196990
8	6	0	2.998973	-1.171098	0.123802
9	8	0	4.169768	-1.796073	0.196482
10	8	0	1.917643	-1.736553	0.169547
11	6	0	-4.133667	3.233334	0.341120
12	8	0	-4.172184	1.793882	0.196269
13	6	0	-3.322343	-3.044836	-0.277613
14	6	0	-2.103201	-2.366545	-0.224914
15	7	0	-2.022628	-1.034423	-0.100252
16	6	0	-3.163919	-0.319958	-0.020261
17	6	0	-4.422205	-0.920830	-0.065500
18	6	0	-4.500866	-2.308371	-0.197258
19	6	0	-3.000653	1.170314	0.123702
20	8	0	-1.919984	1.737045	0.169578
21	47	0	-0.000048	0.001765	-0.022877
22	1	0	3.606483	-3.502706	1.261834
23	1	0	3.622283	-3.682319	-0.516340
24	1	0	5.171958	-3.546543	0.381762
25	1	0	3.340425	4.123593	-0.379162
26	1	0	1.164464	2.903538	-0.282526
27	1	0	5.313111	0.305151	0.002105
28	1	0	5.471180	2.796775	-0.234872
29	1	0	-5.176512	3.543130	0.381553
30	1	0	-3.611049	3.501150	1.261746
31	1	0	-3.626926	3.680825	-0.516419
32	1	0	-3.334935	-4.124773	-0.379366
33	1	0	-1.160595	-2.901643	-0.282567
34	1	0	-5.312946	-0.309063	0.001815
35	1	0	-5.467507	-2.800917	-0.235219

***Syn*-eclipsed dimer**

Center Number	Atomic Number	Atomic Type	Coordinates (Angstroms) X	Y	Z
1	47	0	0.745805	-0.189113	0.018908
2	6	0	-5.781976	4.035521	-2.455178
3	6	0	0.647979	-2.253207	2.460937
4	6	0	-3.405474	4.624260	-2.630207
5	6	0	-2.272957	5.455639	-2.009179
6	6	0	-0.877064	4.831939	-2.179156
7	6	0	-0.027689	6.051546	-2.563265
8	6	0	1.329090	0.479310	5.006467
9	6	0	2.300218	5.571976	-2.195236
10	6	0	1.065067	-0.785365	4.478265
11	6	0	1.468979	5.508053	-4.546469
12	6	0	-1.017468	6.816821	-3.450465
13	6	0	0.939188	-0.923988	3.096730
14	6	0	1.319744	1.331999	2.765445
15	6	0	1.456522	1.558029	4.138536
16	6	0	-6.989822	4.890436	-2.048098
17	6	0	-6.009968	1.833796	-4.315361
18	6	0	-6.493472	0.237385	-2.408727
19	6	0	-5.433062	-0.813506	-2.782735
20	6	0	-7.896283	-0.156542	-2.902799
21	6	0	-8.281680	-1.546866	-2.377667
22	6	0	-7.244069	-2.603020	-2.777485
23	6	0	-5.821626	-2.220519	-2.295758
24	6	0	-4.834493	-3.267035	-2.820879
25	6	0	-6.489187	5.548111	2.166710
26	6	0	-3.941318	-5.519573	-2.516439
27	6	0	-4.562574	-6.865384	-2.109915
28	6	0	-2.524364	-5.402980	-1.928425
29	6	0	-5.431138	4.529140	2.610385
30	6	0	-5.717505	2.109857	2.956237
31	6	0	-1.602232	-6.181953	-4.104641
32	6	0	-0.131209	-5.589836	-2.142933
33	6	0	0.792498	-4.631379	-2.903513
34	6	0	-6.324012	0.825256	2.384819
35	6	0	0.783948	-6.817004	-2.013568
36	6	0	-7.814384	0.784817	2.808564
37	6	0	2.089633	-4.851865	-2.121011
38	6	0	3.344518	-4.384599	-2.867927
39	6	0	-8.494707	-0.525145	2.393972
40	6	0	-7.743104	-1.744072	2.946925
41	6	0	5.745795	-3.923297	-2.633005
42	6	0	6.888473	-4.840089	-2.182931
43	6	0	6.003468	-2.480723	-2.142935
44	6	0	-5.567177	-0.413473	2.903777
45	6	0	-6.272573	-1.720488	2.495847
46	6	0	5.818542	-1.712929	-4.497032
47	6	0	6.361428	-0.106622	-2.614725
48	6	0	5.285726	0.933644	-2.985386
49	6	0	7.754631	0.300028	-3.126537
50	6	0	8.137781	1.691247	-2.604435
51	6	0	7.085724	2.738230	-2.988136
52	6	0	5.670663	2.342168	-2.492718
53	6	0	4.691944	3.398841	-3.007341

54	6	0	-5.330387	-3.107485	4.399790
55	6	0	-5.231753	-3.888442	2.046524
56	6	0	3.768428	5.632268	-2.630728
57	6	0	4.381673	6.931777	-2.082390
58	6	0	-1.790729	1.149927	-3.466505
59	6	0	-0.959267	1.110087	-2.343485
60	6	0	-0.330709	-1.071866	-2.793510
61	6	0	-1.162239	-1.126287	-3.911450
62	6	0	-1.900279	0.006926	-4.253386
63	6	0	0.552492	-2.226962	-2.402762
64	6	0	4.958803	3.841854	1.888014
65	6	0	4.378042	5.168722	2.418406
66	6	0	1.945053	5.169256	2.778485
67	6	0	0.592597	5.591710	2.190575
68	6	0	-0.558892	4.620081	2.504466
69	6	0	-1.746620	5.601758	2.475401
70	6	0	-5.314404	-6.410550	2.063315
71	6	0	-4.049475	4.969195	2.094086
72	6	0	-4.537982	-5.177096	2.539251
73	6	0	-3.122948	4.730823	4.400495
74	6	0	-1.146676	6.814965	3.198777
75	6	0	-2.094715	-5.103035	2.859728
76	6	0	-0.752301	-5.038015	2.113174
77	6	0	1.037548	-6.568666	1.903663
78	6	0	5.272745	6.329859	1.963141
79	6	0	0.417383	-4.607065	3.007636
80	6	0	1.593450	-5.170796	2.205046
81	6	0	5.279757	3.109209	4.236991
82	6	0	6.110870	1.716989	2.291824
83	6	0	5.418304	0.403584	2.698595
84	6	0	7.587607	1.749960	2.723104
85	6	0	8.341347	0.531730	2.171850
86	6	0	7.673443	-0.779029	2.603692
87	6	0	6.183575	-0.828054	2.180807
88	6	0	5.569873	-2.117947	2.732450
89	6	0	3.148088	-5.195001	4.231549
90	6	0	3.946442	-4.836971	1.900460
91	6	0	5.360682	-4.541858	2.428545
92	6	0	6.334306	-5.630798	1.963397
93	6	0	-5.994518	2.591769	-1.951863
94	7	0	1.306777	5.749712	-3.104538
95	7	0	-5.746149	3.190137	2.123346
96	7	0	-6.118990	1.595078	-2.869871
97	7	0	-5.544711	-2.926243	2.956773
98	7	0	-4.757912	-4.406090	-2.068247
99	7	0	-3.168088	-5.159575	2.034423
100	7	0	-1.461199	-5.795016	-2.697746
101	7	0	4.461932	-4.370735	-2.105674
102	7	0	2.930400	-5.082450	2.783155
103	7	0	6.006560	-1.473279	-3.057666
104	7	0	5.768741	-3.224308	1.955273
105	7	0	4.499786	4.464125	-2.171511
106	7	0	5.383968	2.911998	2.784396
107	7	0	-0.243000	0.025169	-2.011761
108	7	0	-4.548841	4.594381	-1.910826
109	7	0	3.006789	5.346694	1.950860
110	7	0	-3.021298	5.102782	2.981060
111	7	0	1.070402	0.120601	2.249939
112	8	0	4.196730	3.349267	-4.135638

113	8	0	5.023159	-2.170172	3.836835
114	8	0	5.042791	3.684720	0.659529
115	8	0	3.748295	-4.822554	0.680014
116	8	0	-0.655258	3.501359	1.658595
117	8	0	0.229044	6.842290	2.790867
118	8	0	-3.915652	5.218711	0.885495
119	8	0	6.194626	-2.295336	-0.929732
120	8	0	-0.367494	-6.359553	1.672542
121	8	0	3.299725	-4.066699	-4.058314
122	8	0	2.122417	-6.289660	-2.001796
123	8	0	0.176148	-3.334989	-3.037377
124	8	0	-2.153093	-5.082498	4.089763
125	8	0	-2.379959	-4.992368	-0.771972
126	8	0	-5.460233	-3.769517	0.832285
127	8	0	-4.231942	-3.134168	-3.888508
128	8	0	-5.323155	2.175340	4.123470
129	8	0	-6.081664	2.397732	-0.730472
130	8	0	-3.269230	4.100109	-3.743846
131	8	0	-0.419769	4.170983	-1.001865
132	8	0	-2.235252	6.716116	-2.716045
133	8	0	2.041995	5.438246	-0.978679
134	8	0	0.525204	-3.216347	3.375511
135	8	0	1.476319	-2.125502	-1.616478
136	8	0	2.043067	4.746617	3.934315
137	8	0	0.535385	-2.418770	1.257974
138	1	0	-5.667373	4.070418	-3.535771
139	1	0	-2.474092	5.660288	-0.954487
140	1	0	-0.889338	4.120886	-3.015277
141	1	0	0.130760	6.645076	-1.658373
142	1	0	1.428108	0.613072	6.079438
143	1	0	0.953845	-1.652594	5.115956
144	1	0	0.581042	4.989977	-4.918346
145	1	0	1.585674	6.443702	-5.105185
146	1	0	2.323743	4.853414	-4.722098
147	1	0	-1.125326	6.370663	-4.448078
148	1	0	-0.777359	7.877930	-3.558330
149	1	0	1.373788	2.154452	2.064372
150	1	0	1.647194	2.568912	4.485364
151	1	0	-7.903047	4.509850	-2.518751
152	1	0	-7.135558	4.864521	-0.964615
153	1	0	-6.841213	5.926367	-2.366615
154	1	0	-5.960914	0.870835	-4.823449
155	1	0	-5.098477	2.388376	-4.550890
156	1	0	-6.876981	2.375382	-4.712313
157	1	0	-6.528305	0.317381	-1.320674
158	1	0	-4.466978	-0.519897	-2.355129
159	1	0	-5.292671	-0.868412	-3.868208
160	1	0	-7.921492	-0.168856	-4.001516
161	1	0	-8.623042	0.596228	-2.573595
162	1	0	-9.270648	-1.829382	-2.758095
163	1	0	-8.366164	-1.513398	-1.281673
164	1	0	-7.228711	-2.707225	-3.871657
165	1	0	-7.517797	-3.581684	-2.367979
166	1	0	-5.814678	-2.252909	-1.198506
167	1	0	-7.476363	5.230222	2.514744
168	1	0	-6.507262	5.647253	1.078357
169	1	0	-6.274124	6.536191	2.588938
170	1	0	-3.908028	-5.438070	-3.602477
171	1	0	-3.980192	-7.704297	-2.506939

172	1	0	-5.582865	-6.936582	-2.497717
173	1	0	-4.592762	-6.967504	-1.020607
174	1	0	-5.443385	4.455784	3.695703
175	1	0	-1.888302	-5.329760	-4.731304
176	1	0	-0.646890	-6.570993	-4.460774
177	1	0	-2.340538	-6.978886	-4.223433
178	1	0	-0.300987	-5.174461	-1.149503
179	1	0	0.971443	-4.943638	-3.935069
180	1	0	-6.274710	0.861139	1.289393
181	1	0	0.587493	-7.370525	-1.089376
182	1	0	0.683738	-7.508237	-2.860468
183	1	0	-7.867317	0.896917	3.900078
184	1	0	-8.340761	1.642837	2.373811
185	1	0	2.057042	-4.395626	-1.129750
186	1	0	-9.534151	-0.534176	2.743654
187	1	0	-8.534126	-0.588827	1.296480
188	1	0	-7.800412	-1.737694	4.044654
189	1	0	-8.212685	-2.674445	2.605293
190	1	0	5.654166	-3.972695	-3.715177
191	1	0	6.677951	-5.871656	-2.480010
192	1	0	7.832244	-4.530454	-2.645763
193	1	0	7.019442	-4.797634	-1.098484
194	1	0	-4.540933	-0.417433	2.514951
195	1	0	-5.493063	-0.317889	3.992529
196	1	0	-6.254892	-1.793149	1.406596
197	1	0	4.918413	-2.303847	-4.680744
198	1	0	5.698004	-0.751937	-4.995968
199	1	0	6.684237	-2.215356	-4.945294
200	1	0	6.409527	-0.175165	-1.526055
201	1	0	4.324211	0.629428	-2.552355
202	1	0	5.139304	0.992156	-4.069397
203	1	0	7.763167	0.311451	-4.225539
204	1	0	8.490171	-0.448113	-2.807030
205	1	0	9.118486	1.984284	-2.998267
206	1	0	8.238391	1.655658	-1.509686
207	1	0	7.054484	2.845459	-4.081048
208	1	0	7.354995	3.718994	-2.578635
209	1	0	5.671271	2.365788	-1.395760
210	1	0	-5.536182	-2.163619	4.903012
211	1	0	-6.000340	-3.865534	4.822410
212	1	0	-4.295981	-3.384213	4.618205
213	1	0	3.852312	5.635977	-3.715700
214	1	0	5.435562	6.987370	-2.369663
215	1	0	4.307852	6.974622	-0.992400
216	1	0	3.863975	7.806280	-2.491304
217	1	0	-2.340278	2.058877	-3.696020
218	1	0	-0.855737	1.972070	-1.691395
219	1	0	-1.231401	-2.037633	-4.490037
220	1	0	-2.551965	-0.011791	-5.121146
221	1	0	4.312933	5.176967	3.503215
222	1	0	0.677520	5.723521	1.104013
223	1	0	-0.407640	4.227549	3.515330
224	1	0	-1.929852	5.871765	1.434555
225	1	0	-4.782540	-7.320725	2.355385
226	1	0	-5.437878	-6.397546	0.977459
227	1	0	-6.313275	-6.431857	2.514035
228	1	0	-4.448985	-5.210703	3.622357
229	1	0	-2.117478	4.559496	4.788473
230	1	0	-3.583728	5.527780	4.996738

231	1	0	-3.692188	3.806924	4.521318
232	1	0	-1.208194	6.719461	4.291466
233	1	0	-1.614493	7.760439	2.906534
234	1	0	-0.849799	-4.384444	1.242596
235	1	0	1.182919	-7.256214	2.749065
236	1	0	1.491170	-7.003550	1.008882
237	1	0	6.277711	6.227019	2.387446
238	1	0	5.370806	6.343680	0.874197
239	1	0	4.850724	7.281393	2.299587
240	1	0	0.285369	-5.093412	3.975437
241	1	0	1.650157	-4.628396	1.262380
242	1	0	5.490239	2.161638	4.732111
243	1	0	6.000147	3.848639	4.606817
244	1	0	4.270241	3.417514	4.516874
245	1	0	6.072618	1.800942	1.204055
246	1	0	5.340247	0.311544	3.787558
247	1	0	4.392664	0.399928	2.308348
248	1	0	8.048220	2.681378	2.371419
249	1	0	7.659802	1.750109	3.819656
250	1	0	8.369338	0.584062	1.073717
251	1	0	9.383784	0.552336	2.511719
252	1	0	8.203032	-1.636608	2.174019
253	1	0	7.733552	-0.880071	3.696614
254	1	0	6.143119	-0.861143	1.084854
255	1	0	3.898854	-5.957436	4.458687
256	1	0	2.218703	-5.506991	4.707888
257	1	0	3.462989	-4.236524	4.655780
258	1	0	5.369506	-4.476329	3.514901
259	1	0	7.349105	-5.390202	2.293486
260	1	0	6.054845	-6.604042	2.382737
261	1	0	6.325912	-5.722458	0.873553
262	1	0	-5.932267	3.057748	1.129089
263	1	0	-5.030654	-4.362519	-1.086321
264	1	0	-3.025962	-5.111089	1.024295
265	1	0	4.375677	-4.535119	-1.102101
266	1	0	5.989668	-3.099211	0.966631
267	1	0	4.729940	4.375251	-1.181495
268	1	0	-4.530701	4.896151	-0.935128
269	1	0	2.845941	5.546768	0.965665
270	1	0	-0.850610	3.799046	0.745923
271	1	0	0.511407	4.459131	-0.857415

***Anti*-eclipsed dimer**

1	6	0	-5.387776	-0.955010	-3.136076
2	6	0	5.480198	3.116242	4.348196
3	6	0	3.662393	-4.386280	-3.029401
4	6	0	2.351676	-4.727118	-2.295421
5	6	0	1.123014	-4.294475	-3.098874
6	6	0	0.045984	-5.193872	-2.490481
7	6	0	-3.299278	4.828267	4.232950
8	6	0	-2.310339	-5.553279	-2.278560
9	6	0	-5.196808	-3.275313	4.121662
10	6	0	0.837204	-6.516682	-2.467108
11	6	0	1.490320	1.157781	-3.736160
12	6	0	1.341026	-0.217391	-3.544760
13	6	0	0.760354	-0.669921	-2.359696
14	6	0	7.175486	-4.813252	-2.240769
15	6	0	6.289164	-2.449338	-2.196173
16	6	0	0.431975	1.489087	-1.600166
17	6	0	1.014555	2.018878	-2.754446
18	6	0	6.553516	-0.049735	-2.601221
19	6	0	7.891437	0.499800	-3.123281
20	6	0	8.150248	1.903071	-2.556186
21	6	0	7.000750	2.862128	-2.888793
22	6	0	5.379964	0.899246	-2.910225
23	6	0	5.636235	2.324463	-2.385770
24	6	0	4.545858	3.288820	-2.862538
25	6	0	3.213977	-4.956738	4.081913
26	6	0	-0.565997	1.979656	2.845839
27	6	0	3.503995	5.474887	-2.487132
28	6	0	4.207389	6.818941	-2.224282
29	6	0	2.147537	5.483096	-1.763056
30	6	0	-1.419992	-0.474277	5.619783
31	6	0	-1.118267	0.726349	4.974003
32	6	0	-0.221859	6.060808	-1.734840
33	6	0	-0.918215	7.402655	-2.085649
34	6	0	-0.861966	0.712210	3.602446
35	6	0	-1.329518	5.005132	-2.033467
36	6	0	-2.594063	5.885451	-2.164042
37	6	0	-3.680743	5.239806	-3.025815
38	6	0	-1.185757	-1.575422	3.504091
39	6	0	-1.459702	-1.645328	4.871839
40	6	0	-5.720393	3.881281	-2.990583
41	6	0	-7.051305	4.654059	-2.903442
42	6	0	-5.904650	2.489090	-2.366768
43	6	0	-6.393542	0.100876	-2.641590
44	6	0	-7.850836	-0.323762	-2.895794
45	6	0	-8.133207	-1.700178	-2.277074
46	6	0	-7.148802	-2.759117	-2.788844
47	6	0	0.647443	-2.133131	-2.060204
48	6	0	-5.679400	-2.341371	-2.533343
49	6	0	-4.757289	-3.400316	-3.142589
50	6	0	6.430380	-5.529553	1.809184
51	6	0	-3.747954	-5.622363	-2.823220
52	6	0	-4.406945	-6.939057	-2.396994
53	6	0	-1.438632	-5.055791	-4.567257
54	6	0	6.146156	-1.635235	-4.538279
55	6	0	1.036073	6.062794	-3.922683
56	6	0	5.462940	-4.438430	2.290393
57	6	0	-6.126927	1.598000	-4.667647

58	6	0	6.049864	-3.887068	-2.711591
59	6	0	-4.347967	-5.314492	2.241791
60	6	0	5.781664	-2.045622	2.698181
61	6	0	-1.910339	-5.194146	2.537743
62	6	0	-0.558601	-5.527724	1.890354
63	6	0	0.582804	-4.602181	2.323002
64	6	0	1.788603	-5.525482	2.071786
65	6	0	6.419358	-0.750736	2.187844
66	6	0	4.055958	-4.745360	1.749185
67	6	0	5.657334	0.474084	2.727355
68	6	0	1.245847	-6.868958	2.587145
69	6	0	7.901432	-0.728057	2.638510
70	6	0	8.589076	0.590229	2.263082
71	6	0	7.837370	1.797155	2.842092
72	6	0	-5.223218	-6.479023	1.759499
73	6	0	-4.960062	-3.983394	1.752566
74	6	0	6.370324	1.793554	2.379052
75	6	0	5.212833	3.920244	2.013759
76	6	0	-6.141977	-1.890669	2.225653
77	6	0	-7.592646	-1.943276	2.736813
78	6	0	-8.395313	-0.743138	2.215300
79	6	0	-7.727632	0.583680	2.595712
80	6	0	-5.452314	-0.562817	2.591761
81	6	0	-6.261222	0.653284	2.099765
82	6	0	-5.651778	1.958359	2.615561
83	6	0	-5.373481	4.360606	2.211362
84	6	0	-6.242417	5.440555	1.555726
85	6	0	-3.898038	4.565393	1.832939
86	6	0	5.217665	6.441457	2.044580
87	6	0	-1.580868	4.880714	2.340787
88	6	0	-1.077131	6.305011	2.063521
89	6	0	4.515894	5.183408	2.568528
90	6	0	-0.429309	4.388134	3.227812
91	6	0	2.109639	4.867031	3.020170
92	6	0	0.739189	4.832179	2.335615
93	1	0	-2.158518	4.129232	-0.474399
94	1	0	-2.833591	-5.686533	0.768422
95	1	0	-4.704481	-4.405545	-1.335266
96	1	0	-5.944512	2.877475	0.784105
97	1	0	-4.564199	4.494772	-1.300130
98	1	0	4.674783	4.342891	-1.088438
99	1	0	2.904585	5.219597	1.162384
100	1	0	6.138009	-2.968269	0.880324
101	1	0	4.615318	-4.440279	-1.211254
102	1	0	0.799662	4.218590	1.431637
103	1	0	-0.349611	4.936813	4.167971
104	1	0	4.508339	5.203027	3.655716
105	1	0	-1.340373	6.993414	2.878610
106	1	0	-1.469298	6.709186	1.125003
107	1	0	-1.521377	4.321597	1.410560
108	1	0	5.250122	6.439667	0.951695
109	1	0	6.249515	6.485463	2.410755
110	1	0	4.688145	7.334901	2.388017
111	1	0	-6.135515	5.424332	0.467434
112	1	0	-7.292659	5.272113	1.810715
113	1	0	-5.956150	6.437180	1.909523
114	1	0	-5.510833	4.387054	3.290620
115	1	0	-6.267855	0.675540	1.001944
116	1	0	-5.331437	-0.460567	3.676109

117	1	0	-4.444886	-0.546438	2.156720
118	1	0	-8.293375	1.428222	2.185397
119	1	0	-7.730609	0.697920	3.688396
120	1	0	-8.484371	-0.809818	1.121338
121	1	0	-9.416802	-0.775136	2.612699
122	1	0	-8.056562	-2.884691	2.418220
123	1	0	-7.606299	-1.933982	3.835580
124	1	0	-6.162200	-1.976473	1.137497
125	1	0	6.359248	1.915290	1.294072
126	1	0	-5.323135	-6.466335	0.670812
127	1	0	-6.228150	-6.406494	2.189862
128	1	0	-4.781328	-7.430620	2.069001
129	1	0	7.887498	1.758743	3.939221
130	1	0	8.314188	2.734995	2.531925
131	1	0	9.625719	0.587804	2.620996
132	1	0	8.637069	0.680253	1.167869
133	1	0	7.938388	-0.865165	3.728174
134	1	0	8.432265	-1.576540	2.191477
135	1	0	1.685845	-7.726767	2.067836
136	1	0	0.690841	-3.445503	0.725804
137	1	0	1.410023	-6.996656	3.665420
138	1	0	6.154982	3.876196	4.759993
139	1	0	4.450152	3.366149	4.613276
140	1	0	5.725286	2.164393	4.818364
141	1	0	2.366226	-4.263551	-1.302979
142	1	0	1.322770	-4.525490	-4.145857
143	1	0	-0.126742	-4.883816	-1.463108
144	1	0	-3.882707	5.727281	4.457724
145	1	0	-3.854116	3.936388	4.534979
146	1	0	-2.377123	4.878029	4.810440
147	1	0	-5.362854	-2.324498	4.628499
148	1	0	-4.178973	-3.601603	4.344308
149	1	0	-5.910715	-4.002135	4.527930
150	1	0	0.804763	-7.018414	-3.444492
151	1	0	0.465885	-7.204419	-1.702793
152	1	0	1.990784	1.549322	-4.614524
153	1	0	1.689546	-0.933200	-4.278572
154	1	0	6.962150	-5.843129	-2.541557
155	1	0	8.128848	-4.511215	-2.688721
156	1	0	7.288706	-4.770562	-1.154770
157	1	0	0.042064	2.141889	-0.829083
158	1	0	1.104791	3.092646	-2.857541
159	1	0	6.634096	-0.155853	-1.517806
160	1	0	8.700836	-0.185321	-2.843549
161	1	0	7.878564	0.551756	-4.220975
162	1	0	9.093440	2.299015	-2.951486
163	1	0	8.270801	1.839781	-1.464847
164	1	0	6.942343	3.001317	-3.977399
165	1	0	7.188809	3.848409	-2.449572
166	1	0	5.203078	0.971660	-3.989259
167	1	0	4.462403	0.492724	-2.466689
168	1	0	5.653387	2.325282	-1.288285
169	1	0	3.690889	-4.006215	4.332107
170	1	0	2.221513	-4.966839	4.535261
171	1	0	3.789233	-5.783762	4.514549
172	1	0	3.378053	5.334631	-3.559436
173	1	0	3.611847	7.654726	-2.608651
174	1	0	4.354436	6.976358	-1.150944
175	1	0	5.182537	6.831422	-2.719612

176	1	0	-1.619389	-0.484384	6.687081
177	1	0	-1.078888	1.661521	5.516482
178	1	0	0.045723	6.014389	-0.679836
179	1	0	-0.329035	8.059053	-2.729813
180	1	0	-1.157032	7.954197	-1.162983
181	1	0	-1.134535	4.550273	-3.012155
182	1	0	-2.988167	6.107027	-1.159100
183	1	0	-1.180984	-2.469068	2.897929
184	1	0	-1.686312	-2.612508	5.306850
185	1	0	-5.410118	3.827135	-4.033003
186	1	0	-7.844600	4.141314	-3.460310
187	1	0	-7.372840	4.746021	-1.860796
188	1	0	-6.923194	5.654503	-3.326296
189	1	0	-6.260366	0.225336	-1.565058
190	1	0	-8.526974	0.433692	-2.481015
191	1	0	-8.047417	-0.371020	-3.975966
192	1	0	-9.162904	-2.005732	-2.498339
193	1	0	-8.056442	-1.631092	-1.182017
194	1	0	-7.291222	-2.905092	-3.868677
195	1	0	-7.344305	-3.724183	-2.307464
196	1	0	-5.415507	-1.053316	-4.226759
197	1	0	-4.370644	-0.635452	-2.876436
198	1	0	-5.517858	-2.318792	-1.448180
199	1	0	6.135463	-6.507580	2.205711
200	1	0	7.443214	-5.305882	2.157032
201	1	0	6.434278	-5.598370	0.718158
202	1	0	-3.771250	-5.547194	-3.908098
203	1	0	-3.874886	-7.793639	-2.829845
204	1	0	-5.442864	-6.963297	-2.747396
205	1	0	-4.392398	-7.053341	-1.310185
206	1	0	-2.329998	-4.460095	-4.774298
207	1	0	-0.582148	-4.506379	-4.957240
208	1	0	-1.497356	-6.014206	-5.096941
209	1	0	5.265467	-2.241971	-4.762273
210	1	0	7.032505	-2.109729	-4.975693
211	1	0	6.017741	-0.665586	-5.018725
212	1	0	1.595869	6.964522	-4.196911
213	1	0	0.004332	6.192245	-4.255690
214	1	0	1.460930	5.209316	-4.458311
215	1	0	5.471756	-4.410080	3.377509
216	1	0	-6.859347	2.344608	-4.988822
217	1	0	-5.141938	1.874377	-5.057378
218	1	0	-6.408690	0.647721	-5.119148
219	1	0	5.983247	-3.927580	-3.795982
220	1	0	-4.276166	-5.350096	3.325967
221	1	0	-0.664862	-5.515471	0.796621
222	1	0	0.465889	-4.394551	3.390978
223	1	0	1.946570	-5.598802	0.993438
224	1	0	6.396561	-0.753302	1.090526
225	1	0	5.556551	0.346965	3.810781
226	1	0	4.639418	0.494865	2.317598
227	7	0	4.743233	-4.318028	-2.218405
228	7	0	5.890452	-3.116012	1.858652
229	7	0	-1.275291	-5.240381	-3.116703
230	7	0	5.636696	2.973280	2.892740
231	7	0	0.325411	0.168255	-1.396007
232	7	0	3.116658	5.142348	2.158577
233	7	0	6.294866	-1.424275	-3.090432
234	7	0	4.323423	4.358047	-2.046053

235	7	0	1.032290	5.834093	-2.470169
236	7	0	-2.960573	4.748435	2.802409
237	7	0	-4.643584	4.585271	-2.310036
238	7	0	-5.784056	3.029985	1.780304
239	7	0	-6.112263	1.439933	-3.207332
240	7	0	-5.371076	-3.072204	2.677341
241	7	0	-4.488326	-4.464830	-2.330885
242	7	0	-2.981485	-5.451624	1.750463
243	7	0	3.068839	-5.092171	2.624833
244	7	0	-0.888642	-0.428020	2.879645
245	8	0	-4.354707	-3.329133	-4.306004
246	8	0	-5.086125	-3.803876	0.531472
247	8	0	-1.422809	3.954167	-1.095253
248	8	0	-5.166050	2.052577	3.746007
249	8	0	0.620922	-3.342266	1.690348
250	8	0	3.870563	-4.720088	0.523526
251	8	0	-0.166076	-6.838320	2.322202
252	8	0	-5.905137	2.376518	-1.130490
253	8	0	-3.600038	4.542821	0.623215
254	8	0	-3.648794	5.288412	-4.248653
255	8	0	-2.119419	7.067593	-2.780269
256	8	0	0.350641	6.177563	1.978065
257	8	0	2.106933	5.230817	-0.553289
258	8	0	-0.518025	3.024586	3.676558
259	8	0	3.978690	3.155850	-3.952431
260	8	0	2.239804	4.647404	4.225204
261	8	0	6.443018	-2.280590	-0.976338
262	8	0	5.348114	3.813224	0.783956
263	8	0	3.677406	-4.164819	-4.242329
264	8	0	0.912960	-2.866276	-3.137779
265	8	0	2.189338	-6.145726	-2.148489
266	8	0	-2.120557	-5.765349	-1.071716
267	8	0	5.284806	-2.130704	3.824062
268	8	0	-1.991237	-4.752890	3.690027
269	8	0	0.361046	-2.573422	-0.955897
270	8	0	-0.402850	2.037634	1.640282
271	47	0	-0.302711	-0.482973	0.691518

Clockwise alternating dimer

Center Number	Atomic Number	Atomic Type	Coordinates (Angstroms) X	Y	Z
1	47	0	0.510593	-1.809849	-2.080444
2	6	0	4.279597	5.271016	2.563575
3	6	0	-2.602003	-1.373810	-3.099635
4	6	0	1.827602	5.307668	2.532936
5	6	0	0.616626	5.930012	1.826648
6	6	0	-0.661736	5.071284	1.874243
7	6	0	-1.715448	6.093595	2.341645
8	6	0	-1.586243	-1.725562	-6.745955
9	6	0	-3.917427	5.187518	2.001785
10	6	0	-2.380163	-1.488472	-5.623746
11	6	0	-3.034200	5.266145	4.340110
12	6	0	-0.881451	6.991000	3.264789
13	6	0	-1.790521	-1.541231	-4.359047
14	6	0	0.270078	-2.066596	-5.265624
15	6	0	-0.239173	-2.026371	-6.565402
16	6	0	5.285399	6.383582	2.220859
17	6	0	5.060153	3.312063	4.544868
18	6	0	6.282239	1.987359	2.762456
19	6	0	5.770023	0.589042	3.154662
20	6	0	7.685662	2.259188	3.331688
21	6	0	8.670837	1.185988	2.845125
22	6	0	8.185675	-0.224279	3.200021
23	6	0	6.759105	-0.498718	2.673879
24	6	0	6.323398	-1.884471	3.160156
25	6	0	-7.041655	4.497414	-2.222015
26	6	0	5.781995	-4.205023	2.457188
27	6	0	6.789673	-5.067499	1.691870
28	6	0	4.337246	-4.591565	2.068855
29	6	0	-5.734862	3.749051	-2.520687
30	6	0	-3.532140	4.727903	-3.005708
31	6	0	3.446678	-3.629295	4.229416
32	6	0	1.968067	-4.588928	2.446018
33	6	0	0.829357	-3.669915	2.888254
34	6	0	1.395248	-5.934695	2.914117
35	6	0	-2.407452	5.628814	-2.491747
36	6	0	-0.417855	-4.487952	2.522176
37	6	0	-1.566562	-4.246048	3.521121
38	6	0	-2.695525	7.076110	-2.960595
39	6	0	-1.573692	8.033507	-2.542667
40	6	0	-3.977514	-4.468695	3.863220
41	6	0	-4.825744	-5.741779	3.989906
42	6	0	-4.796249	-3.357886	3.172443
43	6	0	-0.211876	7.556315	-3.065187
44	6	0	-1.037244	5.141505	-3.006921
45	6	0	-5.029573	-2.272107	5.387057
46	6	0	-6.076759	-1.275135	3.300561
47	6	0	-5.459850	0.118887	3.527288
48	6	0	-7.548461	-1.330235	3.745540
49	6	0	-8.369306	-0.237470	3.045145
50	6	0	-7.772157	1.154927	3.286992
51	6	0	-6.287906	1.230323	2.852970
52	6	0	-5.751708	2.610180	3.233690
53	6	0	0.082706	6.116914	-2.601803

54	6	0	1.651994	5.511002	-4.505831
55	6	0	-5.350871	4.911788	2.474902
56	6	0	-6.308601	5.875454	1.758749
57	6	0	1.337378	2.107938	0.252358
58	6	0	1.188524	1.126574	-0.730956
59	6	0	2.448380	5.648707	-2.168189
60	6	0	1.064289	-0.553264	0.842157
61	6	0	1.208820	0.358477	1.889269
62	6	0	1.346244	1.714050	1.588241
63	6	0	0.909542	-2.027383	1.087336
64	6	0	-2.919110	-6.093947	-0.711255
65	6	0	-4.426093	-5.957671	-1.028912
66	6	0	-4.717276	-3.657497	-1.797556
67	6	0	-4.931445	-2.209230	-1.364634
68	6	0	-4.979065	-1.196150	-2.520470
69	6	0	-5.447155	0.026780	-1.723200
70	6	0	4.610504	6.871110	-2.421560
71	6	0	-5.699428	2.405609	-1.772621
72	6	0	3.894323	5.537790	-2.688798
73	6	0	-6.258361	1.113229	-3.851814
74	6	0	-6.580932	-0.629637	-0.905086
75	6	0	4.761871	3.242756	-2.676747
76	6	0	5.690503	2.258960	-1.957192
77	6	0	-5.235651	-6.904544	-0.138380
78	6	0	7.151732	0.570777	-2.357103
79	6	0	4.967804	1.011768	-1.376603
80	6	0	-2.476273	-6.702957	-3.078111
81	6	0	-0.626137	-6.645127	-1.338365
82	6	0	0.194807	-5.480420	-1.925876
83	6	0	-0.065572	-8.015726	-1.750357
84	6	0	1.421702	-8.117222	-1.381814
85	6	0	2.242468	-6.981441	-2.008380
86	6	0	1.690895	-5.594850	-1.588990
87	6	0	2.498900	-4.473227	-2.252148
88	6	0	5.977354	-0.140446	-1.654658
89	6	0	4.962237	-0.955225	-3.811889
90	6	0	4.766322	-3.629807	-2.692931
91	6	0	5.955401	-4.495659	-3.146325
92	6	0	5.297158	-2.462078	-1.849472
93	6	0	4.896809	3.922325	2.154424
94	7	0	-0.483801	-1.815482	-4.188385
95	7	0	-2.956799	5.533529	2.896725
96	7	0	-4.561694	4.517350	-2.124602
97	7	0	5.344960	3.084545	3.119002
98	7	0	1.427955	5.688998	-3.063928
99	7	0	6.008642	-2.784329	2.188533
100	7	0	4.609434	4.441605	-2.050988
101	7	0	3.308763	-4.253119	2.912172
102	7	0	-2.767667	-4.693013	3.088312
103	7	0	-5.270276	-2.338220	3.939830
104	7	0	5.403936	-1.238446	-2.437871
105	7	0	-5.702851	3.521612	2.218186
106	7	0	3.815014	-4.427402	-1.930195
107	7	0	1.052933	-0.175200	-0.450712
108	7	0	-2.060886	-6.500523	-1.683261
109	7	0	3.010796	5.510692	1.897704
110	7	0	-4.812085	-4.565859	-0.803762
111	7	0	-5.803251	1.232921	-2.467567
112	8	0	-5.521832	2.397377	-0.547376

113	8	0	-6.225059	-2.019691	-0.778927
114	8	0	-4.426954	-3.921724	-2.969722
115	8	0	-2.540753	-5.826701	0.438265
116	8	0	0.877962	-2.309733	2.388823
117	8	0	1.998953	-3.699823	-3.092842
118	8	0	5.660211	-2.690952	-0.689737
119	8	0	-5.465261	2.893882	4.398964
120	8	0	4.669546	1.098335	0.000954
121	8	0	-5.001959	-3.433813	1.952978
122	8	0	6.601953	1.756882	-2.922918
123	8	0	-1.376082	-3.691508	4.605635
124	8	0	0.005293	-5.865826	2.582860
125	8	0	4.236724	2.955517	-3.749914
126	8	0	4.116690	-5.183667	1.004839
127	8	0	2.280786	5.760471	-0.942055
128	8	0	6.296404	-2.154329	4.363673
129	8	0	-3.544235	4.296116	-4.157457
130	8	0	5.027186	3.690879	0.931746
131	8	0	1.727304	4.749927	3.630786
132	8	0	-0.929796	4.499327	0.604883
133	8	0	0.323102	7.161281	2.525820
134	8	0	-3.673281	5.140151	0.778352
135	8	0	-3.849188	-0.982381	-3.386491
136	8	0	0.821595	-2.856064	0.192478
137	8	0	-2.170598	-1.576167	-1.980751
138	1	0	4.064297	5.299690	3.629176
139	1	0	0.856958	6.179558	0.792282
140	1	0	-0.559164	4.285794	2.637599
141	1	0	-2.000493	6.686358	1.467556
142	1	0	-2.019288	-1.688112	-7.740932
143	1	0	-3.437977	-1.281106	-5.715598
144	1	0	-3.199727	6.187121	4.911270
145	1	0	-3.825543	4.548022	4.557346
146	1	0	-2.091259	4.818926	4.668387
147	1	0	-0.679246	6.525672	4.238338
148	1	0	-1.318715	7.980320	3.425971
149	1	0	1.304069	-2.327700	-5.067491
150	1	0	0.412139	-2.233726	-7.408139
151	1	0	4.872365	7.355323	2.505992
152	1	0	6.228112	6.234180	2.758606
153	1	0	5.505995	6.391168	1.148640
154	1	0	5.675550	4.113978	4.968782
155	1	0	5.279248	2.394444	5.090708
156	1	0	4.003166	3.545940	4.689524
157	1	0	6.345476	2.015825	1.675144
158	1	0	4.787176	0.423931	2.699787
159	1	0	5.661880	0.490745	4.241703
160	1	0	7.656330	2.255312	4.430009
161	1	0	8.021252	3.256617	3.021287
162	1	0	9.660844	1.362618	3.282019
163	1	0	8.791315	1.274492	1.755390
164	1	0	8.176063	-0.358256	4.288448
165	1	0	8.877731	-0.974394	2.797270
166	1	0	6.771289	-0.481900	1.576450
167	1	0	-7.167653	4.647224	-1.145145
168	1	0	-7.909289	3.933655	-2.585580
169	1	0	-7.032827	5.473709	-2.715073
170	1	0	5.937214	-4.338569	3.529068
171	1	0	6.612188	-6.130927	1.879842

172	1	0	7.803242	-4.817286	2.019774
173	1	0	6.706300	-4.890656	0.617195
174	1	0	-5.616917	3.607617	-3.594474
175	1	0	2.897868	-2.681974	4.255959
176	1	0	3.042324	-4.286140	5.009167
177	1	0	4.484754	-3.400142	4.460755
178	1	0	2.009872	-4.602961	1.360309
179	1	0	0.814426	-3.514433	3.967075
180	1	0	1.837137	-6.781969	2.383857
181	1	0	1.536771	-6.080285	3.995685
182	1	0	-2.401252	5.622470	-1.396422
183	1	0	-0.761847	-4.286865	1.504913
184	1	0	-2.798428	7.075700	-4.054150
185	1	0	-3.655763	7.410922	-2.549978
186	1	0	-1.782939	9.044312	-2.913120
187	1	0	-1.539045	8.102503	-1.445885
188	1	0	-3.634328	-4.159851	4.848209
189	1	0	-5.697119	-5.563363	4.630598
190	1	0	-5.190757	-6.064565	3.010548
191	1	0	-4.229814	-6.547124	4.428958
192	1	0	-0.210534	7.603192	-4.163618
193	1	0	0.587925	8.220229	-2.714481
194	1	0	-0.816043	4.148962	-2.595907
195	1	0	-1.114881	5.031908	-4.094140
196	1	0	-5.569848	-1.417970	5.793894
197	1	0	-5.397699	-3.166078	5.900579
198	1	0	-3.966306	-2.141997	5.613623
199	1	0	-6.037687	-1.510755	2.234090
200	1	0	-4.432412	0.127342	3.141855
201	1	0	-5.399886	0.357471	4.595023
202	1	0	-7.620466	-1.190735	4.833504
203	1	0	-7.956902	-2.322558	3.520931
204	1	0	-9.407150	-0.264105	3.398472
205	1	0	-8.403652	-0.444347	1.965783
206	1	0	-7.830223	1.402564	4.355080
207	1	0	-8.351202	1.917805	2.752031
208	1	0	-6.233245	1.118813	1.762619
209	1	0	0.144630	6.105122	-1.512934
210	1	0	0.685813	5.397310	-4.996071
211	1	0	2.158979	6.371714	-4.958362
212	1	0	2.233694	4.605409	-4.694302
213	1	0	-5.448656	5.053003	3.549210
214	1	0	-6.069911	6.914801	2.010470
215	1	0	-7.335773	5.670223	2.074012
216	1	0	-6.237824	5.766652	0.673401
217	1	0	1.431290	3.150027	-0.032687
218	1	0	1.173599	1.395394	-1.783157
219	1	0	1.206445	0.005844	2.913016
220	1	0	1.454009	2.445066	2.384851
221	1	0	-4.642019	-6.172362	-2.073206
222	1	0	-4.146360	-1.949209	-0.644780
223	1	0	-5.750671	-1.541558	-3.212711
224	1	0	-4.645159	0.319315	-1.041239
225	1	0	4.103429	7.692600	-2.939531
226	1	0	5.642876	6.819740	-2.779512
227	1	0	4.612575	7.101204	-1.351261
228	1	0	3.923728	5.320007	-3.753431
229	1	0	-5.429768	0.881919	-4.529295
230	1	0	-7.009277	0.319221	-3.923523

231	1	0	-6.735051	2.035549	-4.178440
232	1	0	-7.542185	-0.558541	-1.429998
233	1	0	-6.685888	-0.175908	0.082816
234	1	0	6.237811	2.761659	-1.145290
235	1	0	-6.305799	-6.762686	-0.315766
236	1	0	-4.980545	-7.946692	-0.358644
237	1	0	-5.020082	-6.720286	0.916969
238	1	0	7.609339	-0.009603	-3.161715
239	1	0	7.930529	0.825710	-1.621209
240	1	0	4.045683	0.828866	-1.945242
241	1	0	-1.587047	-6.707346	-3.709519
242	1	0	-3.118496	-5.885818	-3.413568
243	1	0	-2.989760	-7.661968	-3.217619
244	1	0	-0.590321	-6.575378	-0.248497
245	1	0	-0.208573	-4.539549	-1.538074
246	1	0	0.086441	-5.446360	-3.016634
247	1	0	-0.172401	-8.167330	-2.833258
248	1	0	-0.639061	-8.810302	-1.258133
249	1	0	1.820537	-9.085843	-1.705895
250	1	0	1.531719	-8.083377	-0.288622
251	1	0	2.210035	-7.065483	-3.104707
252	1	0	3.292941	-7.073108	-1.713686
253	1	0	1.833929	-5.505841	-0.505201
254	1	0	6.278714	-0.576085	-0.705561
255	1	0	5.540040	-1.524723	-4.548360
256	1	0	3.899435	-1.181778	-3.942296
257	1	0	5.108559	0.107299	-4.013689
258	1	0	4.216267	-3.274728	-3.561342
259	1	0	5.598834	-5.336955	-3.747914
260	1	0	6.497624	-4.885344	-2.278953
261	1	0	6.657402	-3.909038	-3.749942
262	1	0	-4.431503	4.732271	-1.137671
263	1	0	6.012816	-2.510504	1.208376
264	1	0	4.878315	4.526238	-1.073822
265	1	0	-2.848732	-5.077905	2.145722
266	1	0	-5.750255	3.209767	1.249148
267	1	0	4.134271	-4.874003	-1.070590
268	1	0	2.999928	5.786340	0.916655
269	1	0	-4.917917	-4.241795	0.160941
270	1	0	4.702916	2.035452	0.292626
271	1	0	-1.902681	4.504347	0.497769

Counter-clockwise alternating dimer

Center Number	Atomic Number	Atomic Type	Coordinates (Angstroms) X	Y	Z
1	47	0	0.401803	0.752274	1.247958
2	6	0	5.743129	-3.010519	2.560316
3	6	0	7.752657	-1.564778	3.017026
4	6	0	5.409451	-0.590945	3.144499
5	6	0	6.011837	0.823458	3.089755
6	6	0	7.420446	0.866189	3.704125
7	6	0	8.336335	-0.144540	2.992686
8	6	0	4.665231	2.864323	2.905375
9	6	0	3.683901	3.885799	3.509438
10	6	0	1.341269	4.226080	2.863905
11	6	0	0.374650	4.563515	1.717652
12	6	0	-0.950600	3.781834	1.825576
13	6	0	-2.016638	4.887973	1.718805
14	6	0	-1.277021	6.080692	2.342948
15	6	0	-4.171368	3.837853	1.475655
16	6	0	-5.688900	3.915334	1.698220
17	6	0	-6.280779	1.816941	2.809319
18	6	0	-7.005766	0.481061	2.675483
19	6	0	-6.044774	-0.702221	2.913098
20	6	0	-6.810065	-2.036876	2.831577
21	6	0	-8.189228	0.441749	3.666013
22	6	0	-7.981163	-2.083409	3.828185
23	6	0	-8.931882	-0.899015	3.595073
24	6	0	-5.598247	-3.918980	1.863282
25	6	0	-4.839845	-5.255972	2.020337
26	6	0	-2.415399	-4.946225	2.162011
27	6	0	-1.112331	-4.793612	1.366278
28	6	0	0.104699	-4.589587	2.272287
29	6	0	1.241848	-4.877982	1.289884
30	6	0	0.652895	-6.141569	0.620646
31	6	0	3.599224	-5.044575	0.919619
32	6	0	5.071842	-5.081517	1.384317
33	6	0	5.910537	-5.964433	0.457106
34	6	0	4.662836	1.655334	5.065589
35	6	0	4.445566	5.162455	3.910116
36	6	0	2.777765	-5.254120	3.286589
37	6	0	-5.660915	-6.384229	1.385199
38	6	0	-5.407125	-3.520383	4.318463
39	6	0	-3.658773	4.892837	3.666691
40	6	0	-6.281170	4.800056	0.586220
41	6	0	5.442475	-0.935664	-4.420780
42	6	0	5.537556	6.547114	-0.812487
43	6	0	3.130144	6.335595	-3.652960
44	6	0	-5.178286	5.063418	-5.455824
45	6	0	-4.348093	1.325703	-4.731879
46	6	0	-5.447181	-5.614369	-2.707338
47	6	0	-2.588564	-4.210414	-4.957256
48	6	0	6.276132	-4.905761	-3.769292
49	6	0	-4.202976	4.217195	-4.629572
50	6	0	-4.998160	3.422746	-3.567266
51	6	0	-6.977764	0.381839	-3.375406
52	6	0	-5.933543	1.335513	-2.757413
53	6	0	-5.142296	0.436430	-1.753226

54	6	0	-5.907881	-0.903642	-1.779066
55	6	0	-5.196304	-1.970130	-2.620572
56	6	0	-4.655908	-4.318574	-2.928004
57	6	0	-3.176079	-4.570918	-2.574922
58	6	0	0.583036	-7.163576	-3.696435
59	6	0	-0.766862	-6.516133	-4.027448
60	6	0	-0.919874	-5.166321	-3.302261
61	6	0	0.242323	-4.205971	-3.627612
62	6	0	1.746975	-6.224424	-4.030738
63	6	0	1.605344	-4.853491	-3.317531
64	6	0	2.779186	-3.973484	-3.758835
65	6	0	5.244962	-3.777023	-3.641945
66	6	0	5.751340	-2.706933	-2.662185
67	6	0	7.347541	0.574861	-2.519600
68	6	0	6.255577	-0.417295	-2.093648
69	6	0	5.158322	0.519581	-1.565077
70	6	0	5.949402	1.761731	-1.098008
71	6	0	5.458169	3.026818	-1.813287
72	6	0	4.845501	5.379308	-1.532890
73	6	0	3.322796	5.494843	-1.331384
74	6	0	-0.716868	7.929755	-1.781901
75	6	0	-2.119178	4.586004	-3.374776
76	6	0	-1.663029	7.047071	-2.613893
77	6	0	-1.253194	5.561905	-2.562507
78	6	0	0.229226	5.384989	-2.961737
79	6	0	1.134239	6.263140	-2.081606
80	6	0	0.751802	7.747223	-2.193574
81	6	0	-0.067484	-2.153144	2.577366
82	6	0	3.169448	-0.088736	-0.213047
83	6	0	1.310257	-0.146948	-3.541241
84	6	0	2.287193	-0.426951	-2.581694
85	6	0	0.306888	0.766311	-3.228543
86	6	0	2.192422	0.179785	-1.330502
87	6	0	0.264120	1.297677	-1.936033
88	6	0	-0.710403	-0.748570	6.036800
89	6	0	-0.586453	-1.664162	4.992188
90	6	0	-0.427095	0.590773	5.787901
91	6	0	6.326626	-1.603495	2.423229
92	6	0	-0.057340	0.976883	4.496946
93	6	0	-0.203829	-1.196297	3.732660
94	7	0	-6.312090	2.605461	1.693252
95	7	0	0.036538	0.104973	3.480199
96	7	0	-5.915454	-3.205933	2.977481
97	7	0	-3.518474	-5.140997	1.409138
98	7	0	2.646991	4.164093	2.520523
99	7	0	5.099829	1.835832	3.677831
100	7	0	1.164444	0.985764	-0.993126
101	7	0	2.591909	-5.034149	1.845108
102	7	0	5.583375	-3.711163	1.402236
103	7	0	2.570038	6.024707	-2.332474
104	7	0	5.307981	4.105794	-1.008373
105	7	0	5.801771	-1.398750	-3.070873
106	7	0	3.964490	-4.284172	-3.155704
107	7	0	-2.253428	-4.582296	-3.576993
108	7	0	-5.194002	-3.227232	-2.125356
109	7	0	-5.119688	2.072498	-3.725974
110	7	0	-3.238598	5.087612	-3.962033
111	7	0	-3.343655	4.571913	2.265974
112	8	0	-2.881808	-4.814607	-1.396316

113	8	0	2.686635	-3.126956	-4.654920
114	8	0	-5.185475	0.939170	-0.422129
115	8	0	-5.526239	4.056316	-2.650288
116	8	0	-1.794582	3.400493	-3.496437
117	8	0	-7.176850	-0.624837	-2.385464
118	8	0	2.842421	5.148039	-0.242825
119	8	0	5.272539	3.031859	-3.033872
120	8	0	4.490236	-0.125695	-0.453893
121	8	0	6.076899	-3.055472	-1.522445
122	8	0	7.319729	1.533531	-1.465055
123	8	0	0.172055	-3.379292	3.047190
124	8	0	-1.044096	2.800728	0.793832
125	8	0	0.918150	4.028147	4.011780
126	8	0	5.044439	3.038767	1.736662
127	8	0	-0.169893	-1.841914	1.404346
128	8	0	2.778023	-0.312518	0.918291
129	8	0	-3.728605	3.211797	0.488500
130	8	0	-5.763086	2.188465	3.864737
131	8	0	-5.928691	-3.553060	0.724231
132	8	0	-2.414328	-4.857378	3.395510
133	8	0	3.342724	-4.975003	-0.291484
134	8	0	5.461368	-3.476680	3.667556
135	8	0	-0.780119	-6.006040	0.678230
136	8	0	0.031120	5.965780	1.791314
137	8	0	-4.718090	-1.689742	-3.730422
138	1	0	7.704358	-1.936446	4.048611
139	1	0	8.406632	-2.249749	2.463097
140	1	0	4.422989	-0.582468	2.670853
141	1	0	5.286182	-0.930903	4.179725
142	1	0	6.098546	1.111798	2.042028
143	1	0	7.377749	0.623656	4.775474
144	1	0	7.829754	1.879986	3.618898
145	1	0	9.328300	-0.140316	3.459866
146	1	0	8.481289	0.174713	1.950472
147	1	0	3.158264	3.490143	4.377344
148	1	0	0.846008	4.398748	0.748170
149	1	0	-1.033435	3.308317	2.810405
150	1	0	-2.159312	5.100177	0.654378
151	1	0	-1.682917	7.051430	2.046354
152	1	0	-1.241763	6.026292	3.438168
153	1	0	-5.909480	4.363270	2.665857
154	1	0	-7.402817	0.395603	1.655376
155	1	0	-5.572175	-0.578586	3.895990
156	1	0	-5.252227	-0.690904	2.155815
157	1	0	-7.218912	-2.124887	1.822336
158	1	0	-8.879060	1.267607	3.452451
159	1	0	-7.798394	0.608028	4.677839
160	1	0	-8.518702	-3.033457	3.719411
161	1	0	-7.607964	-2.040765	4.860202
162	1	0	-9.408730	-1.003037	2.610185
163	1	0	-9.739684	-0.919786	4.336260
164	1	0	-4.664969	-5.496905	3.066335
165	1	0	-1.226242	-3.973866	0.646653
166	1	0	0.053057	-5.352704	3.050205
167	1	0	1.289713	-4.072300	0.553371
168	1	0	0.971667	-6.242718	-0.418221
169	1	0	0.951375	-7.043934	1.170493
170	1	0	5.157733	-5.449907	2.404668
171	1	0	5.924955	-5.552589	-0.553530

172	1	0	5.504731	-6.980984	0.417145
173	1	0	6.937367	-6.014127	0.831473
174	1	0	3.629149	1.295669	5.128937
175	1	0	4.755421	2.581687	5.638315
176	1	0	5.306725	0.914888	5.540043
177	1	0	5.200439	4.947166	4.675338
178	1	0	4.954505	5.588998	3.039953
179	1	0	3.745662	5.901557	4.310187
180	1	0	3.751379	-4.883869	3.606532
181	1	0	2.034343	-4.677994	3.837707
182	1	0	2.669450	-6.313968	3.551651
183	1	0	-5.103835	-7.324381	1.434799
184	1	0	-5.896031	-6.157783	0.342972
185	1	0	-6.607654	-6.511679	1.921621
186	1	0	-5.365283	-2.595659	4.898220
187	1	0	-4.394517	-3.923402	4.268039
188	1	0	-6.057710	-4.224445	4.852071
189	1	0	-3.963011	5.938414	3.792625
190	1	0	-4.439466	4.222102	4.026904
191	1	0	-2.771523	4.710682	4.279274
192	1	0	-7.364182	4.872658	0.725151
193	1	0	-6.068940	4.396975	-0.408413
194	1	0	-5.860791	5.810310	0.641148
195	1	0	6.255697	-1.106543	-5.136230
196	1	0	4.536590	-1.430170	-4.776770
197	1	0	5.248928	0.138829	-4.383293
198	1	0	6.622574	6.465994	-0.924363
199	1	0	5.214352	7.507949	-1.227872
200	1	0	5.289327	6.539726	0.254171
201	1	0	3.868403	7.143859	-3.606379
202	1	0	2.322650	6.664882	-4.306903
203	1	0	3.591530	5.451804	-4.104035
204	1	0	-5.869825	4.418852	-6.007705
205	1	0	-5.772651	5.706851	-4.798490
206	1	0	-4.634896	5.682919	-6.176447
207	1	0	-4.718581	1.513649	-5.746983
208	1	0	-4.438590	0.259282	-4.523989
209	1	0	-3.291376	1.599627	-4.673748
210	1	0	-5.086822	-6.399649	-3.381101
211	1	0	-5.331445	-5.972767	-1.681804
212	1	0	-6.508772	-5.444251	-2.909853
213	1	0	-1.662407	-4.062813	-5.513601
214	1	0	-3.168090	-4.986756	-5.472311
215	1	0	-3.143379	-3.269813	-4.971132
216	1	0	7.219898	-4.521267	-4.172318
217	1	0	6.485530	-5.358811	-2.796840
218	1	0	5.897472	-5.675447	-4.448229
219	1	0	-3.633049	3.564485	-5.287541
220	1	0	-7.943317	0.866399	-3.544295
221	1	0	-6.630538	-0.056316	-4.318504
222	1	0	-6.445959	2.090463	-2.162605
223	1	0	-4.102623	0.311850	-2.077160
224	1	0	-6.099905	-1.266114	-0.766497
225	1	0	-4.769692	-4.007099	-3.963227
226	1	0	0.612094	-7.420168	-2.627872
227	1	0	0.693824	-8.106012	-4.246231
228	1	0	-1.591329	-7.178079	-3.736326
229	1	0	-0.839662	-6.369613	-5.114482
230	1	0	-0.921359	-5.355123	-2.226065

231	1	0	0.241088	-3.922330	-4.686162
232	1	0	0.122246	-3.279552	-3.052978
233	1	0	2.701187	-6.686015	-3.754256
234	1	0	1.779083	-6.052507	-5.116139
235	1	0	1.698947	-5.020635	-2.236614
236	1	0	5.044580	-3.363520	-4.627667
237	1	0	8.344268	0.127331	-2.539584
238	1	0	7.141931	1.048497	-3.487705
239	1	0	6.630460	-0.985956	-1.242792
240	1	0	4.438937	0.788974	-2.336700
241	1	0	5.905810	1.876541	-0.014582
242	1	0	5.113198	5.381779	-2.587950
243	1	0	-0.817612	7.671414	-0.718216
244	1	0	-1.009874	8.981336	-1.881098
245	1	0	-2.686881	7.178303	-2.237622
246	1	0	-1.655828	7.391620	-3.657800
247	1	0	-1.349714	5.213632	-1.522482
248	1	0	0.344950	5.652308	-4.021803
249	1	0	0.508188	4.332645	-2.863745
250	1	0	0.988040	5.967326	-1.041338
251	1	0	1.403883	8.346721	-1.547828
252	1	0	0.893422	8.107415	-3.222406
253	1	0	1.353868	-0.644255	-4.505090
254	1	0	3.084523	-1.126719	-2.798983
255	1	0	-0.448702	1.067767	-3.945183
256	1	0	-0.514198	1.996415	-1.649427
257	1	0	-1.016305	-1.080650	7.024381
258	1	0	-0.796979	-2.717894	5.127700
259	1	0	-0.492366	1.339987	6.570117
260	1	0	6.375834	-1.333030	1.360182
261	1	0	0.182894	2.014388	4.282468
262	1	0	-6.446639	2.145739	0.797211
263	1	0	-3.439521	-5.094071	0.389417
264	1	0	2.930876	4.400817	1.568257
265	1	0	5.783146	-3.284042	0.500646
266	1	0	5.272486	3.956901	-0.000962
267	1	0	3.947701	-4.762585	-2.255493
268	1	0	-5.483102	-3.409841	-1.162865
269	1	0	-3.581913	5.987338	-3.657560
270	1	0	-4.625371	1.739653	-0.346397
271	1	0	-2.004509	2.727344	0.576385
